# A new conceptual model of global ocean heat uptake

**DOI:** 10.1007/s00382-023-06989-z

**Published:** 2023-12-22

**Authors:** Jonathan M. Gregory, Jonah Bloch-Johnson, Matthew P. Couldrey, Eleftheria Exarchou, Stephen M. Griffies, Till Kuhlbrodt, Emily Newsom, Oleg A. Saenko, Tatsuo Suzuki, Quran Wu, Shogo Urakawa, Laure Zanna

**Affiliations:** 1 NCAS, University of Reading, Reading, UK; 2 Met Office Hadley Centre, Exeter, UK; 3 Barcelona Supercomputing Centre, Barcelona, Spain; 4 NOAA Geophysical Fluid Dynamics Laboratory, Princeton, NJ USA; 5 Atmospheric and Oceanic Sciences Program, Princeton University, Princeton, NJ USA; 6 Courant Institute, New York University, New York City, NY USA; 7 SEOS, University of Victoria, Victoria, BC Canada; 8 Japan Agency for Marine Earth Science and Technology, Yokohama, Japan; 9 Meteorological Research Institute, Tsukuba, Japan

**Keywords:** Ocean heat uptake, Atlantic meridional overturning circulation, Effective climate sensitivity, Transient climate response

## Abstract

We formulate a new conceptual model, named “*MT*2”, to describe global ocean heat uptake, as simulated by atmosphere–ocean general circulation models (AOGCMs) forced by increasing atmospheric CO_2_, as a function of global-mean surface temperature change *T* and the strength of the Atlantic meridional overturning circulation (AMOC, *M*). *MT*2 has two routes whereby heat reaches the deep ocean. On the basis of circumstantial evidence, we hypothetically identify these routes as low- and high-latitude. In low latitudes, which dominate the global-mean energy balance, heat uptake is temperature-driven and described by the two-layer model, with global-mean *T* as the temperature change of the upper layer. In high latitudes, a proportion *p* (about 14%) of the forcing is taken up along isopycnals, mostly in the Southern Ocean, nearly like a passive tracer, and unrelated to *T*. Because the proportion *p* depends linearly on the AMOC strength in the unperturbed climate, we hypothesise that high-latitude heat uptake and the AMOC are both affected by some characteristic of the unperturbed global ocean state, possibly related to stratification. *MT*2 can explain several relationships among AOGCM projections, some found in this work, others previously reported: $\bullet$ Ocean heat uptake efficiency correlates strongly with the AMOC. $\bullet$ Global ocean heat uptake is not correlated with the AMOC. $\bullet$ Transient climate response (TCR) is anticorrelated with the AMOC. $\bullet$ *T* projected for the late twenty-first century under high-forcing scenarios correlates more strongly with the effective climate sensitivity than with the TCR.

## Introduction

### Global-mean energy balance

The global-mean energy balance of the climate system, often called the “Earth energy balance”, has proved to be a useful framework for quantitative comparison among climate models, and between models and observations, regarding the rate and magnitude of global climate change. The energy balance may be written

1 $$\begin{aligned} N = F - \alpha T, \end{aligned}$$

where *N* is the rate of energy storage in the Earth system (W m^-2^), *F* is the effective radiative forcing (ERF, W m^-2^, the perturbation caused by agents such as greenhouse gases), *T* is the surface air temperature change (K), and $\alpha$ is the climate feedback parameter (W m^-2^ K^-1^). *F*, *N* and *T* are all global means over the Earth surface area. (Appendix E repeats key equations for reference, and Appendix F tabulates the definitions of the symbols and abbreviations that we use repeatedly throughout this paper.)

*F*, *N* and *T* are defined as differences from an assumed unperturbed steady state in which they are all zero. When a positive *F* is imposed, *T* increases, and the perturbed climate system radiates an energy flux $\alpha T$ to space, which opposes *F*. For the system to be stable to small perturbations, $\alpha$ must be positive. In some other papers, $\alpha$ is a negative number, with $+\alpha T$ in Eq. (1).

In a steady state, the energy content of the system is not changing, whence $N=0$. Therefore, if $F_{2\times }$ is the ERF due to doubling atmospheric CO_2_ concentration, the steady state for $2\times \text{ CO}_{2}$ has $T=F_{2\times }/\alpha$ from Eq. (1). This quantity is the *equilibrium* climate sensitivity. When $\alpha$ is determined from *F*, *T* and *N* through Eq. (1) from any transient state, in which $N\ne 0$, the quantity $F_{2\times }/\alpha$ is the *effective* climate sensitivity. The effective and equilibrium climate sensitivity differ, in any AOGCM, because $\alpha$ is not a constant, due to the dependence of climate feedbacks on several factors, including global-mean and geographical patterns of surface temperature change, the magnitude of *F* and the nature of the forcing agents (Andrews et al. 2015; Marvel et al. 2016; Gregory et al. 2020; Bloch-Johnson et al. 2021; Andrews et al. 2022; Salvi et al. 2022). Moreover, $\alpha$ has a wide spread among AOGCMs, and there is a large uncertainty in its real-world value (Andrews et al. 2012; Zelinka et al. 2020; Sherwood et al. 2020).

### The zero-layer model of transient climate change and ocean heat uptake

Studies of climate change simulated by atmosphere-ocean general circulation models (AOGCMs) have found that, for timescales longer than about a year, energy storage can be disregarded other than the heat content (strictly, the enthalpy) of the ocean (Palmer and McNeall 2014). In that case, *N* is entirely absorbed by the ocean.

In many scenarios of future change, *F* increases continuously in time. After the first decade under such a regime, heat storage in the upper ocean layer (a few tens of metres) is unimportant, and *T* can be regarded as though it were the temperature of a surface skin with zero heat capacity. Practically all of *N* is then stored in the deep ocean, whose heat capacity is vast, and whose temperature change is consequently negligible for many decades.

This being so, *T* can be modelled by the “zero-layer model” for times longer than about a decade and shorter than about a century (Bouttes et al. 2013):

2 $$\begin{aligned} T = \frac{F}{\alpha +\kappa } \qquad \text{ and } \qquad N = \kappa T = \frac{\kappa F}{\alpha +\kappa }. \end{aligned}$$

In the zero-layer model, *F* is always balanced by the sum of increased heat loss, $\alpha T$, to space and downward transfer, $\kappa T$, into the deep ocean. The quantity $\kappa$ is the AOGCM-specific thermal coupling coefficient between the upper and deep ocean, called the ocean heat uptake efficiency (OHUE), whose units are the same as for $\alpha$ (W per m^2^ of world area per K; we choose the positive-stable convention for $\alpha$ so that it has the same sign as $\kappa$). $F=\alpha T + \kappa T$ gives the diagnostic relationships for *T* and *N* (Eq. 2). Because no heat capacity is involved (hence the name “zero-layer” for the model), *T* and *N* respond instantaneously, both increasing like *F*.

The standard metric for global warming under increasing CO_2_ is the transient climate response (TCR), defined as *T* at the time of $2\times \text{ CO}_{2}$ in the idealised “1pctCO2” scenario, with atmospheric CO_2_ concentration increasing at 1% yr^-1^. From Eq. (2) it follows that $\text{ TCR }=F_{2\times }/(\alpha +\kappa )$, and hence that TCR is anticorrelated with both $\alpha$ and $\kappa$, considering the variation in these quantities across a set of AOGCMs.

Although not formally required by the zero-layer model, it is often implicitly assumed that $\alpha$ and $\kappa$ are independent variables, which respectively quantify the atmosphere and ocean response to forcing. Gregory and Forster (2008) found $\alpha$ and $\kappa$ to be uncorrelated across AOGCMs of CMIP3, the third phase of the Coupled Model Intercomparison Project (CMIP), and Kuhlbrodt and Gregory (2012) the same for CMIP5 (the fifth phase; note that no “CMIP4” exists), after excluding two outlying AOGCMs. Absence of correlation suggests that climate feedback and ocean heat uptake are independent phenomena that together determine the TCR in the zero-layer model.

OHUE, defined as $\kappa \equiv N/T$, is a constant in the zero-layer model. The zero-layer model is a special approximate solution of the two-layer ocean model, which we describe later (Sect. 3.1.2). OHUE is not constant in the two-layer model or in AOGCMs (Sect. 4; Gregory et al. 2015).

OHUE is conventionally evaluated as *N*/*T* at the time of $2\times \text{ CO}_{2}$ in the 1pctCO2 scenario (*viz.* after 70 years). Its large spread, spanning a factor of two among AOGCMs (Kuhlbrodt and Gregory 2012), implies uncertainty in projections of *T* under any given forcing scenario. The uncertainty motivates the need to understand and to constrain the spread in OHUE.

In the 1pctCO2 scenario, *F* rises linearly in time, since the CO_2_ concentration rises exponentially, if CO_2_ ERF depends logarithmically on concentration, as is usually assumed. [Small deviations from this behaviour are found in atmosphere radiative transfer calculations and GCMs e.g. Byrne and Goldblatt (2014), Bloch-Johnson et al. (2021).] According to Eq. (2), we expect $T(t)\propto F(t)\propto t$ and $N(t) \propto F(t) \propto t$ in 1pctCO2 for any given AOGCM, where *t* is time. These proportionalities are found to hold quite accurately for the first 70 years, up to the time of $2\times \text{ CO}_{2}$ (Gregory et al. 2015).

In the zero-layer model, the deep ocean accumulates all the heat added to the climate system

3 $$\begin{aligned} H(t)={\mathcal {A}}\int _{0}^{t}\,N(t')\,\textrm{d}t', \end{aligned}$$

where ${\mathcal {A}}=5.101\times 10^{14}$ m^2^ is the global surface area and *H* is the global ocean heat uptake (OHU, in $\text{ ZJ }\equiv 10^{21}$J), which is a function of time. In 1pctCO2, $N(t)\propto t \Rightarrow N(t')=N(t)t'/t$, whence

4 $$\begin{aligned} H(t)={\textstyle \frac{1}{2}}{\mathcal {A}} \, t \, N(t)={\textstyle \frac{1}{2}}{\mathcal {A}} \, t\,\kappa \,T(t)=\frac{{\mathcal {A}}\,t\,\kappa }{2(\alpha +\kappa )}F(t) \end{aligned}$$

(see also Appendix B.1).

Making the assumption that $\alpha$ and $\kappa$ are unrelated, we can consider a set of hypothetical AOGCMs with the same $\kappa$ and a range of $\alpha$, which produces a range of *T* for given *t* by Eq. (2). Hence $H={\textstyle \frac{1}{2}}{\mathcal {A}}t\kappa T\propto T$ from Eq. (4). Furthermore, a set of AOGCMs with the same $\alpha$ and a range of $\kappa$ will have $H={\textstyle \frac{1}{2}}{\mathcal {A}}tF\kappa /(\alpha +\kappa )\propto \kappa /(\alpha +\kappa )$ for given *t*, which indicates a correlation between *H* and $\kappa$. The correlation is positive because $\kappa$ is about half the size of $\alpha$ in AOGCMs, so $\kappa$ has less importance in the denominator than in the numerator (Kuhlbrodt and Gregory 2012). Thus the zero-layer model predicts that larger ocean heat uptake efficiency $\kappa$ and larger global warming *T* should both give larger ocean heat uptake *H*.

### Plan and purpose of this paper

We proceed in Sect. 2 by examining relationships across AOGCMs among OHUE, OHU, *T* and other quantities in the $2\times \text{ CO}_{2}$ state of 1pctCO2 experiments from CMIP5 and CMIP6 (Phases 5 and 6 of CMIP, which henceforth we will refer to as “CMIP5&6”). We show that $\alpha$ and $\kappa$ are positively correlated across AOGCMs, contradicting the assumption often made in connection with the zero-layer model (Sect. 2.2). We show that OHU and OHUE are not correlated across AOGCMs, unlike in the zero-layer model (Sect. 2.3). We find moreover that, although OHU is correlated with *T* as predicted by the zero-layer model, some part of the OHU is unrelated to *T* i.e. OHU is substantial even for small *T* (Sect. 2.4). Furthermore, the zero-layer model does not incorporate the strong correlation of OHUE with the Atlantic meridional overturning circulation (AMOC) (Kostov et al. 2014; Winton et al. 2014), which is evident in AOGCM data (Sect. 2.6) but has not been explained. From the above evidence, it appears that the zero-layer model is inadequate for modelling AOGCM OHU, despite its successful use in modelling *T*.

On the basis of these and other findings (summarised in Sect. 2.12), in Sect. 3 we propose a new conceptual model of OHU, called “*MT*2”. In the *MT*2 model, part of the OHU is linearly related to the strength of the AMOC in the unperturbed state, although the AMOC itself is not the dominant physical mechanism of the OHU, which mostly takes place in the Southern Ocean. We offer a physical interpretation of the *MT*2 model, and show that it gives an accurate reproduction of OHU as a function of time in individual AOGCMs and the AOGCM mean in both the 1pctCO2 and the abrupt4xCO2 scenario (constant quadrupled CO_2_ concentration).

In the two subsequent sections, we use the *MT*2 model to explain how OHUE (Sect. 4) and *T* (Sect. 5) depend on time, on the climate feedback parameter (which is itself time-dependent and AOGCM-specific) and on the unperturbed AMOC strength (AOGCM-specific), but not involving the time-dependence of AMOC during climate change.

Section 6 is a summary and discussion. In Sect. 6, we summarise the *MT*2 model (Sect. 6.1) and our new findings and explanations (Sect. 6.2). We finish with some unanswered questions and concluding remarks (Sect. 6.3).

The purpose of this work is to describe and explain the time-dependent behaviour of global ocean heat uptake given the time-dependent global-mean surface temperature change. We do not investigate the behaviour of the forcing or the climate feedback parameter, which are the subjects of a great deal of research, and which together with OHU determine *T* through Eq. (1). However, the *MT*2 model could be combined with these other elements to construct a global-mean energy-balance model.

## Ocean heat uptake efficiency in AOGCMs in the transient $2\times \text{ CO}_{2}$ state

**Fig. 1:**
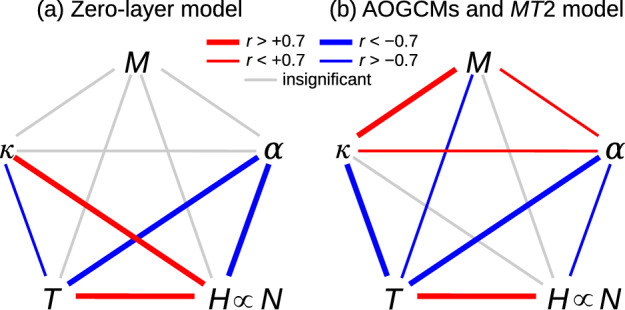
Correlations across AOGCMs among piControl AMOC strength *M*, ocean heat uptake efficiency (OHUE) $\kappa$, climate feedback parameter $\alpha$ ($\propto 1/\text{EffCS }$), transient climate response (TCR) *T*, and global ocean heat uptake *H* in the $2\times \text{ CO}_{2}$ state of 1pctCO2. *H* is proportional across AOGCMs to the rate *N* of ocean heat uptake. Red lines join quantities whose correlation is positive, blue negative. Thick lines indicate correlation coefficients of magnitude $>0.7$, thin lines weaker but significant correlations, and grey lines mean no significant correlation. **a** Correlations expected from the zero-layer model, calculated from synthetic data for the same number of AOGCMs as in our CMIP5&6 set, and with the same mean and standard deviation as CMIP5&6 $\alpha$ and $\kappa$. For the synthetic data, $\alpha$ and $\kappa$ were generated by selection from independent random normal distributions, then *T* and *H* calculated assuming the formulae of the zero-layer model (Eqs. 2 and 4). **b** Correlations diagnosed from CMIP5&6 AOGCMs and accounted for by the *MT*2 model

**Table 1:** Various quantities evaluated in 1pctCO2 and piControl experiments with CMIP5&6 AOGCMs and the CMIP3 AOGCM HadCM3

		AOGCM	*M*	TCR	$\Delta$SST	OHU	OHUE	$\alpha$
			Sv	K	K	ZJ	W m^-2^ K^-1^	
*CMIP5*								
A	14	ACCESS1-0	17.9	1.98	1.22	861	0.822	1.08
B		bcc-csm1-1		1.76	1.17	682	0.654	1.36
C	14	CNRM-CM5	15.0	2.08	1.40	661	0.521	1.05
D	14	CSIRO-Mk3-6-0	20.5	1.78	1.20	733	0.732	1.09
E*	14	CanESM2	17.1	2.41	1.57	828	0.578	1.19
F	14	GFDL-CM3	27.2	1.95	1.32	860	0.696	1.13
G		GFDL-ESM2G		1.05	0.71	631	0.912	1.58
H*	4	GFDL-ESM2M	27.7	1.34	0.90	720	0.870	1.36
J*	14	HadGEM2-ES	14.8	2.50	1.64	861	0.514	0.79
K		inmcm4	20.5	1.29				1.67
L	14	IPSL-CM5A-LR	11.5	2.04	1.40	678	0.538	0.86
M	14	IPSL-CM5A-MR	12.5	2.03	1.47			0.88
Q*	14	MPI-ESM-LR	23.5	2.06	1.32	869	0.783	1.33
R	14	MPI-ESM-MR	19.1	2.04	1.32	850	0.708	1.40
S	1	MRI-CGCM3	15.3	1.56	1.03	631	0.641	1.54
T	14	NorESM1-M	32.3	1.39	0.85	733	1.008	1.63
V	1	NorESM1-ME	32.0		0.93	721	0.908	
Y	14	ACCESS1-3	19.2	1.65	1.15	785	0.793	1.17
Z	14	MPI-ESM-P	22.5	2.06	1.34	846	0.723	1.50
*CMIP6*								
a	14	ACCESS-CM2	20.8	2.11	1.35	905	0.737	
b	14	ACCESS-ESM1-5	22.1	1.95	1.34	838	0.677	
d	1	CESM2	24.6	2.07	1.44	970	0.753	1.15
e	14	CNRM-CM6-1	17.0	2.13	1.49	828	0.600	0.90
f	14	CNRM-ESM2-1	19.1	1.84	1.25	754	0.697	0.48
g	1	GFDL-CM4	19.5	2.06	1.33	771	0.678	1.48
h*	1	HadGEM3-GC31-LL	16.9	2.55	1.69	1016	0.620	0.83
i	1	IPSL-CM6A-LR	13.2	2.29	1.63	795	0.516	0.99
k*	14	MPI-ESM1-2-HR	19.7	1.66	1.06	765	0.819	1.53
m*		MIROC6	18.7	1.55	1.01	656	0.716	1.63
n	14	NorESM2-LM	23.6	1.48	1.00	601	0.677	2.07
q*	14	CanESM5	13.1	2.74	1.74	880	0.546	0.68
t*	14	MRI-ESM2-0	21.5	1.64	1.12	773	0.780	1.33
u	14	UKESM1-0-LL	16.2	2.79	1.87	1024	0.609	0.82
*CMIP3*								
x*		HadCM3	19.0	1.97	1.26	729	0.614	1.25

**Fig. 2:**
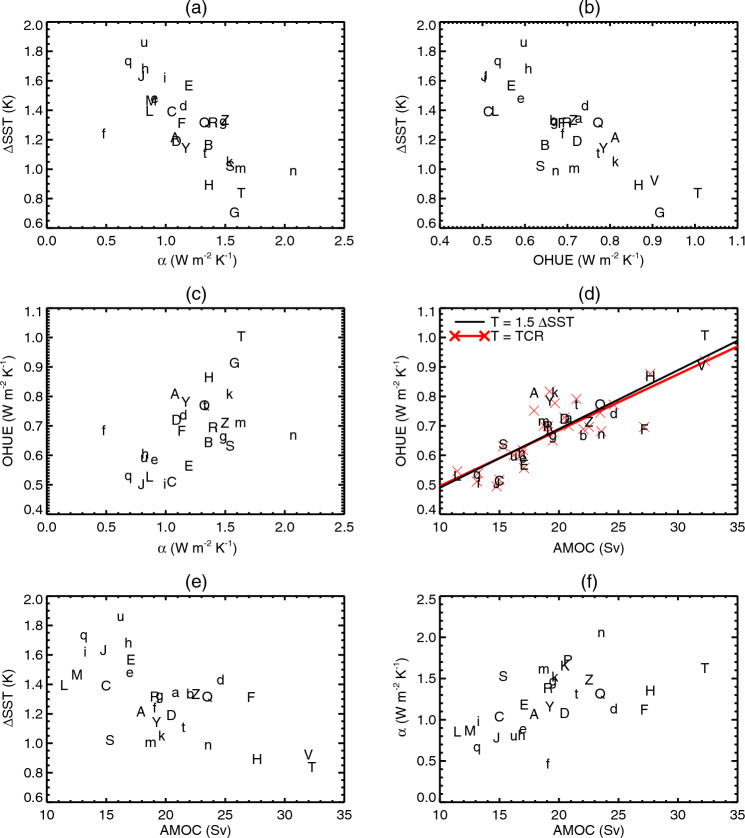
Relationships across CMIP5&6 AOGCMs among various quantities: global-mean sea-surface temperature change $\Delta$SST and ocean heat uptake efficiency (OHUE) in the $2\times \text{ CO}_{2}$ state of 1pctCO2, the climate feedback parameter $\alpha$ from the first 20 years of abrupt4xCO2, and the strength of the Atlantic meridional overturning circulation (AMOC) in piControl. All panels use the letters shown in Table 1 to identify the AOGCMs, CMIP5 with upper-case letters, CMIP6 lower-case. The lines in **d** show ordinary least-squares regressions

**Fig. 3:**
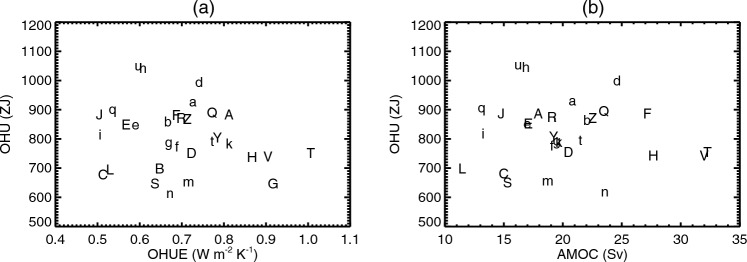
Scatter plots showing no significant relationship across CMIP5&6 AOGCMs between global ocean heat uptake (OHU) in the $2\times \text{ CO}_{2}$ state of 1pctCO2 experiments and **a** ocean heat uptake efficiency (OHUE) in the same state, **b** the strength of the Atlantic meridional overturning circulation (AMOC) in piControl. Both panels use the letters shown in Table 1 to identify the AOGCMs, CMIP5 with upper-case letters, CMIP6 lower-case

The letters in the first column are used as identification in Figs. 2 and 3, and * indicates FAFMIP models. In the second column, “1” indicates that 1pctCO2 is analysed and “4” indicates abrupt4xCO2 is analysed. The other columns are the strength of the Atlantic meridional overturning circulation in piControl (AMOC, *M*, time-mean of years 1–70), global-mean surface air temperature change (TCR), global-mean sea surface temperature change ($\Delta$SST), global ocean heat uptake (OHU), ocean heat uptake efficiency (OHUE), climate feedback parameter ($\alpha$, from years 1–20 of abrupt4xCO2). TCR, $\Delta$SST, OHU and OHUE are all for the $2\times \text{ CO}_{2}$ state of 1pctCO2. OHUE is calculated using TCR. See Appendix A for further details of calculations. CMIP6 values for TCR and $\alpha$ were evaluated by Mark Ringer (available at https://github.com/mark-ringer/cmip6 under the Creative Commons Attribution-ShareAlike 4.0 International Public License). Unspecified values arise from unavailability of diagnostics or other technical difficulties

In this section we analyse data from the piControl and $2\times \text{ CO}_{2}$ state of 1pctCO2 experiments of CMIP5& AOGCMs (Table 1). Our aim is to test the zero-layer model (Sect. 1.2) as a description of the relationships across the CMIP5&6 AOGCMs between quantities relevant to global ocean heat uptake (OHU), and to discover any relationships that the zero-layer model does not account for, by evaluating correlation coefficients and other statistics. Correlations expected on the basis of the zero-layer model are depicted in Fig. 1a. Although correlations are no proof of a causal connection, a satisfactory physical interpretation of the AOGCM data must account for their presence. Furthermore, the *absence* of an expected correlation can falsify a physical hypothesis.

In Sect. 2.12 we summarise our findings about the statistical relationships among AOGCM quantities, depicted in Fig. 1b. These findings provide the starting-point for the development of a new conceptual model of ocean heat uptake, which we present in Sect. 3.

### AOGCM diagnostics

For the strength of the Atlantic meridional overturning circulation (AMOC, *M*) we use the maximum of the piControl time-mean depth–meridional overturning streamfunction in the Atlantic north of 30^∘^ N and within 500–2000 m depth. See Appendix A.1 for comparison with the AMOC at 26^∘^N.

Ocean heat uptake efficiency (OHUE) is usually evaluated from the top-of-atmosphere net downward radiative flux *N* and OHU usually as the global and time-integral of *N*. We instead use the 3D ocean temperature change for OHU, because about 10% of the added heat is stored outside the ocean, and because a few atmosphere GCMs do not conserve energy accurately enough for our purposes (Hobbs et al. 2016). We calculate *N* from the global-mean time-derivative of OHU as per Eq. (3). In Appendix A3–A4, we show that our OHU and OHUE for most AOGCMs agree well with other definitions.

In this section, because we are concentrating on ocean quantities, we use global-mean sea-surface temperature change $\Delta$SST at the time of $2\times \text{ CO}_{2}$ to estimate the transient climate response (TCR), which is defined as global-mean surface air temperature change *T* in the same state. TCR and $\Delta$SST are highly correlated ($r=0.98$ for 24 AOGCMs). They are almost proportional with $\text{ TCR }\simeq 1.5\times {\Delta \textrm{SST}}$ (Appendix A.2; Toda et al. 2021). Consequently OHUE ($=N/T$) is very similar for the two estimates of *T* (compare the black letters and red crosses in Fig. 2d).

Except where otherwise stated, reported values for the product-moment correlation coefficient, *r*, are statistically significant at the 5% level. This assertion means that the probability is only 5% of the correlation between the two variables equalling or exceeding our estimated *r* if the two variables are actually independent (in accordance with the null hypothesis).

### TCR, EffCS and OHUE are all correlated

In the $2\times \text{ CO}_{2}$ state of 1pctCO2, AOGCMs with larger $\alpha$ (equivalent to smaller effective climate sensitivity $\text{ EffCS }\propto 1/\alpha$) have smaller $\Delta$SST ($r=-0.72$, or $r=-0.82$ excluding the outliers CNRM-ESM2-1 and NorESM2-LM, marked “f” and “n” respectively, in Fig. 2a). Likewise, AOGCMs with larger OHUE $\kappa$ have smaller $\Delta$SST ($r=-0.79$, Fig. 2b). These correlations are consistent with the zero-layer model $T=\text{ TCR }=F_{2\times }/(\alpha +\kappa )$ (Eq. 2).

Moreover, $\alpha$ and $\kappa$ are correlated too ($r=0.53$, or $r=0.67$ excluding CNRM-ESM2-1 and NorESM2-LM, in Fig. 2c). This correlation means that climate feedback and ocean heat uptake are not entirely independent in their effects on TCR, despite the frequent tacit assumption to the contrary, and it demands a physical explanation, to which we will return later.

Previous work (Gregory and Forster 2008; Kuhlbrodt and Gregory 2012) found no correlation between $\alpha$ and $\kappa$. Our analysis differs in revealing a relationship mainly for two reasons, relating to methodology. First, we have a larger set of AOGCMs, with the inclusion of CMIP6. Second, we evaluate $\alpha$ from years 1–20 of abrupt4xCO2 and $\kappa$ from years 61–80 of 1pctCO2. During these 20-year periods $\alpha$ and $\kappa$ have nearly constant values in any given AOGCM, whereas the previous works used regression slopes to fit $\textrm{d}(F-N)/\textrm{d}T$ and $\textrm{d}N/\textrm{d}T$ respectively for $\alpha$ and $\kappa$ from years 1–70 of 1pctCO2, during which they decrease in all AOGCMs (Sect. 4.3; Gregory et al. 2015). Since the rate of change differs among AOGCMs, it increases the scatter in the relationships.

### OHU is not correlated with OHUE

The zero-layer model predicts a correlation between OHU and OHUE $\kappa$ across AOGCMs at any given time in 1pctCO2 (Sect. 1.2). The idea is that AOGCMs which transport heat more efficiently from the upper to the deeper layer will store a greater fraction of the forcing *F*, for a given $\alpha$. However, the CMIP5&6 1pctCO2 experiments do not follow this prediction (Fig. 3a); they show insignificant correlation between OHU and OHUE.

### OHU is correlated with *T*, but part of OHU is unrelated to *T*

**Fig. 4:**
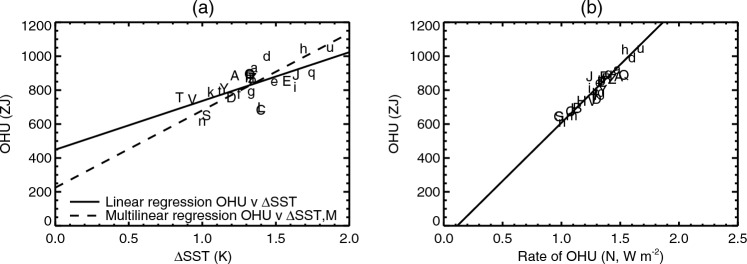
Relationships of global ocean heat uptake (OHU) to **a** global-mean sea-surface temperature change $\Delta$SST and the strength *M* of the Atlantic meridional overturning circulation in piControl, and **b** rate of ocean heat uptake, across CMIP5&6 AOGCMs in the $2\times \text{ CO}_{2}$ state of 1pctCO2. Both panels use the letters shown in Table 1 to identify the AOGCMs, CMIP5 with upper-case letters, CMIP6 lower-case. The solid lines show ordinary least-squares regressions, the dashed line in **a** shows multiple linear regression

The zero-layer model predicts that $\text{ OHU }\propto {\Delta \textrm{SST}}$ (Sect. 1.2), because larger *T* produces a greater heat flux $N=\kappa T$ from the upper to the deep layer, for a given $\kappa$. This prediction is only partially supported by CMIP5&6 experiments. There is a strong and significant correlation between OHU and $\Delta$SST (Fig. 4a, $r=0.72$; recall from Sect. 2 that $T=1.5\times {\Delta \textrm{SST}}$ to an excellent approximation). OHU also correlates with $\alpha$ ($r=-0.53$, not shown) and hence with effective climate sensitivity $\text{ EffCS }\propto 1/\alpha$, consistent with $\alpha$ being the main influence on the spread of $\Delta$SST (Fig. 2a; Sect. 5.1).

Linear regression of OHU against $\Delta$SST gives a good fit, but the relationship has a non-zero intercept of $430\pm 63$ ZJ, which is 55% of the multi-model mean OHU. This quantity of heat reaches the deep ocean without involving any substantial global-mean warming. That is, OHU is larger in models with greater *T*, but OHU is not *proportional* to *T*. The intercept in Fig. 4a suggests a hypothetical AOGCM with non-zero OHU but $T=0$ at the time of $2\times \text{ CO}_{2}$.

### OHU is proportional to the rate of OHU

The zero-layer model predicts that the OHU *H*(*t*) that has been accumulated by time *t* in 1pctCO2 is proportional to the *rate* of OHU *N*(*t*) at that time i.e. $H\propto N$ (Eq. 4). We find that the prediction $H\propto N$ holds for the CMIP5&6 AOGCMs (Fig. 4b, $r=0.93$). The ordinary least-squares regression slope of *H*(*N*) is $600\pm 40$ ${\mathrm{ZJ\,W}}^{-1}\,{\textrm{m}}^{2}$, where $1{{\mathrm{ZJ\,W}}^{-1}\,{\textrm{m}}^{2}}=10^{21}{{\textrm{m}}^{2}\,{\textrm{s}}}$, which is statistically indistinguishable from the zero-layer slope of ${\textstyle \frac{1}{2}}{\mathcal {A}}\, t=560$ ${\mathrm{ZJ\,W}}^{-1}\,{\textrm{m}}^{2}$ for $t=70$ years. (We make this inference with only medium confidence because the *H* versus *N* slope is underestimated by a probably small but unknown amount, due to unforced variability in *N* affecting the regression.)

In the zero-layer model, $H\propto N$ is a consequence of $N\propto F$ (Eq. 2) and $F\propto t$ (due to CO_2_ increasing exponentially), where *F*(*t*) is the CO_2_ ERF. Hence *N* increases linearly in time in all AOGCMs, differing among them only in its rate of increase, and $H=\int N\,\textrm{d}t$ increases quadratically in time, so the ratio *H*/*N* depends only on *t*. Thus *H*/*N* is the same in all AOGCMs at any given *t*. Although this prediction is correct, we cannot rely on the zero-layer model to explain the AOGCM behaviour, in view of its inadequacies (Sects. 2.3–2.4). In Appendix C.5 we derive $H\propto N$ using the conceptual model of Sect. 3.

### OHUE is correlated with piControl AMOC

We find that OHUE in the $2\times \text{ CO}_{2}$ state of 1pctCO2 experiments with our set of CMIP5&6 AOGCMs is strongly correlated with the strength of the AMOC in the unperturbed climate of the corresponding piControl experiments (Fig. 2d). The product-moment correlation coefficient is $r=0.83$ and the rank correlation coefficient 0.80. That is, OHUE is generally large in AOGCMs where AMOC in the piControl is strong, and OHUE is small in AOGCMs with weak piControl AMOC. Accounting for the relationship of OHUE and AMOC is one of the purposes of this work.

Winton et al. (2014) demonstrated a similar correlation between OHUE and AMOC in a set of ten AOGCMs developed by the Geophysical Fluid Dynamics Laboratory. For eight CMIP AOGCMs, Kostov et al. (2014) showed that the thermal coupling $\gamma$ between the layers of the two-layer model (described in Sect. 3.1.2) is highly correlated with *M*. Our correlation is the same phenomenon as they discovered, because $\gamma$ is the same as OHUE $\kappa$ in the zero-layer model for 1pctCO2 (Appendix B.1).

### OHU is not correlated with piControl AMOC

One hypothesis for the correlation between OHUE and AMOC is that the AMOC itself is the mechanism for a large part of OHU so that, if the AMOC is stronger, substantially more heat is conveyed by the AMOC from the surface into the deeper ocean (Kostov et al. 2014). This suggestion is reinforced by zonal-mean cross-sections of ocean temperature change (e.g. Fig. 2 of Kostov et al. 2014), in which the largest and deepest warming is apparent at high northern latitudes. However, that picture is somewhat misleading, because the area of the ocean is relatively small at the latitudes of the North Atlantic, where the AMOC has its strongest influence. The entire Atlantic (north of 30^∘^ S) accounts for about 30% of global ocean OHU below 200 m (Saenko et al. 2021). The largest OHU occurs in the Southern Ocean (e.g. Kuhlbrodt and Gregory 2012; Frölicher et al. 2015), which has much greater area. This fact makes it less likely that the correlation of the AMOC with OHUE is due to an effect of AMOC itself as a dominant mechanism of global OHU.

We find insignificant correlation of OHU with the AMOC across CMIP5&6 AOGCMs in 1pctCO2 (Fig. 3b), which is further evidence against AMOC influence on OHU. Furthermore, the fractional spread across AOGCMs (the ratio of the standard deviation to the mean) is smaller for OHU (15%) than for AMOC (22%). The small spread of OHU across AOGCMs was noted also by Newsom et al. (2020) and Saenko et al. (2021), and the small sensitivity of OHU to AMOC was shown by Smith et al. (2014) with perturbed versions of a single AOGCM.

### TCR is anticorrelated with piControl AMOC

Given that *H* is proportional to *N* (Sect. 2.5), and seeing that *H* is not correlated with the AMOC (Sect. 2.7), it follows that *N* is not correlated with the AMOC. Hence we infer that the correlation of OHUE ($\kappa =N/T$) with the AMOC must come via surface temperature change *T*, not *N*. That is, OHUE is larger in an AOGCM with a greater piControl AMOC strength, with other things being equal, if a stronger AMOC correlates with a smaller surface warming *T*.

**Fig. 5:**
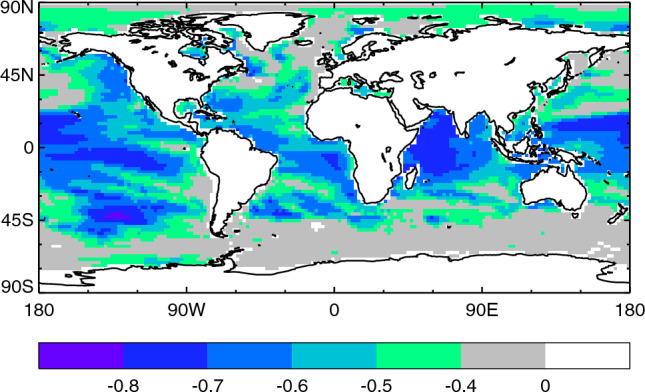
Correlation coefficient across CMIP5&6 AOGCMs of the AMOC strength in piControl with the SST change in the $2\times \text{ CO}_{2}$ state of 1pctCO2

**Fig. 6:**
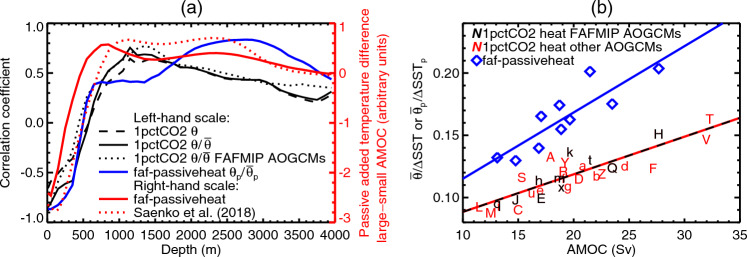
(**a**, left-hand scale, in black and blue) Correlation coefficient as a function of depth across CMIP5&6 AOGCMs of the AMOC strength in piControl with the ocean area-mean $\theta$ and $\theta /\overline{\theta }$ in the $2\times \text{ CO}_{2}$ state of 1pctCO2, and $\theta _{p}/\overline{\theta }_{p}$ in FAFMIP faf-passiveheat, where $\theta$ and $\theta _{p}$ are respectively the ocean temperature change and the passive heat tracer, whose means over the ocean volume are $\overline{\theta }$ and $\overline{\theta }_{p}$. (**a**, right-hand scale, in red) Difference in the passive heat tracer $\theta _{p}$ as a function of depth between the AOGCM variants of Saenko et al. (2018) with strongest and weakest AMOC, and similarly for faf-passiveheat. Note that the zeroes on the right and left are not at the same position on the axes. **b** Relationship across CMIP5&6 AOGCMs of the AMOC strength in piControl with $\overline{\theta }/\Delta \textrm{SST}$ in 1pctCO2 and $\overline{\theta }_{p}/\Delta \text{ SST}_{p}$ in FAFMIP faf-passiveheat. The two latter quantities are proportional respectively to ocean heat uptake efficiency and passive heat uptake efficiency. Letters identify the AOGCMs according to Table 1, CMIP5 with upper-case letters, CMIP6 lower-case

**Fig. 7:**
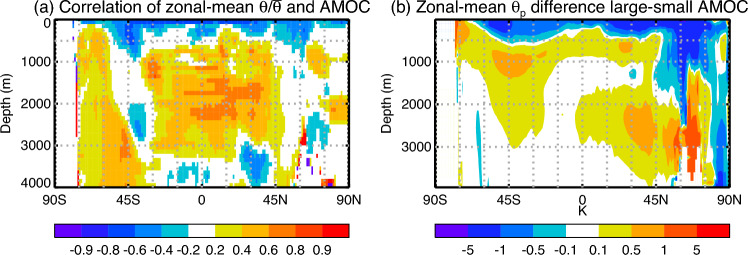
**a** Correlation coefficient across CMIP5&6 AOGCMs of the AMOC strength in piControl with zonal-mean $\theta /\overline{\theta }$ in the $2\times \text{ CO}_{2}$ state of 1pctCO2, where $\theta$ is the ocean temperature change and $\overline{\theta }$ is its mean over the ocean volume. **b** Difference in zonal-mean $\theta _{p}$ in FAFMIP faf-passiveheat between the means of sets of CMIP5&6 AOGCMs with large and small AMOC strength in piControl

Considering the $2\times \text{ CO}_{2}$ state of 1pctCO2, when $T=\text{ TCR }$ by definition, we infer that TCR and AMOC must be anticorrelated. This inference is corroborated by the AOGCM results ($r=-0.65$, Fig. 2e). As far as we know, the anticorrelation of TCR and piControl AMOC in CMIP AOGCMs has not previously been reported. (Winton et al. (2014) found an anticorrelation between AMOC and the ratio of TCR to EffCS in a set of AOGCMs developed at the Geophysical Fluid Dynamics Laboratory.)

Moreover, local sea-surface temperature (SST) change in 1pctCO2 is anticorrelated with the AMOC almost everywhere in the world (the stronger the piControl AMOC, the smaller the local warming, Fig. 5). The anticorrelation is strongest within 45^∘^ S–45^∘^ N, just as strong in the Indian and eastern Pacific Oceans as it is in the Atlantic, and relatively weak in the North Atlantic. These features suggest that the anticorrelation is not due to a causal connection between SST change and AMOC.

### EffCS is anticorrelated with piControl AMOC

One explanation for stronger AMOC being associated with smaller $\Delta$SST relates to the climate feedback parameter $\alpha$, which we find to be larger i.e. effective climate sensitivity ($\text{ EffCS }\propto 1/\alpha$) is smaller in models with stronger AMOC ($r=0.53$, Fig. 2f). We do not know the reason for this correlation, assuming it is not random. As far as we are aware, it has not been noticed before, but substantial effects on $\alpha$ connected with patterns of low-latitude SST change have been shown in other contexts (e.g. Winton et al. 2010; Andrews et al. 2015; Gregory and Andrews 2016; Ceppi and Gregory 2019), and can involve the AMOC (Lin et al. 2019). Whatever the physical explanation, if strong AMOC gives large $\alpha$, and since large $\alpha$ gives small *T*, strong AMOC gives small *T*.

### OHUE is related to the depth of warming

Another explanation for the anticorrelation of AMOC and $\Delta$SST is that a larger *fraction* of the heat added at the ocean surface is removed from the upper ocean and transported to the deeper ocean in AOGCMs with stronger AMOC. This mechanism is the essential idea of “ocean heat uptake efficiency” in the zero-layer model, where larger $\kappa$ gives smaller *T* (Eq. 2). It is also consistent with stronger AMOC being associated with penetration of warming to greater ocean depth. Kostov et al. (2014) quantified this effect by evaluating the depth above which a certain fraction of the OHU was contained.

We demonstrate this penetration mechanism by evaluating the correlation of piControl AMOC with global area-mean ocean temperature warming $\theta$ in 1pctCO2 as a function of depth below the ocean surface (dashed black line in Fig. 6a). Note that at the surface this correlation is the same quantity as for Fig. 2e, since $\Delta \text{ SST }$ is identical with $\theta$ at the surface. Because the ocean volume-mean of $\theta$ (proportional to the OHU) is similar in all cases, smaller $\theta$ at the surface is balanced by larger $\theta$ beneath. This relation explains why the correlation coefficient between $\theta$ and AMOC changes sign at around 500 m depth. It has its negative minimum at the surface ($r=-0.66$), and its positive maximum within 1100–1400 m. At greater depth the correlation declines but it remains significant down to around 3000 m.

The correlation of $\theta$ with the AMOC is affected by the AOGCM spread in OHU, which is small but not zero, and which is not correlated with the AMOC (Sect. 2.7). We remove the OHU spread by dividing $\theta$ in each AOGCM by its ocean volume-mean $\overline{\theta }$. The correlation coefficient with the AMOC of $\theta /\overline{\theta }$ as a function of latitude and depth (Fig. 7a) indicates that the fraction of the OHU that is retained near the surface is smaller at all latitudes in AOGCMs with a stronger piControl AMOC, and the fraction stored below a few 100 m is larger at most latitudes. At the surface, the relationship between $\Delta \text{ SST }/\overline{\theta }$ and AMOC ($r=-0.87$, solid black line in Fig. 6a) is stronger than between $\Delta$SST and AMOC (dashed black line). (This difference in *r* is marginally statistically significant, having probability 0.053, obtained by applying the Fisher transformation to the *r* values, and assuming that the transformed values have a Gaussian distribution.)

We expect the quantity $\Delta \text{ SST }/\overline{\theta }$ to be proportional to *T*/*N*, since $\overline{\theta }\propto \text{ OHU }$, $\text{ OHU }\propto N$ (Sect. 2.5) and $\Delta \text{ SST }\propto T$. Therefore its reciprocal $\overline{\theta }/\Delta \textrm{SST}$ should be proportional to *N*/*T*, which is the OHUE. This prediction is correct: the intercept of regression of $\overline{\theta }/\Delta \textrm{SST}$ against OHUE is consistent with zero, and their correlation coefficient is large ($r=0.81$, not shown), hence ${\overline{\theta }/\Delta \textrm{SST}}\propto \text{ OHUE }$ as expected. The strong correlation between $\overline{\theta }/\Delta \textrm{SST}$ and AMOC ($r=0.87$, letters in Fig. 6b with red and black regression line) is therefore consistent with the strong correlation of OHUE and AMOC (Fig. 2d).

We deduce that the relationship of OHUE and AMOC could be physically explained by some property of the ocean which gives *both* a strong AMOC in the piControl state *and* more efficient transport of heat from the surface into the deeper ocean under increasing CO_2_.

### OHUE is related to passive tracer uptake efficiency

To obtain insight concerning the efficiency of transport of heat from the surface to the deep ocean, we use results from the faf-passiveheat experiment, carried out with ten AOGCMs (indicated in Table 1) which participated in the flux-anomaly-forced model intercomparison project (FAFMIP) (Gregory et al. 2016; Couldrey et al. 2021, 2023). This group is a typical subset of the CMIP5&6 AOGCMs. They show similar relationships to the full set of AOGCMs, between piControl AMOC strength and global-mean $\theta /\overline{\theta }$ as a function of depth (black dotted line in Fig. 6a), and between piControl AMOC strength and $\overline{\theta }/\Delta \textrm{SST}$ (which is proportional to OHUE, as described in the penultimate paragraph of Sect. 2.10, black letters in Fig. 6b).

In the FAFMIP faf-passiveheat experiment, no surface heat flux or any other climate forcing is applied. The climate is therefore the same as in piControl. The experiment is 70 years long, and may be an exact rerun of piControl. The ocean contains a passive tracer, denoted $\theta _{p}$, initialised to zero, whose surface flux is prescribed as a function of location and time of year, the same in all years and all AOGCMs. The surface flux is equal to the CMIP5 ensemble-mean time-mean of the change in surface heat flux at the time of $2\times \text{ CO}_{2}$ in 1pctCO2, and is thus typical in magnitude and pattern of ocean heat uptake in response to CO_2_ in AOGCMs. The passive tracer is described as “passive heat”, and expressed in units of temperature change. It tracks where the “added heat” would go if there were no effect of climate change on ocean transports.

Zonal-mean $\theta _{p}$ is largest in the upper ocean because it enters through the surface, and it penetrates deeply in the regions around 45^∘^ N and 45^∘^ S (colours in Fig. 7b). Hence, we suggest that the main processes of passive heat uptake are the AMOC in the North Atlantic, eddy transport down sloping isoneutral surfaces in the Southern Ocean, and wind-driven subduction in the gyres and in the Southern Ocean to the north of the Antarctic Circumpolar Current (Marshall et al. 2015; Morrison et al. 2016; Bronselaer and Zanna 2020; Clément et al. 2022; Wu and Gregory 2022). In other work, the latitude of maximum heat uptake due to the latter process has been found to depend on the location of the line of zero windstress curl (Stewart and Hogg 2019; Lyu et al. 2020), and it is likely that the same applies also to passive heat uptake.

We divide the FAFMIP AOGCMs into groups with stronger and weaker piControl AMOC ($\gtrless 18.8$ Sv, half of them in each group), and consider the difference between the AOGCM-mean $\theta _{p}$ distributions of the groups. Stronger piControl AMOC is associated with deeper tracer uptake and smaller surface concentration at all latitudes (Fig. 7b). Global-mean $\theta _{p}/\overline{\theta }_{p}$ is smaller above 500 m and larger below in AOGCMs with greater AMOC (Fig. 6a, solid red line), where $\overline{\theta }_{p}$ is the ocean volume-mean of $\theta _{p}$. ($\overline{\theta }_{p}$ is very similar in all AOGCMs, because they have the same prescribed field as its surface source.)

A similar result was obtained by Saenko et al. (2018) for variants of an ocean GCM with different piControl AMOC. The vertical profile of the difference in $\theta _{p}$ between their cases with maximum and minimum AMOC (dotted red line in Fig. 6a) is qualitatively similar to faf-passiveheat (solid red line), in having a pronounced minimum at the surface, crossing zero at around 700 m, and small positive values in the deep ocean. With each variant they also carried out a climate-change experiment. With larger AMOC the warming spread more deeply at all latitudes and OHUE was consequently larger.

As we did for temperature $\theta$ (described in the penultimate paragraph of Sect. 2.10), we calculate the ratio of $\overline{\theta }_{p}$ to $\Delta \text{ SST}_{p}$, the sea surface area-mean of $\theta _{p}$. This quantity $\overline{\theta }_{p}/\Delta \text{ SST}_{p}$ is analogous to $\overline{\theta }/\Delta \textrm{SST}$ for heat. It measures the efficiency of processes that remove the added passive tracer from the surface into the deep ocean. We call this “passive heat uptake efficiency”, by analogy with ocean heat uptake efficiency. Like $\overline{\theta }/\Delta \textrm{SST}$, $\overline{\theta }_{p}/\Delta \text{ SST}_{p}$ is strongly correlated with piControl AMOC ($r=0.87$, blue line at $z=0$ in Fig. 6a, blue diamonds in Fig. 6b). Furthermore, $\overline{\theta }/\Delta \textrm{SST}$ and $\overline{\theta }_{p}/\Delta \text{ SST}_{p}$ are strongly correlated ($r=0.85$), but $\overline{\theta }_{p}/\Delta \text{ SST}_{p}$ is larger (blue diamonds lie above black letters in Fig. 6b). From experiments with two OGCMs, Romanou et al. (2017) also found passive tracer uptake efficiency to be greater than OHUE, but by a much larger ratio (a factor of five) than we find in FAFMIP AOGCMs.

Further analysis shows that $\overline{\theta }_{p}/\Delta \text{ SST}_{p}$ is larger than $\overline{\theta }/\Delta \textrm{SST}$ because of the weakening of the AMOC, which occurs in 1pctCO2 but not faf-passiveheat (see Appendix D). However, the relationship between $\overline{\theta }_{p}/\Delta \text{ SST}_{p}$ and piControl AMOC cannot be due to the weakening of the AMOC or to any other climate-change effect on heat uptake processes, because there is no climate change in faf-passiveheat. OHUE anticorrelates significantly with the change of the AMOC in 1pctCO2 (Winton et al. 2014, their Figure 4), but this could arise because the weakening of the AMOC is correlated with its piControl strength ($r=-0.79$ in CMIP5&6 excluding three outliers), as found in successive generations of AOGCMs (see Appendix D for discussion).

Since it cannot be due to climate change, the relationship between the AMOC and passive tracer uptake efficiency must arise from some property of the piControl state. Given also the similarity of $\overline{\theta }/\Delta \textrm{SST}$ and $\overline{\theta }_{p}/\Delta \text{ SST}_{p}$, we can take a step beyond the conclusion of Sect. 2.10, in deducing that the relationship between OHUE and AMOC could be explained by a property of the piControl state which gives both a strong AMOC and efficient *passive* tracer uptake. Later (Sect. 3.3.2) we suggest how this might happen.

### Summary of analysis of the transient $2\times \text{ CO}_{2}$ state

In this section, we summarise our findings so far. During the analysis, we used global-mean sea-surface temperature change $\Delta$SST at the time of $2\times \text{ CO}_{2}$ in 1pctCO2 as a substitute for the transient climate response (TCR, defined as global-mean surface air temperature change *T* at that time) because *T* and $\Delta$SST are highly correlated (as explained at the start of Sect. 2 and in Appendix A.2). Here for simplicity and clarity we mention TCR only, not $\Delta$SST or *T*. Note that when we mention “correlation” in this summary, it refers to the relationship between two quantities across our set of CMIP5&6 AOGCMs. The summary is as follows:

1. We have confirmed the finding by Kostov et al. (2014) of a **strong correlation between the strength of the Atlantic meridional overturning circulation (AMOC) in piControl experiments and ocean heat uptake efficiency (OHUE)** in W m^-2^ K^-1^.
2. However, the **ocean heat uptake (OHU) is not significantly correlated with piControl AMOC, nor with OHUE**, where OHU in ZJ is the integral ${\mathcal {A}}\int N\,\textrm{d}t$ up to the time of $2\times \text{ CO}_{2}$, and *N* is the global-mean rate of ocean heat uptake in W m^-2^.
3. **TCR is anticorrelated with piControl AMOC**.
4. Across AOGCMs, **OHU is proportional to the rate of OHU** $\int _0^t N(t')\,\textrm{d}t' \propto N(t)$, which means they all have a similar time-profile of *N*(*t*).
5. **AOGCMs with larger TCR have greater OHU**, but
6. **about half of the AOGCM-mean OHU is unrelated to TCR**.
7. **OHUE is anticorrelated with effective climate sensitivity (EffCS)**.
8. **EffCS is anticorrelated with piControl AMOC**.
9. **In an AOGCM with stronger piControl AMOC, heat is removed more efficiently from the upper ocean** (the top few 100 m) and penetrates more deeply. This relationship arises from the AOGCM spread in some property of the piControl state affecting both the AMOC strength and passive tracer uptake processes.

## A new conceptual model of global ocean heat uptake

**Fig. 8:**
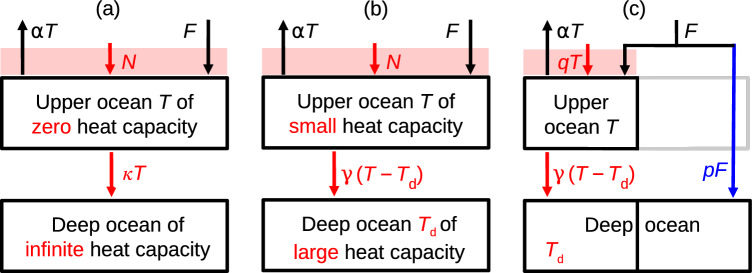
Models of global ocean heat uptake. **a** Zero-layer model (Sect. 1.2), **b** two-layer model (Sect. 3.1.2), **c** *MT*2 model (Sect. 3.1)

In this section, we present a new conceptual model, called “*MT*2”, for OHU in AOGCM experiments forced by CO_2_ increase. The formulation of the model was guided by the empirical results that we have described (Sect. 2.12). The choices made in its construction and the evaluation of its coefficients and consequences involve some rather detailed analysis and arguments, which are given in Appendix C. In this section we set out the formulation of the *MT*2 model without describing its derivation (Sect. 3.1), use it to reexpress the global energy balance (Sect. 3.2), offer a physical interpretation for it (Sect. 3.3), and assess its accuracy in reproducing AOGCM results (Sect. 3.4). Wherever relevant, we explain how the *MT*2 model incorporates or accounts for the behaviour of the CMIP5&6 AOGCMs as summarised in Sect. 2.12.

For reference, Fig. 8 compares diagrams of the *MT*2, zero- and two-layer models, and Appendix E tabulates their equations. We classify quantities in these equations as “AOGCM-neutral” or “AOGCM-specific”. For each AOGCM, the model uses a *different* value of a given AOGCM-specific quantity. For every AOGCM, the model uses the *same* value of a given AOGCM-neutral quantity. The values of the AOGCM-neutral quantities are chosen to fit CMIP5&6 results; for a different set of AOGCMs the optimal choices would be different.

### Formulation of the $MT2$ model

#### Rate of ocean heat uptake in the $MT2$ model

The *MT*2 model has two routes for heat from the surface to the deep ocean, with fluxes $N_{M}$ and $N_{T}$, which depend on the piControl AMOC strength *M* and the global-mean surface air temperature change *T* respectively (Fig. 8c). According to the *MT*2 model, the rate of OHU *N*(*t*) simulated by a given AOGCM in abrupt4xCO2 and 1pctCO2 is

5 $$\begin{aligned} \begin{array}{c} N(t) = N_{MT}(t) = N_{M}(t) + N_{T}(t) \\ \text{ with } \quad N_{M}(t)=pF(t) \quad \text{ where }\quad p=s_{0}\,(M-M_{0})\\ \text{ and }\quad N_{T}(t)=q(t)T(t). \end{array} \end{aligned}$$

The parameters $M_{0}=-10.2$ Sv and $s_{0}=0.0047\pm 0.0001$ Sv^-1^ are AOGCM-neutral constants of the *MT*2 model. Since *p* depends on the AMOC strength *M* in piControl, it is an AOGCM-specific constant. The CMIP5&6 AOGCM-mean $\langle p \rangle =14$% and the standard deviation across AOGCMs is 3%. (Here and subsequently, $\langle  \rangle$ denotes the mean over AOGCMs.) The coefficient *q*(*t*) is an AOGCM-neutral but scenario-dependent function of time.

Since $N_{T}$ is a function of *T*(*t*), it is AOGCM-specific, as well as time- and scenario-dependent. $N_{M}$ is AOGCM-specific through *M*, and time- and scenario-dependent through *F*(*t*), but does not depend on *T*(*t*).

To evaluate quantities with the *MT*2 model, we must adopt a value for the forcing due to CO_2_. Evaluated from years 1–20 of abrupt4xCO2 by the method of Gregory et al. (2004), our set of CMIP5&6 AOGCMs have $F_{4\times }=7.6\pm 1.0$ W m^-2^. The spread of about 10% comes from tropospheric adjustment, and is small compared with the AOGCM spread in $\alpha$ and *p*. We assume an AOGCM-neutral ERF for $\text{4 }\times \text{ CO}_{2}$ of $F_{4\times }=7.5$ W m^-2^, which is twice the stratosphere-adjusted $F_{2\times }=3.75$ W m^-2^ of Forster et al. (2021).

#### Two-layer model as a component of the $MT2$ model

The *MT*2 model calculates $N_{T}(t)$ from *T*(*t*) using the two-layer model (Fig. 8b). Coupled to the global energy balance (Eq. 1), the two-layer model has been used to make projections of *T* for a range of scenarios and timescales (Gregory 2000; Held et al. 2010; Geoffroy et al. 2013b; Gregory et al. 2015). In those applications, the two-layer model simulates the whole of *N*, whereas as a component of the *MT*2 model it simulates only the part $N_{T}=N-N_{M}$.

According to the two-layer model,

6 $$\begin{aligned} N_{T} = c_{u} \frac{\textrm{d}T}{\textrm{d}t} + \Phi \qquad \text{ and } \qquad \Phi = \gamma (T-T_{d}) = c_{d} \frac{\textrm{d}T_{d}}{\textrm{d}t}. \end{aligned}$$

In Eq. (6), $T_{d}$ is the temperature change of the deep ocean layer relative to the unperturbed state, $c_{u}$ and $c_{d}$ are the heat capacities for the upper and deeper ocean layers per unit of global area ($\text{ J }~\text{ m}^{-2}~\text{ K}^{-1}$), and $\Phi$ is the heat flux from the upper ocean layer to the deep layer, where $\gamma$ (W m^-2^ K^-1^) is assumed to be constant in time.

$N_{T}(t)$ is obtained from the solution of Eq. (6), given $T(t')$ for $t'<t$ as a boundary condition and $T_{d}(0)=0$. In Eq. (5) we write this solution as $N_{T}=qT$ by *defining* $q(t)\equiv N_{T}(t)/T(t)$, where *q*(*t*) is an AOGCM-neutral scenario-dependent coefficient. In this form, $N_{T}(t)$ can be calculated simply from *T*(*t*) alone, without knowledge of $T(t')$ for earlier $t'<t$. It is not obvious *a priori* that the formula *q*(*t*)*T*(*t*) will agree with the solution for $N_{T}(t)$ from Eq. (6) given *T*(*t*) for any individual AOGCM. It works because, under a given scenario, all AOGCMs have a similar time-profile of *T*, as discussed further in Appendix C.4.4. The accuracy of the formula $N_{T}=qT$ is evaluated in Sects. 3.4 and 4.1.

The *MT*2 two-layer model has heat capacities of ${\mathcal {A}}c_{u}=60\pm 2$ ZJ K^-1^ and ${\mathcal {A}}c_{d}=454\pm 14$ ZJ K^-1^ (corresponding to global-mean water thicknesses of about 26 m and 200 m), and thermal coupling coefficient $\gamma =0.470\pm 0.008$ W m^-2^ K^-1^ between the layers. These values are chosen so that for abrupt4xCO2 the two-layer model yields the AOGCM-mean $\langle N_{T}(t) \rangle$ when given $\langle T(t) \rangle$ as input, whence $q(t)=\langle N_{T}(t) \rangle /\langle T(t) \rangle$. (See Appendix C.4.2 for the method used to obtain the values of the parameters, and comparison with Geoffroy et al. (2013b).

#### Time-integral ocean heat uptake in the $MT2$ model

OHU *H*(*t*) in the *MT*2 model is given by

7 $$\begin{aligned} \begin{array}{c} H(t) = H_{MT}(t) \equiv H_{M}(t) + H_{T}(t) \\ \displaystyle \text{ with } \qquad H_{M}=\int _{0}^{t}\,N_{M}(t')\,\textrm{d}t' =U_{0}+p\int _{0}^{t}\,F(t')\,\textrm{d}t' \\ \displaystyle \text{ and } \qquad H_{T}=\int _{0}^{t}\,N_{T}(t')\,\textrm{d}t'=\int _{0}^{t}\,q(t')T(t')\,\textrm{d}t', \end{array} \end{aligned}$$

where the AOGCM-specific constant *p* is defined by Eq. (5). The AOGCM-neutral constant $U_{0}=84$ ZJ is a relatively small contribution to *H*, included for accuracy. (We do not show $\textrm{d}U_{0}/\textrm{d}t$ in Eq. (5), but we include it in our calculations.)

Since *q* is AOGCM-neutral, it is obvious that $H_{T}$ is larger in AOGCMs with larger *T*. By contrast, $H_{M}$ is independent of *T*. Therefore *H* is larger in AOGCMs with larger *T* (point **5** of Sect. 2.12). We see later (Sect. 3.4) that $\langle H_{M} \rangle$ and $\langle H_{T} \rangle$ are of comparable size, meaning that about half of $\langle H \rangle$ is unrelated to *T* (point **6**). It is not obvious or necessarily true that $H\propto N$ across AOGCMs at any *t* in the *MT*2 model (point **4**), but it holds in 1pctCO2 and abrupt4xCO2 (Appendix C.5).

#### Coefficients of the $MT2$ model and its $MT2T$ variant

The *MT*2 model has six time-independent, scenario-independent and AOGCM-neutral parameters: $M_{0},s_{0},U_{0}$ for $N_{M}$, $c_{u},c_{d},\gamma$ for $N_{T}$. In Sect. 3.4 we show that we can make the *MT*2 model more accurate, while less parsimonious, if we calibrate the two-layer $c_{u},c_{d},\gamma$ individually for each AOGCM, rather than for all together using the AOGCM mean. This variant of the model is called “*MT*2*T*”. *MT*2*T* has the same formulation as *MT*2 (Eq. 5), and uses the same $M_{0},s_{0},U_{0}$ for $N_{M}$; $c_{u},c_{d},\gamma$ are AOGCM-specific, but still time-independent, and *q*(*t*) is AOGCM-specific and time-dependent.

### Energy balance of the $MT2$ model

In the *MT*2 model, the energy balance is

8 $$\begin{aligned} F - \alpha T = N = N_{M} + N_{T} = p F + q T \end{aligned}$$

(from Eqs. 1 and 5). The proportion $p=N_{M}/F=s_{0}(M-M_{0})$ of the forcing *F* is absorbed by the ocean without raising *T*. Rearranging Eq. (8), we obtain

9 $$\begin{aligned} F-pF = \alpha T + qT \Rightarrow T = \frac{(1-p)F}{\alpha +q}. \end{aligned}$$

Considering Eq. (9), we can see that *T* is smaller for a given *F* in AOGCMs with larger *M* for two reasons: $\alpha$ is larger (EffCS is smaller, point **8** of Sect. 2.12), and *p* is larger (*F* is removed from the surface more effectively, point **9**). Applied to the $2\times \text{ CO}_{2}$ state of 1pctCO2, these reasons jointly account for the anticorrelation of AMOC *M* and TCR *T* (point **3**).

### Physical interpretation of the $MT2$ model

In this section we offer some physical interpretations for the *MT*2 model as a means to connect terms in the model with physical processes, although only in a speculative manner. In concluding the paper (Sect. 6.3), we remark on questions raised by this interpretation that require further analysis of AOGCMs.

**Fig. 9:**
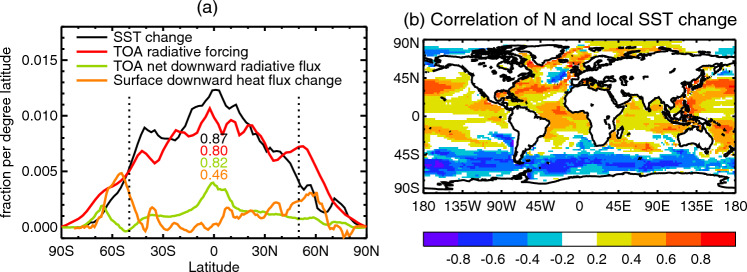
The geographical pattern of SST change and energy fluxes for the $2\times \text{ CO}_{2}$ state of 1pctCO2 in a set of CMIP5 AOGCMs. **a** AOGCM-mean meridional distribution of SST change, surface and top-of-atmosphere energy fluxes. The numbers in the centre are the fraction of the global area-integral of each quantity which occurs within 50^∘^ N–50^∘^  S. The ocean within this latitude range is 80% of the global ocean area and occupies 57% of the global area (land and ocean); 78% of global area is within this latitude range. **b** Correlation across AOGCMs between global-mean *N* and local SST change

#### Temperature as a passive tracer

The term $N_{M}$ of the *MT*2 model consists of a part of OHU which depends on the piControl AMOC strength *M* and is thus specific to each AOGCM, and a part which is the same in all AOGCMs (the $M_{0}$ term). The partitioning of $N_{M}$ into these two parts is arbitrary to the extent that we cannot distinguish between OHU that is related to AOGCM-mean AMOC and OHU that is unrelated to AMOC but the same in all AOGCMs.

We have not investigated the AOGCM-neutral part of OHU. This part could be due to OHU by large-scale wind-driven subduction, which is an important and widespread process in the Southern Ocean (Liu and Huang 2011; Williams and Meijers 2019). The wind-driven overturning circulation resolved in GCMs has a strength of 10 s of Sv (the same order of magnitude as *M* and $M_{0}$). Its contribution to OHU depends on windstress, which is not correlated with the AMOC, and may be more similar among AOGCMs than eddy-induced advection (Sect. 3.3.2).

For a physical interpretation of the *M*-dependent part of OHU, we recall that a passive tracer (i.e. one which has no effect on ocean state or dynamics), applied at the sea surface with the same geographical distribution as heat uptake under CO_2_, is removed more efficiently from the surface and taken deeper into the ocean of AOGCMs with larger *M* (Sect. 2.11). This is a property of the unperturbed ocean state, not due to any aspect of climate change.

The majority of heat uptake occurs in the Southern Ocean, where the added heat is an almost passive tracer (Winton et al. 2013; Gregory et al. 2016; Couldrey et al. 2021). Thus, if heat is mostly taken up like a passive tracer, we expect greater OHU for a given *T* in AOGCMs with stronger *M*; equivalently, for a given OHU, *T* is smaller with stronger *M*, so OHUE is larger.

Heat added to the ocean during climate change causes the AMOC to weaken, and this reduces the efficiency of heat and passive tracer uptake. However, the weakening mostly affects just the North Atlantic, which is a relatively small region, whereas all latitudes are involved in the phenomenon that correlates greater global tracer uptake efficiency with piControl AMOC (see also Appendix D).

#### A role for ocean stratification and mesoscale eddies

We propose that the relationship of AMOC and OHUE expressed by $N_{M}$ is explained by a common influence on both. The common factor could be the vertical stratification of the global ocean. We expect greater stratification to inhibit both heat uptake (Newsom et al. 2023) and sinking, thus decreasing OHUE and AMOC respectively.

The AOGCM spread in stratification could in turn arise from the effect of parametrised mesoscale eddies on the state of the ocean (Marshall and Zanna 2014). An eddy influence is suggested by the fairly strong anticorrelation ($r=-0.76$) between OHUE and $K_{GM}$, the diffusivity parameter in the mesoscale eddy-induced advection scheme, across a set of AOGCMs from the Coupled Model Intercomparison Project Phase 3 where $K_{GM}$ was a global constant (Kuhlbrodt and Gregory 2012). That is, models with stronger eddy-induced advection have smaller OHUE.

In the majority of recent AOGCMs, such as those used in CMIP5&6, $K_{GM}$ is a spatio-temporally varying function of the flow field. Even so, we suggest that the correlation found by Kuhlbrodt and Gregory (2012) between weak AMOC and strong $K_{GM}$ is due to the flattening of neutral directions by eddy-induced advection, with this effect also active in models with flow-dependent $K_{GM}$. Flattening of neutral directions in turn increases the vertical stratification, thus inhibiting convection and sinking, whereas weak $K_{GM}$ supports enhanced ventilation of the deep ocean. We cannot test this connection with CMIP5&6 AOGCMs because diagnostics of $K_{GM}$ are not generally available, but Saenko et al. (2018) showed such a connection in a set of ocean steady states produced with different choices of a factor multiplying the spatially varying $K_{GM}$ in their GCM. Smaller $K_{GM}$ gave stronger AMOC and greater passive heat uptake efficiency.

A critical feature of $N_{M}$ is that it stores a proportion of *F* in the ocean without any effect on either the global-mean surface *T* or the deep-ocean temperature in the two-layer component of the *MT*2 model. We suggest that this type of heat storage could happen if the processes that $N_{M}$ represents occur at high latitude.

At high latitude, vertical ocean transport is more efficient because the stratification is weaker, meaning the neutral surfaces are tilted further from the horizontal. Advection and diffusion occur predominantly along these surfaces (*cf.* Church et al. 1991; Saenko et al. 2021), and hence may convey added heat downwards from the surface more rapidly with a given degree of surface warming than at low latitude, where the stronger stratification inhibits vertical transport. Furthermore, climate feedback is weak ($\alpha$ is small, effective climate sensitivity large) for high-latitude warming (Armour et al. 2013; Rose et al. 2014), because atmospheric stability confines its effects to the near-surface, where it causes low-level amplifying shortwave cloud feedbacks (Ceppi and Gregory 2019; Salvi et al. 2022). Because climate feedback is weak, high-latitude *F* and *N* are similar in size (especially in the Southern Ocean, red and orange lines in Fig. 9a).

Putting these together supports the idea of surface *F* being taken up passively and advectively at high latitude with only a small effect on local SST and *T*. To account for the AOGCM behaviour represented by the *MT*2 model, we hypothesise

- that the downwelling heat flux $N_{M}$ is stored in a part of the deep ocean which is separate from the deep layer of the *MT*2 two-layer component, and hence does not affect $T_{d}$.
- that the heat capacity of this part is very large, or the timescale for recirculation of deep water to the upper ocean is much longer than the centennial timescale of our analysis, so that the accumulation of heat by $N_{M}$ in the deep ocean does not affect the ongoing rate of uptake.

#### The role of low latitudes

Global *T* is mostly due to low-latitude SST change; the fraction of the global area-integral of SST change contributed by the area poleward of 50^∘^ is only about 15% in 1pctCO2 (black line in Fig. 9a), because SST change is smaller there, and it is the minority of the area *viz.* 20% of the global ocean area, although its fraction of global surface *N* is about 50% (orange line in Fig. 9a).

The majority of *F* (80%) falls equatorwards of 50^∘^, but most of it is opposed by climate feedback $\alpha T$ (Eq. 1) i.e. reradiated to space. Therefore $N\ll F$ at the top of the atmosphere at low latitude (green line compared with red line in Fig. 9a), and about half of the low-latitude top-of-atmosphere *N* is exported polewards across 50^∘^ by the atmosphere (accounting for the difference between the green and orange lines).

It is natural to suppose that $N_{T}$ comes from low latitude, because that complements $N_{M}$, and because $N_{T}$ is caused by *T* change. It involves mostly dianeutral processes that depend on temperature gradients, represented by the term $\gamma (T-T_{d})$, which are most likely to be relevant where SST change is largest. We support this hypothesis with two lines of evidence.

First, since low-latitude SST change dominates global *T* and $N_{T}\propto T$, we expect $N_{T}$ to be large when low-latitude warming is large. This relationship is corroborated by the positive correlation across AOGCMs in the $2\times \text{ CO}_{2}$ state of 1pctCO2 between global-mean *N* and local SST change within 50^∘^  N–50^∘^  S (Fig. 9b, except in small regions of equatorward currents and upwelling on the east of gyres). South of 50^∘^  S, the correlation is negative i.e. local SST change is small in the Southern Ocean when *N* is large. We suggest that in this region *N* causes SST change, rather than vice-versa; large $N_{M}$ suppresses Southern Ocean SST change by removing the added heat efficiently from the surface (Newsom et al. 2020). The heat thus accumulated below the surface is represented by the deep ocean on the right-hand side of Fig. 8c; in reality some remains at high latitude, and some is conveyed equatorward at depth, mostly along neutral directions.

Second, the heat capacities of the *MT*2 two-layer component are small. The upper-ocean $c_{u}$ is equivalent to about 50 m thickness of water over the ocean area equatorward of 50^∘^, consistent with the low-latitude mixed layer. The deep-ocean $c_{d}$ is equivalent to about 350 m of water over low-latitude ocean, consistent with OHU being confined to the upper few hundred metres at low latitude in the AOGCMs. If $\gamma (T-T_{d})$ is equated to a diffusive heat flux $cD(T-T_{d})/Z$ between two layers separated by distance $Z=350$ m, the diapycnal diffusivity $D=Z\gamma /c=4\times 10^{-5}$ m^2^ s^-1^, which is the expected order of magnitude. This indicates that the “deep” layer of low-latitude heat uptake is actually quite shallow (Fig. 7); it does not include the ocean below the thermocline, nor the high-latitude deep ocean.

Note, however, that the two-layer model is a simplification; $c_{d}$ is only an empirical parameter, and not literally the heat capacity of a well-mixed layer. We find below (Sect. 3.4) that the *MT*2*T* model reproduces AOGCM OHU more accurately than *MT*2, mostly because of its AOGCM-specific $c_{d}$. It could be that model spread in $c_{d}$ reflects the spread in stratification among AOGCMs at low latitudes.

Because $c_{d}$ is fairly small, $T_{d}$ warms up substantially over decades, causing $q=N_{T}/T$ and hence OHUE to decrease. Nevertheless, the accuracy of the *MT*2 model with time-constant parameters throughout both scenarios indicates that climate change does not much weaken the processes themselves which $N_{T}$ represents. For instance, the increase in low-latitude stratification could inhibit OHU, but does not require $\gamma$ to decrease in our analysis.

### Evaluation of the $MT2$ model of ocean heat uptake

**Fig. 10:**
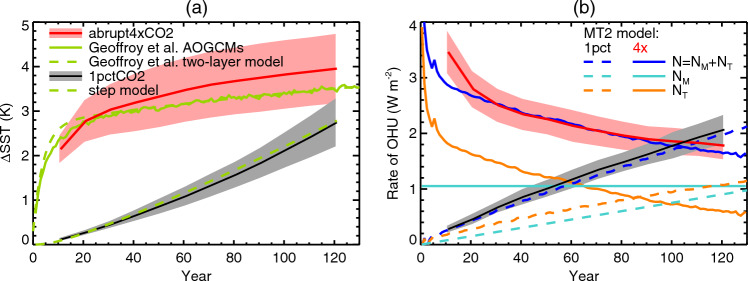
AOGCM-mean global-mean SST change **a** $\Delta$SST and **b** rate of OHU *N* as a function of time (plotted at the mid-year of overlapping 20-year means at 10-year intervals) in CMIP5&6 1pctCO2 and abrupt4xCO2 experiments. The shaded envelopes show $\pm 1$ standard deviation of the AOGCM ensemble. **a** $\Delta$SST is compared with the two-layer model (Geoffroy et al. (2013b), Appendix C.4.2), and the step model (Appendix C.6). Global-mean surface air temperature change $T=1.5\,{\Delta \textrm{SST}}$ (to an excellent approximation). **b** *N* is compared with the results of the *MT*2 model given AOGCM-mean *T*. The *MT*2 timeseries have been smoothed with a running 10-year mean to reduce the effect of interannual variability in *T*

**Fig. 11:**
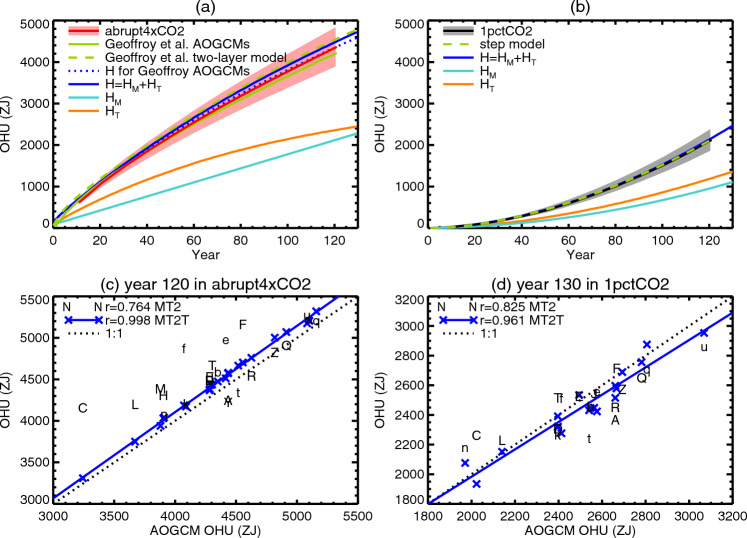
**a**, **b** AOGCM-mean OHU as a function of time (plotted at the mid-year of overlapping 20-year means at 10-year intervals) in CMIP5&6 abrupt4xCO2 and 1pctCO2 experiments respectively, compared with the two-layer model (Geoffroy et al. (2013b), Appendix C.4.2), the step model (Appendix C.6) and the *MT*2 model. The shaded envelopes show $\pm 1$ standard deviation of the AOGCM ensemble. **c**, **d** Comparison of OHU (20-year means, centred on the stated year) from AOGCMs in abrupt4xCO2 and 1pctCO2 respectively with the *MT*2 and *MT*2*T* models. Letters identify AOGCMs according to Table 1, CMIP5 with upper-case letters, CMIP6 lower-case. Solid lines show regression of *MT*2*T* OHU against AOGCM OHU

**Fig. 12:**
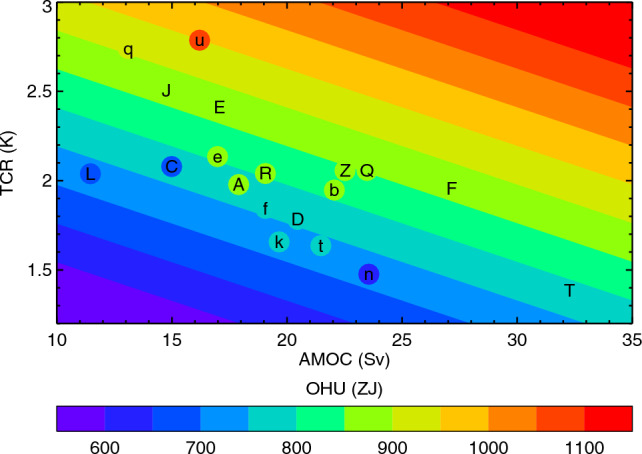
OHU for the $2\times \text{ CO}_{2}$ state of 1pctCO2 as a function of piControl AMOC *M* and TCR, with circles for CMIP5&6 AOGCMs and contours for the *MT*2 model $H_{MT}(M,T)$ (Eq. 7). The letters identify the AOGCMs according to Table 1, CMIP5 with upper-case letters, CMIP6 lower-case

In this section, we assess the performance of the *MT*2 model in estimating the rate *N*(*t*) of OHU and the accumulated OHU *H*(*t*), given *T*(*t*) from CMIP5&6 AOGCMs in 1pctCO2 and abrupt4xCO2 experiments. Note that the *MT*2 model parameters are derived from AOGCM-mean timeseries in abrupt4xCO2 (Appendix C), but the evaluation considers its reproduction of individual AOGCMs as well as the mean, in both scenarios.

In 1pctCO2, in which $F\propto t$, the zero-layer model (Eq. 2) gives $N\propto T\propto t$. There is a small acceleration in AOGCM-mean $\langle T \rangle$ (black line in Fig. 10a) and in *MT*2 $\langle N_{T} \rangle$ (Fig. 10b, dashed orange line), but it does not deviate strongly from a linear increase in time. Since $N_{M}\propto F$ (Eq. 5) it too increases linearly in time (dashed turquoise line), and hence so does $\langle N \rangle =\langle N_{M} \rangle +\langle N_{T} \rangle$ (dashed blue line). Therefore $\langle H_{M} \rangle$, $\langle H_{T} \rangle$ and *MT*2 $\langle H \rangle$ all rise approximately quadratically in time (Eq. 7, Fig. 11b, turquoise, orange and blue lines).

In abrupt4xCO2, in which $F=F_{4\times }$ is constant, $\langle T \rangle$ rises rapidly for the first couple of decades (red line in Fig. 10a), while the upper ocean is warming rapidly, and slowly thereafter. Like *F*, $\langle N_{M} \rangle$ is constant in time, but $\langle N_{T} \rangle$ is always declining, more quickly for the first couple of decades (Fig. 10b, solid turquoise and orange lines). Consequently $\langle H_{M} \rangle$ increases linearly in time, while $\langle H_{T} \rangle$ increases initially rapidly, then at a slowly declining rate (Fig. 11a).

For both scenarios, *MT*2 $\langle N_{MT} \rangle$ is an excellent fit to AOGCM $\langle N \rangle$ except for the first 20 years of abrupt4xCO2 (dashed blue is close to black, solid blue close to red, in Fig. 10b), and *MT*2 $\langle H_{MT} \rangle$ for AOGCM $\langle H \rangle$ (blue and red lines are very close in Fig. 11a, blue and black lines in Fig. 11b). In both scenarios, $\langle H_{M} \rangle$ and $\langle H_{T} \rangle$ are of similar size.

The good agreement of *MT*2 with the AOGCMs for *N*(*T*) implies that $\text{ OHUE }=N/T$ must also be reproduced well. In 1pctCO2, OHUE declines slowly because *N* does not accelerate as much as *T*; in abrupt4xCO2, OHUE declines quickly because *N* is decreasing while *T* is increasing. OHUE is the subject of Sect. 4.

Estimating $H_{MT}$ in abrupt4xCO2 for each AOGCM individually from its own *T*(*t*) and *M*, we find that the correlation coefficient between $H_{MT}$ and $H_{T}$ across AOGCMs is about 0.8 at all times (solid red in Fig. 18a) and the RMS of the error $H_{MT}(t)-H(t)$ is always less than 10% of the AOGCM-mean *H* after year 20 (solid red in Fig. 18b). For example, in the time-mean of years 110–130, the correlation is 0.76 (letters in Fig. 11c), and the RMS error is 380 ZJ, 9% of the AOGCM-mean *H*. The *MT*2 model overestimates *H* in AOGCMs where it is small and underestimates where it is large, so the coefficient of variation is smaller for $H_{MT}$ than *H* (8.3% and 10.8% respectively). This deficiency indicates that the *MT*2 model cannot sufficiently represent the diversity of AOGCM heat uptake processes.

The *MT*2*T* variant of *MT*2, with AOGCM-specific *q*(*t*) (Sect. 3.1.4), matches *H*(*t*) in abrupt4xCO2 for every AOGCM excellently (crosses in Fig. 11c, dotted red lines in Fig. 18a, b). For the time-mean of years 110–130, the RMS error is reduced by a factor of three to 3% of the AOGCM mean, and the regression slope of $H_{MT}$ against *H* is $1.04\pm 0.01$ (blue line in Fig. 11c). Most of this improvement comes from $c_{d}$, which has a correlation coefficient of 0.46 (significant at 5%) with *H*, whereas $c_{u}$ and $\gamma$ have insignificant correlations with *H*. Although it has more AOGCM-specific parameters than *MT*2, the accuracy of *MT*2*T* is a non-trivial result. It means that AOGCM *N*(*t*) is reproduced by $pF+N_{T}(t)$, where *F* is AOGCM-neutral, constant *p* depends only on the AOGCM-specific *M*, and $N_{T}(t)$ is calculated by the two-layer model given AOGCM-specific *T*(*t*).

The circles in Fig. 12 show OHU of individual AOGCMs in the $2\times \text{ CO}_{2}$ state of 1pctCO2 (20-year mean centred on year 70) plotted against their *M* and *T* (TCR); and the underlying field is *MT*2 OHU ($H_{MT}$ of Eq. 7). Comparing the colours of the circles and the background gives an indication of the accuracy of the *MT* model. As for abrupt4xCO2, the *MT*2 model generally overestimates small AOGCM OHU and underestimates large OHU, but there is scatter in both directions.

In 1pctCO2 the correlation between *MT*2 $H_{MT}$ and AOGCM *H* increases and the RMS error decreases over time as the signal grows (black lines in Fig. 18a,b), both being better than in abrupt4xCO2 after year 50. The correlation coefficients of AOGCM *H* for the time-mean of years 121–140 (Fig. 11d) with $H_{MT}$ computed for individual AOGCMs by *MT*2 and *MT*2*T* are 0.83 and 0.96 respectively, RMS errors 6% and 4% of the AOGCM mean, and the regression slope of *MT*2*T* $H_{MT}$ against AOGCM *H* is $0.92\pm 0.07$. We recall that the calibration is done for abrupt4xCO2. It is accurate for 1pctCO2 too because of the linear behaviour of the system (Good et al. 2011; Gregory et al. 2015) (see Appendix C.4.2 for further discussion).

### Lack of correlation of OHU with the AMOC

**Fig. 13:**
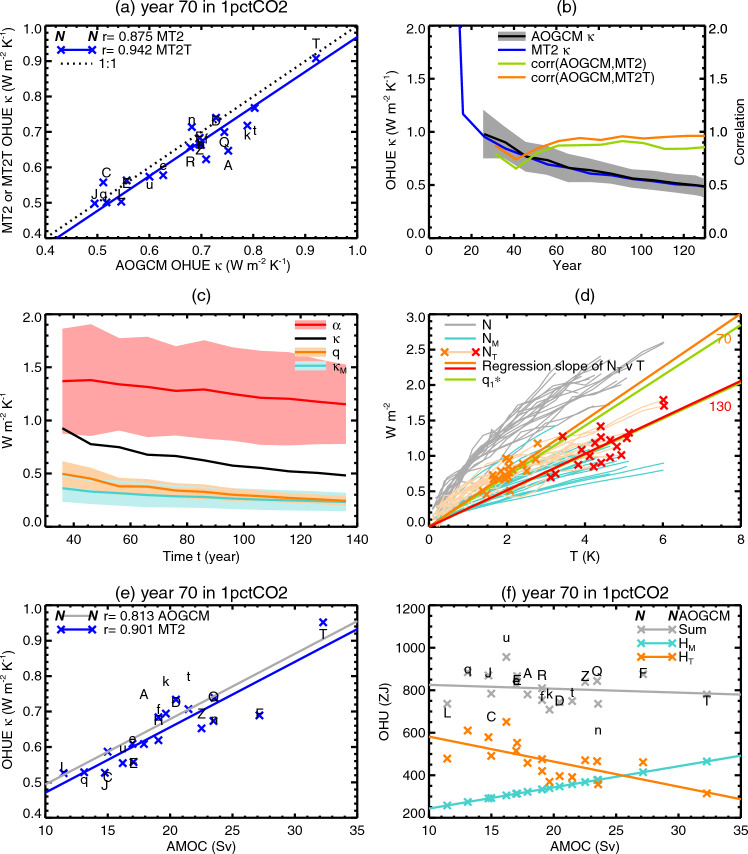
Predictions of the *MT*2 and *MT*2*T* models compared with results diagnosed from CMIP5&6 AOGCMs for OHUE and OHU in 1pctCO2 experiments. **a** Comparison of OHUE $\kappa$ from AOGCMs with the *MT*2 and *MT*2*T* models in the $2\times \text{ CO}_{2}$ state, letters for AOGCMs, solid line showing regression of *MT*2*T* $\kappa$ against AOGCM $\kappa$. (**b**, left-hand axis) AOGCM-mean OHUE as a function of time, with $\pm 1$ standard deviation of the AOGCM ensemble shown as a shaded envelope, compared with the *MT*2 ensemble mean, (**b**, right-hand axis) Correlation coefficient across the ensemble as a function of time of AOGCM $\kappa$ with *MT*2 and *MT*2*T* $\kappa$. **c** Climate feedback parameter $\alpha$ and OHUE from AOGCMs as a function of time, and components of OHUE from the *MT*2*T* model (Eq. 10), with $\pm 1$ standard deviation of the ensemble shown as shaded envelopes. **d** *N* and its components $N_{M},N_{T}$ in the *MT*2 model (Eq. 5) for individual AOGCMs as a function of *T*. Thin lines join successive 20-year means at 10-year intervals, orange and red crosses for 20-year means centred on years 70 and 130, thick lines in the same colour are regressions against *T*, and the green line is from an approximate formula for the slopes (Eq. C53). **e** Relationship between OHUE $\kappa$ in the $2\times \text{ CO}_{2}$ state and AMOC strength in piControl, letters for AOGCMs, symbols for the *MT*2 model, lines for regression of OHUE against AMOC. **f** Relationship between OHU in the $2\times \text{ CO}_{2}$ state and AMOC strength in piControl, letters for AOGCMs, grey symbols for the *MT*2 model, with the terms of *MT*2 in other colours (Eq. 7). Letters in **a**, **e**, **f** identify the AOGCMs according to Table 1, CMIP5 with upper-case letters, CMIP6 lower-case

In the *MT*2 model, the lack of correlation between OHU and AMOC in the $2\times \text{ CO}_{2}$ state of 1pctCO2 (point **2** of Sect. 2.12, Fig. 3b) is due to the anticorrelation of its components $H_{M}$ and $H_{T}$ (Fig. 13f). This in turn arises from the anticorrelation of *M* and *T* (point **3** of Sect 2.12, Sect. 3.2), in conjunction with the positive correlation of OHU with *M* and *T* individually. The anticorrelation of *M* and *T* can be seen in Fig. 12, in which the circles for CMIP5&6 AOGCMs lie broadly along the diagonal between low *M*–high *T* and high *M*–low *T*. Since this direction is parallel to contours of *H*, the AOGCM range of *H* is small, and since $N\propto H$, *N* has likewise a small spread across AOGCMs.

In other words, AOGCMs with stronger AMOC have greater high-latitude heat uptake $H_{M}$, but tend to have smaller heat uptake $H_{T}$ from the energy balance at low latitudes because of their small surface warming. This explanation in terms of the *MT*2 model is no more than a hypothesis. A deeper physical explanation is required to substantiate it, perhaps involving a link between properties of the ocean state and climate feedback (see also Sect. 6.3).

### Summary

In the *MT*2 model, global ocean heat uptake has two components, one depending on global-mean surface air temperature change *T*(*t*), the other on the piControl AMOC strength *M*. We hypothetically identify these as low- and high-latitude phenomena respectively. The *MT*2 model makes a good estimate of the timeseries of OHU for any individual AOGCM in either 1pctCO2 or abrupt4xCO2, given *T*(*t*) and *M*, and involving six AOGCM-neutral constant parameters. At the cost of making three of these AOGCM-specific (in the *MT*2*T* variant of the model), we can refine the OHU estimates.

The formulation of the *MT*2 model accounts for our earlier empirical findings that OHU is proportional to the rate of OHU across AOGCMs at a given time, and that about half of OHU is correlated with *T*, while the remainder is unrelated to *T* (points **4–6** of Sect. 2.12). Earlier, we found in addition that the effective climate sensitivity and the efficiency of removal of added tracers from the upper to the deep ocean are both related to *M* (points **8–9**). Given these points, the *MT*2 model accounts also for the lack of correlation of OHU and AMOC, and the anticorrelation of TCR and *M* (points **2–3**). The latter means that the two components of OHU tend to be anticorrelated across AOGCMs, which reduces the spread of the sum. Section 4.5 addresses the two remaining points (**1** and **7**) of Sect. 2.12.

## $MT2$ model of ocean heat uptake efficiency

In this section we evaluate and analyse the simulation of ocean heat uptake efficiency (OHUE, $\kappa =N/T$) in 1pctCO2 by the *MT*2 model (Sect. 3.1) and its *MT*2*T* variant (Sect. 3.1.4). The concept of OHUE is less useful for a scenario of constant forcing, such as abrupt4xCO2, because it is never approximately constant. Nevertheless, we gain some insight by considering the relationship between *N* and *T* in abrupt4xCO2 (Sect. 4.6).

### Evaluation of the $MT2$ model of OHUE

For the OHUE, the *MT*2 model (Eq. 5) gives

10 $$\begin{aligned} \kappa =\frac{N}{T}=\kappa _{M}+q \quad \quad \kappa _{M}=\frac{N_{M}}{T}=\frac{pF}{T}=\frac{s_{0}F(M-M_{0})}{T}. \end{aligned}$$

In these formulae, $s_{0}$ and $M_{0}$ are AOGCM-neutral constants, *M* is the AOGCM-specific piControl AMOC strength, and *q*(*t*) is computed by the two-layer component of *MT*2 or *MT*2*T*. In *MT*2, *q*(*t*) is AOGCM-neutral; in *MT*2*T* it is AOGCM-specific.

For the $2\times \text{ CO}_{2}$ state of 1pctCO2, *MT*2 $\kappa$ calculated from Eq. (10) for individual CMIP5&6 AOGCMs using their *M* and *T* correlates highly with AOGCM $\kappa$ ($r=0.88$, letters in Fig. 13a). *MT*2*T* reproduces AOGCM $\kappa$ more accurately than *MT*2 ($r=0.94$, blue in Fig. 13a, regression slope $0.98\pm 0.08$).

Likewise, the *MT*2 estimate of $\kappa (t)$ by Eq. (10) for individual AOGCMs correlates well with the AOGCM $\kappa (t)$ at any time in 1pctCO2 after the first couple of decades (green lines in Fig. 13b, $r>0.8$ after year 70). The *MT*2*T* estimate is even better (orange lines, $r>0.9$ after year 70), because of its slightly AOGCM-specific *q*. The correlation coefficient increases over time, as the forced signal becomes larger relative to unforced variability, despite the fact that $\kappa$ itself is decreasing.

Equation (10) makes a very good estimate of CMIP5&6 AOGCM-mean $\kappa (t)$ in 1pctCO2 using *q*(*t*) from either *MT*2 or *MT*2*T*. (The *MT*2 $\langle \kappa (t)\rangle$ in blue is very close to the AOGCM $\langle \kappa (t)\rangle$ in black in Fig. 13b.) In Sect. 4.3 we analyse the time-dependence of $\kappa$.

### AOGCM spread in OHUE

Substituting for *T* from Eq. (9) into Eq. (10) gives

11 $$\begin{aligned} \kappa _{M}= \frac{N_{M}}{T}=p F \,\frac{\alpha +q}{(1-p)F} =\frac{p(\alpha +q)}{1-p} \quad \quad \kappa = \kappa _{M} + q = \frac{p\alpha +q}{1-p}. \end{aligned}$$

For simplicity we do not show the small contribution to $N_{M}=\textrm{d}H_{M}/\textrm{d}t$ which comes from $U_{0}$ (Eq. 7), but we retain this term in the calculations. Equation (11) shows that $\kappa$ depends on *p*, $\alpha$ and *q*, but not on *F*.

In the *MT*2 model, *q* is AOGCM-neutral and thus contributes nothing to the spread of $\kappa$. *MT*2*T* *q* is AOGCM-specific but its correlation with AOGCM $\kappa$ is only 0.38 (in the $2\times \text{ CO}_{2}$ state of 1pctCO2). Therefore *q* explains only 15% of the variance of $\kappa$, and the majority comes from *p* and $\alpha$ through $\kappa _{M}$. Qualitatively the same is true at all times in 1pctCO2; the *MT*2*T* spread in *q* is always smaller than in $\kappa _{M}$ (orange and turquoise shading in Fig. 13c), and *q* has zero spread in *MT*2.

Consistent with this, the correlation coefficient is 0.79 between AOGCM $\kappa$ and the quantity $(p\alpha +\langle q \rangle )/(1-p)$, which is $\kappa$ from Eq. (11) calculated using AOGCM-mean *q*. That means *p* and $\alpha$ together explain $0.79^{2}\simeq 60$% of the spread of AOGCM $\kappa$. Excluding the spread of *p* as well as *q*, the quantity $(\langle p \rangle \alpha +\langle q \rangle )/(1-\langle p \rangle )$ has a correlation coefficient of 0.51 with $\kappa$ (for $2\times \text{ CO}_{2}$ in 1pctCO2), so $0.51^{2}\simeq 30$% of the variance of $\kappa$ comes from $\alpha$. Hence *p* and $\alpha$ contribute about equally to the spread of $\kappa$.

In summary, the spread of OHUE in the *MT*2 model is due roughly equally to the spread in the proportion *p* of the forcing absorbed at high latitude without affecting global-mean *T* (Sect. 3.3.1), and to the spread in the climate feedback parameter $\alpha$, which dominates the spread in *T* through the low-latitude energy balance (Sect. 3.3.3).

### Time-dependence of OHUE

The decline over time in OHUE in AOGCMs (black line in Fig. 13b,c) is reproduced by the *MT*2 model (blue line in Fig. 13b), where $\kappa =\kappa _{M}+q$. According to the *MT*2 model, the decline in *q* with time (orange line in Fig. 13c, halving in size over 100 years) is larger than in $\kappa _{M}$ (turquoise line).

In the two-layer model, OHUE ($N/T=\gamma (T-T_{d})/T$) declines because $T-T_{d}$ asymptotes to a constant while *T* rises continuously (Appendix B.3). The same occurs for $q=N_{T}/T$ in the two-layer component of *MT*2. By assuming that $T\propto t$, as in the zero-layer model, we can show that $q \propto 1/t$ approximately for the first few decades, thereafter declining exponentially on the deep-ocean timescale (Appendix C.4.5).

The time-dependence of $\kappa$ in 1pctCO2 can be visualised alternatively by considering the tangent slope of *N*(*T*), which decreases with time (grey lines in Fig. 13d have decreasing slopes). Again, the time-dependence comes mostly from $q=N_{T}/T$ (the thin orange lines are curved). If *q*(*t*) is AOGCM-neutral (as assumed by the *MT* and *MT*2 models), we expect $N_{T}=qT\propto T$ across AOGCMs at a given time. This behaviour can be seen in Fig. 13d. The 20-year means of $(T,N_{T})$ for individual AOGCMs (crosses) lie near to lines of constant *q* (in the corresponding colours).

In the *MT*2 model, $\kappa _{M}\propto F/T$ (Eq. 10). Since *T* increases slightly more rapidly than $F (\propto t)$ (Fig. 10a), $\kappa _{M}$ decreases, but only slowly (turquoise lines are nearly straight in Fig. 13d). From Eq. (11) we see that the decline in $\kappa _{M}$ has contributions from both $\alpha$ and *q*, which decrease at similar rates, although $\alpha$ is much larger (Fig. 13c).

### Fraction of the forcing absorbed by the ocean

By substituting for *T* from Eq. (9) into $N_{T}=qT$ (Eq. 5), we obtain

12 $$\begin{aligned} N_{T} = q T = \frac{q(1-p)}{\alpha +q}\,F. \end{aligned}$$

Hence, using $N_{M}=pF$ (Eq. 5),

13 $$\begin{aligned} \frac{N_{T}}{N_{M}} = \frac{q(1-p)}{(\alpha +q)}\frac{F}{pF}=\frac{q(1-p)}{(\alpha +q)p}. \end{aligned}$$

With $p=0.14\pm 0.03$ (Sect. 3.1), $\alpha =1.22\pm 0.36$ W m^-2^ K^-1^ (Table 1) and *MT*2 $q=0.36$ W m^-2^ K^-1^ for the $2\times \text{ CO}_{2}$ state of 1pctCO2, we obtain $N_{T}/N_{M}=1.6\pm 0.7$. This comparison is consistent with AOGCM-mean $H_{T}$ and $H_{M}$ being of similar size, with $H_{T}$ somewhat larger (Fig. 11b).

By substituting for $N_{T}$ from Eq. (12) into Eq. (5), we obtain

14 $$\begin{aligned}{} & {} N = N_{M} + N_{T} = \left( p+\frac{q(1-p)}{\alpha +q}\right) F = \frac{p\alpha +q}{\alpha +q}F \nonumber \\{} & {} \quad \Rightarrow \frac{N}{F} = \frac{p\alpha +q}{\alpha +q} \end{aligned}$$

Since the AOGCM spread in *q* is much less than in $\alpha$, and since $p\ll 1$, the spread of *N*/*F* is dominated by $\alpha$ in the denominator. Thus *N*/*F* and $1/\alpha$ are correlated across AOGCMs, or equivalently *N*/*F* and $\alpha$ are anticorrelated, according to the *MT*2 model. Williams et al. (2000) (their Figure 8d) show that $\alpha$ and *N*/*F* (our notation) are highly anticorrelated across CMIP5&6 AOGCMs in 1pctCO2. The correlation coefficient between $\alpha$ and *N*/*F* from Eq. (14) in the $2\times \text{ CO}_{2}$ state is $r=-0.85$. We find similarly strong anticorrelation, with *r* between $-0.8$ and $-0.9$ (not shown), for CMIP6 AOGCMs throughout the twenty-first century in projections following shared socioeconomic pathways (SSPs).

In summary, a larger fraction of the forcing is absorbed by the ocean in AOGCMs with smaller $\alpha$, which gives larger *T*, $N_{T}=qT$ and *N*. For the $2\times \text{ CO}_{2}$ state of 1pctCO2, Eq. (14) gives $N/F=0.34\pm 0.06$ i.e. about a third of the forcing is absorbed, with a spread of 18%, close to the spread of 15% we found for OHU (Sect. 2.7).

### Correlation of OHUE with the AMOC and $\alpha$

The positive correlation across CMIP5&6 AOGCMs of OHUE $\kappa$ with piControl AMOC *M* (point **1** of Sect. 2.12, Fig. 2d) emerges from Eq. (10) because *M* and *T* are anticorrelated (point **3** of Sect 2.12, Sect. 3.2), while the spread of *q* is small in *MT*2*T* and zero in *MT*2 (Sect. 4.2). *MT*2 and *MT*2*T* $\kappa$ calculated by Eq. (10) (for $2\times \text{ CO}_{2}$ in 1pctCO2) correlate even more strongly with *M* than AOGCM $\kappa$ does (Fig. 13e, *MT*2*T* is similar but not shown). The higher correlation could be due to the reduced influence of unforced variability.

In the alternative form given by Eq. (11), the correlation of $\kappa$ and *M* arises because the two factors *p* and $\alpha$ which cause the spread in $\kappa$ (Sect. 4.2) are both positively correlated with *M* (points **8–9** of Sect. 2.12, previously discussed in Sect. 3.2). Furthermore, the appearance of $\alpha$ in Eq. (11) explains the correlation of $\kappa$ with $\alpha$ (point **7**), which is reinforced by the correlation of $\alpha$ with *p* through *M*.

In summary, OHUE correlates with the AMOC because AOGCMs which have a stronger AMOC tend to have both more efficient high-latitude heat uptake (larger *p*), and lower effective climate sensitivity (larger $\alpha$), giving smaller global warming. OHUE is correlated with $\alpha$ because both are correlated with the AMOC. The strong correlation of OHUE with the AMOC is the counterpart of the insignificant correlation of OHU with the AMOC (Sect. 3.5).

### Relationship of *N* and *T* for abrupt4xCO2

**Fig. 14:**
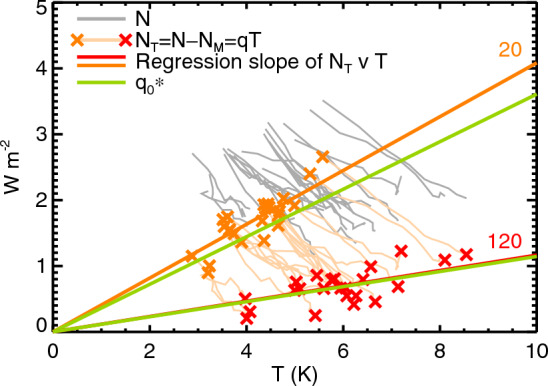
*N* and $N_{T}$ (Eq. 5) for individual CMIP5&6 AOGCMs as a function of *T* in abrupt4xCO2. Thin lines join successive 20-year means at 10-year intervals, orange and red crosses indicate 20-year means centred on years 20 and 120, thick lines in the same colour are regressions against *T*, and the green lines are from an approximate formula for the slopes (Eq. C47)

In the abrupt4xCO2 scenario, the forcing $F=F_{4\times }$ is constant, so the energy balance $N=F-\alpha T$ (Eq. 1) predicts that *N*(*t*) plotted against *T*(*t*) should give a straight line with slope $-\alpha$ if $\alpha$ is constant (Gregory et al. 2004). The trajectories of *N*(*T*) from CMIP5&6 AOGCMs (grey in Fig. 14) are actually not quite straight due to unforced variability and because $\alpha$ is not constant in abrupt4xCO2 (as has been studied in numerous works e.g. Andrews et al. 2012; Rugenstein et al. 2019; Dong et al. 2020). They are not parallel because $\alpha$ is AOGCM-specific.

The OHUE *N*/*T* is the slope of a line from the origin to (*N*, *T*). As *T* increases, OHUE decreases, more rapidly than in 1pctCO2. The estimates of OHUE by *MT*2 and *MT*2*T* for individual AOGCMs in abrupt4xCO2 are more accurate than in 1pctCO2, because of the larger forcing, except for an overestimate before year 20 ($r>0.8$ for AOGCM $\kappa$ and *MT*2 $\kappa$ after year 20, $r>0.95$ for *MT*2*T* throughout, not shown). The initial inaccuracy may be due an inadequacy of the two-layer approximation on short timescales, when the upper layer is not yet well-mixed.

In the *MT*2 model, $N_{M}$ is an AOGCM-specific constant in abrupt4xCO2, because *F* is constant (Eq. 5). Accordingly, the trajectory of $N_{T}=N-N_{M}$ for each AOGCM is displaced by its constant $N_{M}$ from and hence lies parallel to its *N* trajectory (thin orange and grey lines in Fig. 14). In the *MT*2 model, $N_{T}=q(t)T$, where *q*(*t*) is AOGCM-neutral. Hence $N_{T}\propto T$ across AOGCMs at a given time. This is approximately true, as illustrated by 20-year means centred on years 20 and 120 shown as crosses in Fig. 14, which lie near to the regression slopes for $N_{T}$ against *T* in the corresponding colours.

### Summary

The key points of this section are that the *MT*2 model attributes the AOGCM spread of OHUE at any given time in 1pctCO2 firstly to those characteristics of the ocean piControl state which are quantified by the piControl AMOC and remain constant in time, and secondly to the climate feedback parameter, which is also correlated with piControl AMOC. The *MT*2 model accounts for the strong correlation between OHUE and AMOC across AOGCMs at a given time, the lack of correlation between OHU and AMOC, and the anticorrelation of OHUE and effective climate sensitivity (points **1–2** and point **7** of Sect. 2.12). The decrease of the OHUE over time is due mostly to the warming of the deep ocean in the low-latitude heat balance, and partly to the decline in the climate feedback parameter, but is not related to piControl AMOC.

## $MT2$ model of tranient climate response

**Fig. 15:**
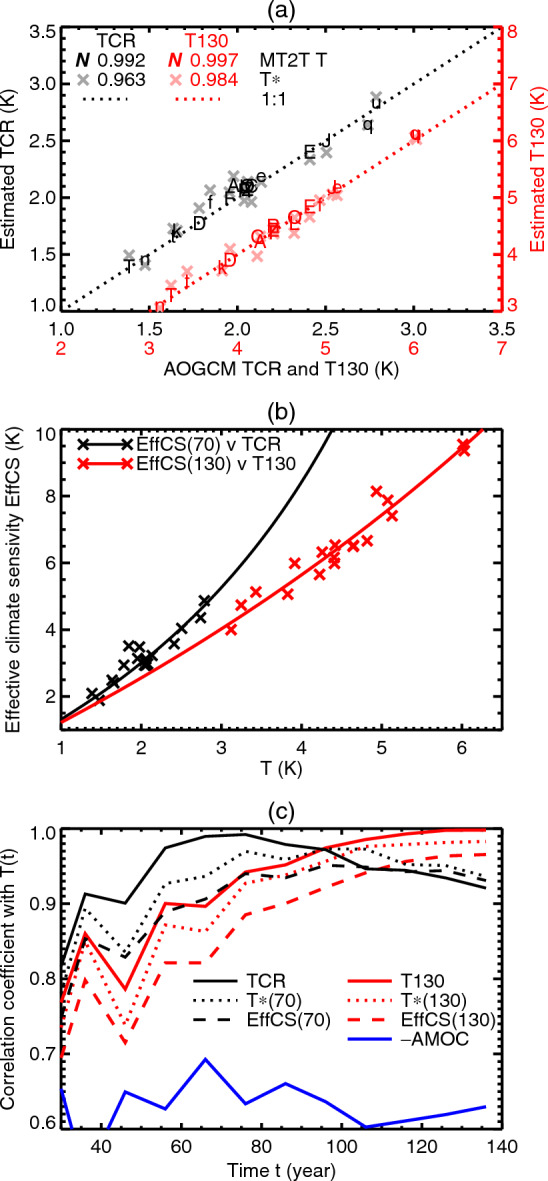
**a** TCR and T130 in 1pctCO2 experiments (*T* for 20-year means centred on years 70 and 130 respectively), with letters identifying AOGCMs according to Table 1 (CMIP5 with upper-case letters, CMIP6 lower-case), compared and correlated with *MT*2*T* *T* (Eq. 9) and $T^{*}$ (Eq. 15, an estimate of *T* which neglects the AOGCM spread of OHUE). The red numbers on the horizontal axis are for T130, the black for TCR. The right-hand axis is shifted by 1 K so that TCR and T130 symbols can be seen separately. **b** Effective climate sensitivity (EffCS) for years 70 and 130 in 1pctCO2 estimated from *F*, *N* and *T* (TCR and T130 respectively) for individual AOGCMs and plotted against *T*, with lines showing the relationships expected from the approximate *MT*2 formula (Eq. 20). **c** Correlation of *T*(*t*) across AOGCMs as a function of time in 1pctCO2 with TCR and T130, with $T^{*}$ and EffCS evaluated at years 70 and 130, and with the AMOC strength in piControl (multiplied by $-1$ to give a positive number)

In this section we analyse AOGCM *T*(*t*) in 1pctCO2 using the *MT*2 model, and its *MT*2*T* variant with AOGCM-specific *q*(*t*).

### AOGCM spread of *T*

To analyse *T*(*t*) in 1pctCO2, we need to know $\alpha (t)$, which we estimate as $(F-N)/T$ using 20-year means of *T*(*t*) and *N*(*t*). We assume that $F(140)=F_{4\times }$ (the forcing in abrupt4xCO2, Table 1, obtained by the method of Gregory et al. (2004)) and that $F(t)\propto t$ during 1pctCO2. The latter is not exactly true (Bloch-Johnson et al. 2021) but no experiments exist to diagnose *F*(*t*) in CMIP5&6. We get $\alpha =1.30\pm 0.44$ W m^-2^ K^-1^ at year 70 and $\alpha =1.16\pm 0.37$ W m^-2^ K^-1^ at year 130 (Fig. 13c); $\alpha$ has a larger AOGCM spread than $\kappa$ and decreases in time in 1pctCO2 (Gregory et al. 2015).

Using AOGCM-specific *M*, $\alpha (t)$ and *MT*2*T* *q*(*t*), *T* from Eq. (9) at years 70 and 130 gives an accurate estimate of TCR and T130 for individual AOGCMs (letters in Fig. 15a, $r>0.99$ for both, root-mean-square $T(70)-\text{ TCR }$ is 0.04 K, $T(130)-\text{ T130 }$ is 0.14 K), where T130 is the time-mean AOGCM *T* for years 121–140. (These years are the last 20 of CMIP5 1pctCO2.)

Because $\alpha$ contributes most of the spread (Fig. 13c), we also consider

15 $$\begin{aligned} T^{*}=\frac{1-\langle p\rangle }{\alpha +q}F, \end{aligned}$$

in which only $\alpha$ is AOGCM-specific. Equation (15) has the same form as the zero-layer model (Eq. 2), with *F* reduced by $\langle p \rangle =14$% on account of the *M*-dependent part of OHU, and the AOGCM-specific OHUE $\kappa$ replaced by the AOGCM-neutral *MT*2 *q*. For *q* we use $q=q_{1}^{*}$ of Eq. (C53) derived from the AOGCM-mean $\langle T(t)\rangle$ in Appendix C.4.4.

$T^{*}$ is almost as accurate as *T* of Eq. (9) (crosses in Fig. 15a, $r=0.965$ and RMS error 0.10 K for TCR, $r=0.982$ and 0.18 K for T130). This demonstrates that the spread in *T* is mostly determined by $\alpha$.

### Time-dependence of climate resistance

The TCR was invented (Cubasch et al. 2001) as a benchmark for AOGCMs in time-dependent projections, analogous to the equilibrium climate sensitivity for steady states, implicitly assuming the climate resistance $\rho \equiv F/T$ to be a time-independent property of an AOGCM. If $\rho$ is constant, it can be evaluated from any transient state e.g. year 70 in 1pctCO2 as $\rho =F_{2\times }/\text{TCR }$, and then used to estimate $T=F/\rho =\text{ TCR }\times F/F_{2\times }$ for any other *F*.

In CMIP5&6 AOGCMs, both $\alpha$ and *q* decrease as time passes (Fig. 13c). Hence the climate resistance (Gregory and Forster 2008)

16 $$\begin{aligned} \rho \simeq \frac{F}{T^{*}}=\frac{\alpha +q}{1-\langle p\rangle } \end{aligned}$$

also decreases.

Since $F\propto t$ in 1pctCO2, Eq. (15) or Eq. (16) gives

17 $$\begin{aligned} \frac{T^{*}(t_{2})}{T^{*}(t_{1})} =\frac{F(t_{2})}{\rho (t_{2})}\,\frac{\rho (t_{1})}{F(t_{1})} = \frac{t_{2}}{t_{1}} \frac{\alpha (t_{1})+q(t_{1})}{\alpha (t_{2})+q(t_{2})} \end{aligned}$$

for any pair of times $t_{1},t_{2}$. With $t_{1}=70$ years and $t_{2}=140$ years, for which $T(t_{1})=\text{ TCR }$ and $T(t_{2})=\text{ T140 }$,

18 $$\begin{aligned} \frac{\text{ T140 }}{\text{ TCR }}\simeq \frac{T^{*}(140)}{T^{*}(70)} =\frac{140}{70}\frac{\alpha (70)+q(70)}{\alpha (140)+q(140)}>2 \end{aligned}$$

because $\alpha (70)>\alpha (140)$ and $q(70)>q(140)$. This is why the increase in *T*, from TCR to T140, during the second doubling of CO_2_ in 1pctCO2, is larger than the TCR (the increase in *T* under the first doubling), although the change in *F* is the same for both first and second doubling (Gregory et al. 2015). The decline in $\alpha$ and *q* is sufficiently rapid that $T(130)/\text{TCR }>2$ as well. (This is shown by the red letters being to the right of the black letters in Fig. 15a, except for CanESM2, marked “E”, in which $\alpha$ does not decline.)

### Relationship between TCR and EffCS

Using $\alpha +q=(1-\langle p\rangle )F/T^{*}$ from rearranging Eq. (16), we get

19 $$\begin{aligned} \text{ EffCS }(t)= & {} \frac{F(t)}{\alpha (t)}\simeq \frac{F}{(1-\langle p\rangle )F/T^{*}-q} \nonumber \\= & {} \frac{T^{*}}{1-\langle p\rangle -(q/F)T^{*}} \nonumber \\= & {} \frac{T^{*}}{1-\langle p\rangle -(qt_{4\times }/F_{4\times }t)T^{*}} \end{aligned}$$

where $t_{4\times }=140$ years, and hence

20 $$\begin{aligned}{} & {} \text{ EffCS }(70)=\frac{\text{ TCR }}{0.86-0.095\,\text{ TCR }} \nonumber \\{} & {} \quad \text{ EffCS }(130)=\frac{\text{ T130 }}{0.86-0.036\,\text{ T130 }}, \end{aligned}$$

given $F_{4\times }=7.5$ W m^-2^ and $q(70,130)=0.36,0.25$ W m^-2^ K^-1^. These formulae produce good fits to EffCS as a function of *T* (Fig. 15b) with errors of $0.06\pm 0.28$ K in EffCS(70), $0.01\pm 0.37$ K in EffCS(130).

### Late twenty-first-century *T* correlates better with EffCS than with TCR

Gregory et al. (2015) and Grose et al. (2018) showed that *T* in the late twenty-first century under a scenario of continuously increasing *F* correlates more highly with the effective climate sensitivity $\text{ EffCS }=F_{2\times }/\alpha _{4}$ than with TCR, where $\alpha _{4}$ is the climate feedback parameter diagnosed from abrupt4xCO2 (Gregory et al. 2004). This was a surprise, because $\text{ TCR }=F_{2\times }/(\alpha +\kappa )$ contains information about the AOGCM spread of $\kappa$, which is relevant to transient states, whereas EffCS does not.

The surprise can be illustrated by considering T130 as an idealised analogy to the late twenty-first century *T* in a scenario of high CO_2_ emissions. The surprising result is that the correlation of $\text{ T130 }=F(130)/\rho (130)$ is stronger with $\text{ EffCS }(130)=F_{4\times }/\alpha (130)$ than with $\text{ TCR }=F(70)/\rho (70)={\textstyle \frac{1}{2}}F_{4\times }/\rho (70)$ (Fig. 15c, compare dashed red and solid black lines at $t=130$ yr). With the *MT*2 model, we can account for this by considering

21 $$\begin{aligned} \rho = \frac{F}{T} = \alpha + \kappa = \frac{\alpha +q}{1-p} \end{aligned}$$

from either Eq. (9) or Eq. (11). *F* affects *T*, but it varies in time in the same way in all AOGCMs. Hence the time-variation in the correlation of *T* across AOGCMs between two different times (Fig. 15c) is due to the behaviour of $\rho$.

The AOGCM spread in $\rho$ at any time is due mostly to $\alpha$ (Fig. 13c). On the right-hand side of Eq. (21), the time-variation of AOGCM-*mean* $\rho$ is due more to *q* than $\alpha$, but *q* has a smaller spread. (Note that *p* is constant in time.) That means all AOGCMs have similar time-variation of *q*, whereas the time-variation of $\alpha$ differs more widely among AOGCMs. Hence the time-variation of the AOGCM *spread* in $\rho$ is due more to $\alpha$ than *q*, and the correlation of $\rho$ between different times depends mostly on the correlation of $\alpha$ between those times. Equivalently, the correlation of *T* between different times depends mostly on that of EffCS.

T130 correlates better than EffCS(130) does with *T*(*t*) at any time (the solid red line is always above the dashed red line in Fig. 15c) because T130 includes the AOGCM spread of *q*. Nonetheless T130 correlates worse with $\text{ TCR }=T(70)$ than with EffCS(130) because the time-dependence of $\alpha$ is more important in this comparison than the AOGCM spread of *q*. In other words, if EffCS is estimated with $\alpha$ from a climate state like the late twenty-first century, it correlates more highly with *T* at that time than TCR does, because TCR is estimated with $\alpha$ from a climate state which is like a much earlier time. A similar explanation was given by Gregory et al. (2015), but they did not have a model for the time-dependence of OHUE.

### Summary

The key points of this section are that the AOGCM spread of *T* and climate resistance at a given time in 1pctCO2 is due primarily to climate feedback $\alpha$, leading to simple and quite accurate relationships for *T* as a function of $\alpha$, and effective climate sensitivity as a function of *T*. The decrease of the AOGCM-mean climate resistance with time in 1pctCO2 is due to the AOGCM-mean decrease in both $\alpha$ and OHUE, but the AOGCM spread in the time-variation of climate resistance is due mostly to $\alpha$. Because $\alpha$ dominates the spread in climate resistance at any time and its variation over time, *T* correlates more strongly with $\alpha$ at the same time than with *T* at another time.

## Summary and discussion

In this work we have studied the ocean heat uptake efficiency in time-dependent climate change (OHUE, $\kappa$, W m^-2^ K^-1^). We have analysed CMIP5&6 AOGCMs in 1pctCO2 (CO_2_ increase at 1% yr^-1^), abrupt4xCO2 (instantaneous quadrupling), faf-heat and faf-passiveheat experiments (from the FAFMIP protocol, with imposed constant surface fluxes of heat and passive tracer, respectively). OHUE is defined as *N*/*T*, where *N* is the rate of increase of ocean heat content (W per m^2^ of the global area) and *T* the global-mean surface air temperature change with respect to the unperturbed climate. For all AOGCMs and both CO_2_-forced scenarios, $T=1.5\times$ the global-mean SST change, to a very good approximation (*cf.* Toda et al. 2021).

OHUE is usually evaluated for the $2\times \text{ CO}_{2}$ state (year 70) in the 1pctCO2 experiment. For a few decades in 1pctCO2, but not in general, the OHUE is the same as the thermal coupling between the upper ocean and the deep ocean of the two-layer global ocean model often used to interpret and emulate AOGCM simulations of ocean heat uptake (OHU, *H*, ZJ). OHUE has previously been found to correlate across AOGCMs with the piControl strength of the Atlantic meridional overturning circulation (AMOC) (Kostov et al. 2014; Winton et al. 2014). However, the AMOC itself is not the major process of OHU (Couldrey et al. 2023), and our analysis suggests a different explanation for this correlation.

### The $MT2$ model

To account for the AOGCM results, we propose a new conceptual model of global OHU, called *MT*2 (Fig. 8c, Appendix E). The *MT*2 model extends the two-layer ocean model to incorporate the connection of OHUE with the AMOC. In the *MT*2 model, a proportion

22 $$\begin{aligned} p=s_{0}(M-M_{0}), \end{aligned}$$

of the effective radiative forcing *F* due to atmospheric CO_2_ is stored in the ocean with no effect on *T*, where $s_{0}$ and $M_{0}$ are AOGCM-neutral constants, and by “AOGCM-neutral” we mean that the *MT*2 model uses the same value for all AOGCMs. The values of the AOGCM-neutral constants of the *MT*2 model are shown in the table in Appendix E.

The piControl AMOC strength *M* and hence also the proportion *p* (Eq. 22) are AOGCM-specific constants i.e. having a different value for each AOGCM. Note that $M_{0}<0$, so $p>0$ for any AOGCM, since all have $M>0$. The CMIP5&6 AOGCMs that we have analysed give $p=0.14\pm 0.03$ (AOGCM ensemble mean and standard deviation).

We hypothesise that the removed forcing $N_{M}=p F$ describes high-latitude OHU (Sect. 3.3.1), especially in the Southern Ocean, with little effect on global-mean *T* (see Sect. 3.3.3). These processes occur predominantly along neutral directions, as if heat were a passive tracer, by mesoscale eddy transports and wind-driven circulation; their interaction is complex and their realistic representation in GCMs is a long-standing challenge (Beadling et al. 2020; Hewitt et al. 2020). We hypothesise that the correlation of $N_{M}$ with the AMOC arises because both are sensitive to some other AOGCM-specific property of the unperturbed ocean state, possibly related to stratification (Newsom et al. 2023) and mesoscale eddy advection (see Sect. 3.3.2).

In the *MT*2 model, the global energy balance (quoting Eq. 8) is

23 $$\begin{aligned} F - \alpha T = N = N_{M} + N_{T} \end{aligned}$$

where

24 $$\begin{aligned}{} & {} N_{M} = p F, \quad \quad N_{T} = qT = c_{u}\frac{\textrm{d}T}{\textrm{d}t} + \gamma (T-T_{d}), \nonumber \\{} & {} \quad \quad c_{d}\frac{\textrm{d}T_{d}}{\textrm{d}t} = \gamma (T-T_{d}) \end{aligned}$$

and *F* is the effective radiative forcing. The climate feedback parameter $\alpha$ (a positive number according to our definition) is time-dependent and AOGCM-specific, for many reasons that are the subject of other research (e.g.Winton et al. 2010; Andrews et al. 2012, 2015; Ceppi and Gregory 2019; Zelinka et al. 2020; Sherwood et al. 2020). We hypothesise that $N_{T}$ describes ocean heat uptake at low latitude, which is driven by surface warming. Low-latitude warming dominates global *T* and is strongly influenced by climate feedback, hence $\alpha$ affects $N_{T}$.

The term $N_{T}$ has the same form as in the two-layer model (Fig. 8b); $c_{u},c_{d}$ are the heat capacities and $T,T_{d}$ the temperature changes relative to the unperturbed state of the upper and deep ocean respectively, $\gamma$ is the thermal coupling coefficient between them, and $q\equiv N_{T}/T$ is a scenario-dependent but AOGCM-neutral function of time. (It is AOGCM-neutral because *T*(*t*) has a similar time-profile in all AOGCMs, itself essentially because $\gamma \ll \alpha$; see Appendix C.4.4.)

The coefficients $c_{u},c_{d},\gamma ,s_{0},M_{0}$, and a sixth coefficient $U_{0}$ (qualitatively unimportant and therefore not discussed further in this summary) are all AOGCM-neutral and scenario-independent. We have evaluated them to give the best fit for the AOGCM-mean of CMIP5&6 abrupt4xCO2 experiments. In contrast, $c_{u},c_{d},\gamma$ are AOGCM-specific in the usual two-layer model (e.g.Geoffroy et al. 2013b; Gregory et al. 2015).

Given the time-mean piControl AMOC and the time-dependent global-mean surface temperature *T*(*t*) from any AOGCM in either CO_2_ scenario, the *MT*2 model accurately reproduces *N*(*t*), OHU $H(t)=\int N(t')\,\textrm{d}t'$, and the time-dependent OHUE $\kappa (t)$. The version called *MT*2*T*, which has AOGCM-specific $c_{u},c_{d},\gamma$ (as in the two-layer model of e.g. Geoffroy et al. (2013b)), is more accurate, but that does not affect any of our qualitative conclusions. In *MT*2*T*, *q* has a small spread among AOGCMs, arising from $c_{d}$.

### New findings of this work

In this section, we summarise our new findings, and we account for these, and for some previously unexplained results, in terms of the *MT*2 model. **Bold text** highlights the main points.

In the *MT*2 model, a proportion *p* of the forcing *F* is absorbed by $N_{M}=p F$ without affecting *T*. Hence only $F-N_{M}$ remains to enter the energy balance

25 $$\begin{aligned} F-N_{M}=F(1-p)=\alpha T+N_{T} =(\alpha +q)T. \end{aligned}$$

(from Eq. 23 and 24). **In an AOGCM with a stronger piControl AMOC, heat is removed more effectively from the upper ocean at high latitude, especially in the Southern Ocean** i.e. *p* and $N_{M}$ are larger for a given *F*. This is **because the relevant processes are affected by some global characteristic of the unperturbed state of the ocean, perhaps related to stratification, which also affects the AMOC**. When AMOC and $N_{M}$ are larger, the heat available ($F-N_{M}$) to cause surface global warming is smaller. This makes *T* smaller, for a given $\alpha$, if AMOC is stronger. We find furthermore that $\alpha$ is larger i.e. **effective climate sensitivity is smaller in AOGCMs with a stronger AMOC**. This is another reason for *T* to be smaller if AMOC is stronger. Consequently the **transient climate response** (TCR, defined as *T* for $2\times \text{ CO}_{2}$ in 1pctCO2) **and local sea-surface temperature change worldwide are both negatively correlated with piControl AMOC strength across AOGCMs**.

For a given *F*, since *T* is negatively correlated with AMOC strength, so also is $N_{T}=qT$, *q* being AOGCM-neutral. On the other hand, *p* and hence $N_{M}=pF$ are positively correlated with AMOC strength. Thus $N_{T}$ and $N_{M}$ are anticorrelated across AOGCMs, since they have opposite variations with AMOC strength. The anticorrelation of $N_{M}$ and $N_{T}$ leads to a relatively small AOGCM spread in the net rate of heat uptake $N=N_{M}+N_{T}$, and *N* does not correlate with AMOC.

Therefore, since OHU is the time-integral of *N*, **global OHU does not correlate significantly with AMOC**, because high- and low-latitude OHU are oppositely correlated with AMOC. By contrast, the **ocean heat uptake efficiency** (OHUE $\kappa =N/T$) **is correlated with the piControl AMOC strength** (Kostov et al. 2014; Winton et al. 2014) **because global warming is anticorrelated with piControl AMOC**. However, **the AOGCM spread in OHUE is not causally related to the** ***change*** **in strength of the AMOC during transient climate change**.

Through its relationships with heat uptake and climate feedback, the unperturbed AMOC strength “explains” (in a statistical sense) 80% of the variance of OHUE in 1pctCO2 across AOGCMs, and about 40% of the TCR variance. **The AOGCM spread in OHUE is due roughly equally to the spread in efficiency of passive tracer uptake and the spread in the effective climate sensitivity**, both of which are correlated with the AMOC. Unlike previous authors, we find a significant correlation between $\alpha$ and $\kappa$, due to our larger set of AOGCMs, and our formulae for evaluating these quantities, excluding the confounding effects of time-variation. That means **OHUE and effective climate sensitivity are anticorrelated**.

By assuming the physical basis for the relationship of AMOC and OHUE in AOGCMs to be realistic, observational estimates of the former might be used to set an “emergent constraint” on the latter, like with ECS (e.g. Caldwell et al. 2018). For the observationally estimated time-mean AMOC of 2004–2014, the corresponding OHUE is 0.68 W m^-2^ K^-1^ (Appendix A.1; Fu et al. (2020); Worthington et al. 2021), which is consistent with $0.58\pm 0.08$ W m^-2^ K^-1^ calculated from ocean and surface temperature for the last five decades (Cael 2022), and close to the AOGCM-mean OHUE of 0.70 W m^-2^ K^-1^ for the $2\times \text{ CO}_{2}$ state of 1pctCO2. Some CMIP5&6 AOGCMs deviate substantially from the observational estimates.

Even with perfect models, we should not expect historical and 1pctCO2 OHUE to be exactly equal, because OHUE declines as a function of time. Although the processes related to temperature-driven heat uptake at low latitude, represented by the two-layer model, contribute little to the spread of OHUE across AOGCMs at a given time, the time-dependence of OHUE arises primarily from temperature-driven heat uptake. In terms of the *MT*2 model, it is due to the decline of *q*. In terms of the two-layer model, it comes about because the temperature difference between the layers decreases as the deep layer warms up; it is not necessary to make the thermal coupling $\gamma$ depend on time in order to account for the behaviour of OHUE. In the *MT*2 model, the OHUE $\kappa =q+p F/T$, and in 1pctCO2, both *F* and *T* increase linearly with time (to a fair approximation), so *pF*/*T* is an AOGCM-specific constant. Because of this nearly constant part, in fractional terms the OHUE declines more slowly in 1pctCO2 than the two-layer model would suggest. That explains why the zero-layer model, with its assumption of constant AOGCM-specific $\kappa$, works fairly well for 1pctCO2.

The decrease of $\alpha$ over time also contributes to the decline of OHUE (through *T*), but *q* is more important for the *AOGCM-mean decrease* in OHUE. However, because *q* has little spread among AOGCMs at any time, the *AOGCM spread in the decrease* of $\alpha$ over time dominates the AOGCM spread both in the decrease of OHUE over time, and in the decrease of climate resistance *F*/*T* over time. This rather subtle point leads us to infer that **projected global warming in the twenty-first century correlates more strongly across AOGCMs with the effective climate sensitivity than with the TCR** (Grose et al. 2018) b**ecause the AOGCM spread in the time-variation of effective climate sensitivity is the main influence on the spread in the time-variation of OHUE as well**.

### Concluding remarks

We conclude by listing some unanswered questions and possible next steps.

$\bullet$ Our results corroborate earlier work (e.g. Marshall and Zanna 2014; Exarchou et al. 2015) that demonstrates important roles for global OHU from wind-driven extratropical heat uptake, eddy-induced neutral diffusion and the Southern Ocean. Further analysis (following Saenko et al. 2021) is needed to relate these and other processes of vertical heat transport to the high-latitude *F*-dependent $N_{M}$ and low-latitude *T*-dependent $N_{T}$ of the *MT*2 model. This research includes discovering how the emergent and parametrised processes in AOGCMs determine the values of the *MT*2 model’s six coefficients, and why these coefficients are fairly AOGCM-neutral.

$\bullet$ Since the *MT*2 model outlined in this paper works well for the idealised 1pctCO2 and abrupt4xCO2 scenarios, it is probably applicable to socioeconomic scenarios for projecting the twenty-first century, but this should be tested. After some time it must become inaccurate, in particular because $N_{M}$ cannot remain non-zero and unchanged as equilibrium is approached under constant forcing. Considering that anthropogenic aerosol forcing is focussed on northern hemisphere extratropics, while greenhouse-gas forcing is greatest at low latitude (e.g. Salvi et al. 2022), it is also important to investigate whether the nature of the forcing influences the relative importance of the low- and high-latitude processes and hence the ocean heat uptake efficiency and global-mean *T*.

$\bullet$ In this work, we calibrate the *MT*2 two-layer model parameters using *T*(*t*) diagnosed from AOGCMs. It would be possible to use the *MT*2 model to emulate AOGCM *N*(*t*) and *T*(*t*), given the piControl AMOC strength *M*, the effective radiative forcing *F*(*t*), and the climate feedback parameter $\alpha (t)$. The latter is the main difficulty, because its time-variation has not yet been understood. It is possible that the commonly used closure assumption for $\alpha$ via the efficacy of ocean heat uptake (Winton et al. 2010; Geoffroy et al. 2013a) is related to the roles of high and low latitudes in heat uptake as analysed in this paper.

$\bullet$ We need to identify the characteristic of the ocean state which causes the correlation across AOGCMs of the unperturbed AMOC strength *M* with the passive tracer uptake efficiency, especially in the Southern Ocean (point **9** of Sect. 2.12). If eddy parametrisations are involved, an interesting question is whether the spread of uptake efficiency is smaller in eddy-resolving AOGCMs. Historical observations of passive tracers or the climatological mean state might provide a test or constraint of the relevant aspects of AOGCMs.

$\bullet$ We need to explain the correlation of *M* and the climate feedback parameter (point **8** of Sect. 2.12). This explanation might be connected to the correlation of *M* with the *time-variation* of climate feedback parameter in abrupt4xCO2 (Lin et al. 2019). It could involve the correlation of *M* with local sea-surface temperature, or with the change in this quantity, especially in sensitive regions for climate feedback processes.

$\bullet$ We need to investigate whether the anticorrelation of $N_{M}$ and $N_{T}$ in the *MT*2 model is the correct physical explanation for the relatively small spread of OHU in AOGCMs.

The last three points are elements of the wider subject of the relationship between heat uptake and climate sensitivity. Although often regarded as intrinsic to ocean and atmosphere respectively, they are actually linked as aspects of the coupled climate system.

## Figures and Tables

**Fig. 16:**
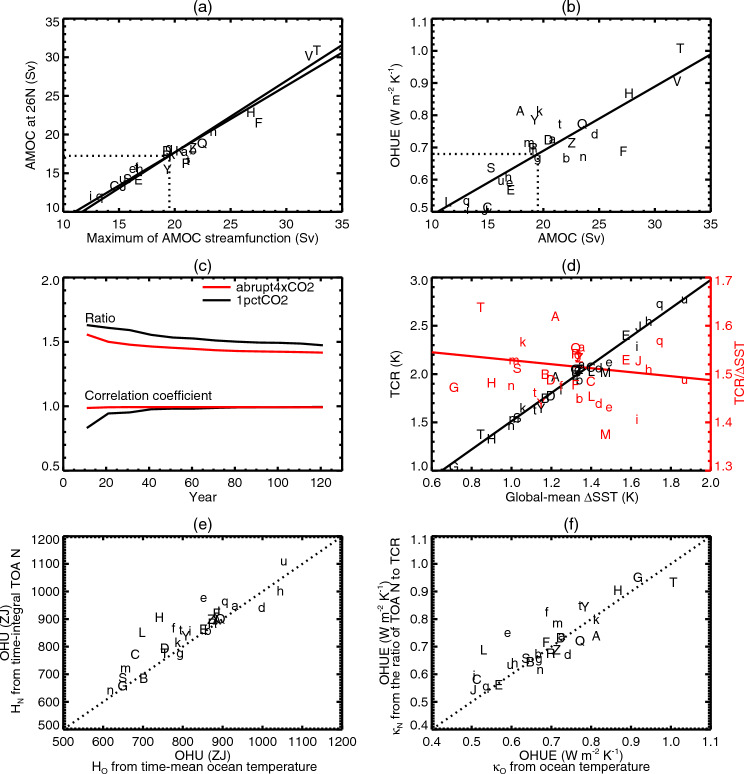
**a** Relationship between the AMOC at 26^∘^  N (the maximum of the overturning streamfunction in depth at that latitude) and *M* in our analysis (the maximum of the overturning streamfunction north of 30^∘^  N and within 500–2000 m depth), with ordinary least-squares regressions of each against the other shown by solid lines, **b** relationship between *M* and OHUE (the same data as in Fig. 2d), with solid regression lines. **c** Correlation and ratio between global-mean surface air temperature change and ocean area-mean sea surface temperature change as a function of time in abrupt4xCO2 and 1pctCO2, **d** relationship between the global-mean surface air temperature (TCR) and global-mean sea-surface temperature change ($\Delta$SST) at the time of $2\times \text{ CO}_{2}$ in 1pctCO2, with the regression line, and the ratio of the quantities (in red), **e**, **f** comparison of OHU and OHUE in CMIP5&6 AOGCMs estimated from ocean temperature, as used in our analysis, with the corresponding quantities evaluated from top-of-atmosphere net downward radiation and surface air temperature. In **a** and **b** the dotted lines connect the observational estimate of AMOC at 26^∘^  N with OHUE. In **e** and **f** the dotted lines are 1:1. In all panels, letters identify CMIP5&6 AOGCMs according to Table 1, CMIP5 with upper-case letters, CMIP6 lower-case

**Fig. 17:**
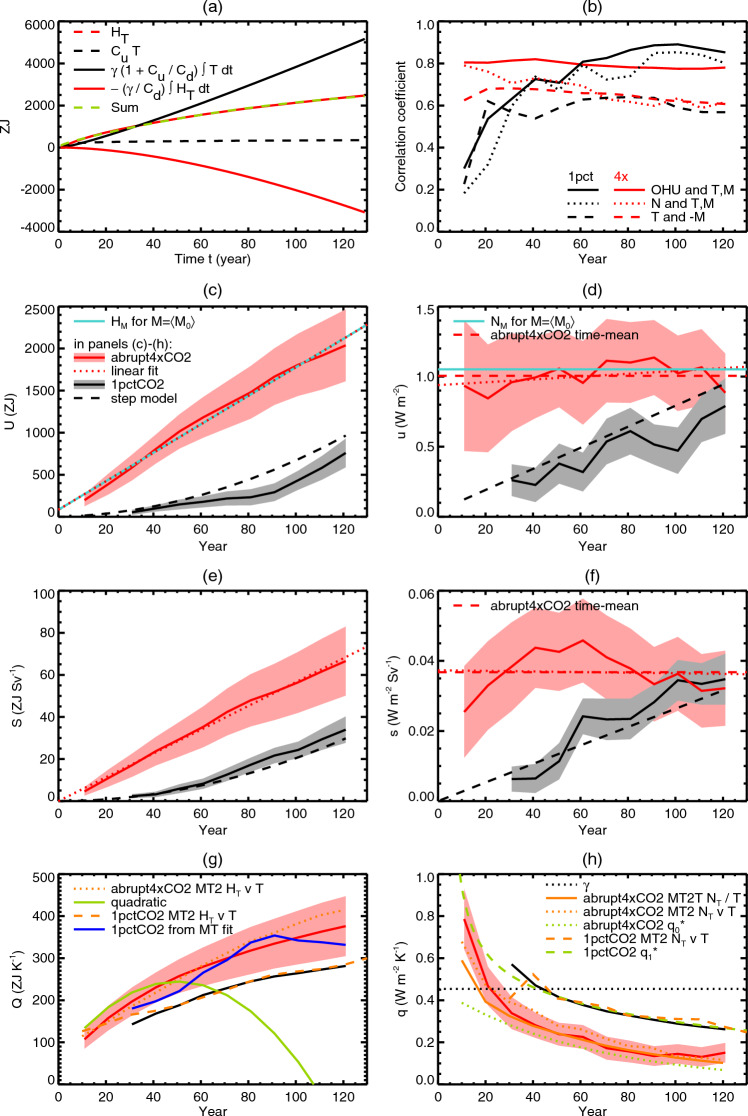
**a** Multiple linear regression of AOGCM-mean $H_{T}$ against *T*, $\int T\,\textrm{d}t$ and $\int H_{T}\,\textrm{d}t$ to obtain the parameters of the *MT*2 fit. **b** Multiple correlation coefficients of the *MT* model as a function of time and scenario in CMIP5&6 experiments. **c**–**h** Time-variation of parameters and variables of the *MT* model, compared with other quantities as described in the text

**Fig. 18:**
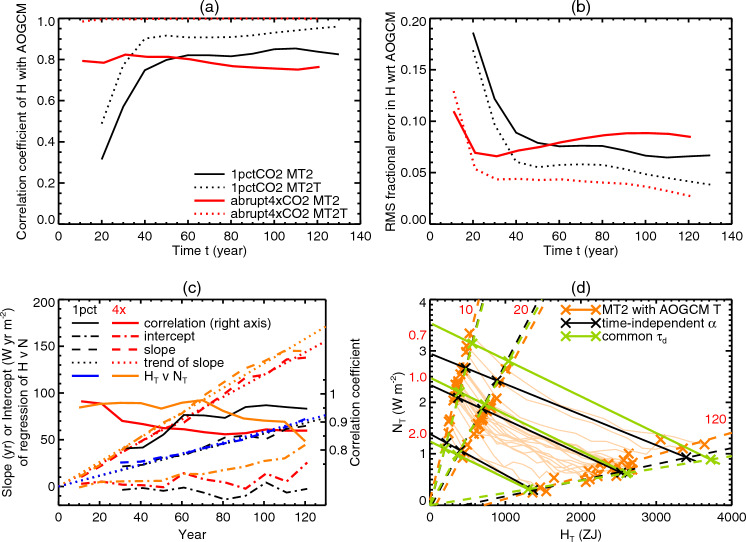
**a** Correlation of AOGCM *H* with *MT*2 (solid) and *MT*2*T* (dotted) $H_{MT}$ across AOGCMs as a function of time in 1pctCO2 (black) and abrupt4xCO2 (red). **b** Root-mean-square error in $H_{MT}$ relative to AOGCM *H* divided by AOGCM-mean *H* as a function of time, using the same key as **a**. **c** Correlation and regression of *H* against *N* across AOGCMs as a function of time in 1pctCO2 (black) and abrupt4xCO2 (red), and the slope of the regression across AOGCMs of $H_{T}$ against $N_{T}$ in 1pctCO2 (blue) and abrupt4xCO2 (orange), using the same linestyles as for *H*(*N*). **d** Relationship of $H_{T}$ to $N_{T}$ as time varies in abrupt4xCO2 calculated by the two-layer model using each AOGCM’s *T*(*t*) and two-layer model parameters (solid orange lines), compared with $H_{T}(N_{T})$ from the two-layer model using its own *T*(*t*) for three time-independent values of $\alpha$ (solid black lines, $\alpha$ marked in red along the left axis), and calculated again for the same $\alpha$ but assuming a common $\tau _{d}$ independent of $\alpha$ (solid green lines). The symbols show $(N_{T},H_{T})$ at the years indicated in red along the top and right sides, and the dashed lines show regressions of $H_{T}$ against $N_{T}$ across AOGCMs for those years

**Fig. 19:**
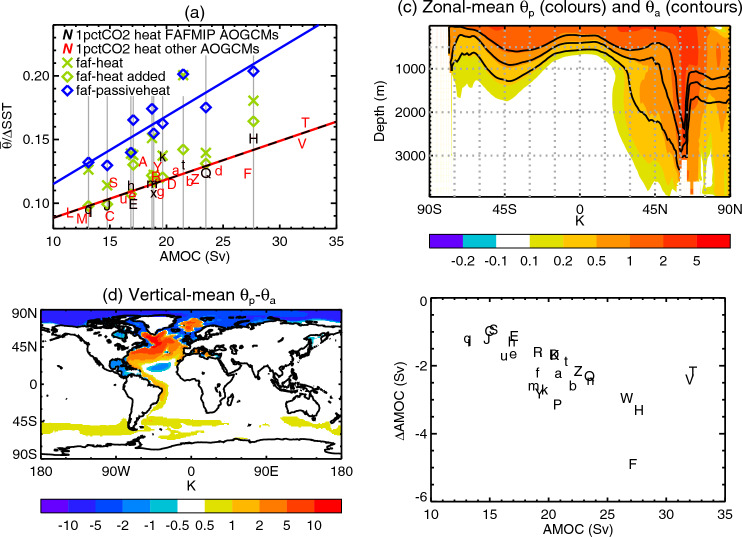
**a** Relationship across CMIP5&6 AOGCMs of $\overline{\theta }/\Delta \textrm{SST}$ in 1pctCO2 and FAFMIP faf-heat, $\overline{\theta }_{a}/\Delta \text{ SST}_{a}$ in faf-heat, and $\overline{\theta }_{p}/\Delta \text{ SST}_{p}$ in faf-passiveheat, with the AMOC strength in piControl. This figure is the same as Fig. 6b with the addition of faf-heat results. The thin grey vertical lines indicate the AMOC values and connect the symbols for FAFMIP AOGCMs. **b**, **c** Comparison of last-decade AOGCM-mean added heat $\theta _{a}$ in the faf-heat experiment with passive heat $\theta _{p}$ in faf-passiveheat. **d** Relationship across CMIP5&6 AOGCMs between piControl AMOC strength and the change in AMOC in 1pctCO2 (time-mean of years 1–70 minus piControl time-mean). Letters in **a** and **d** identify CMIP5&6 AOGCMs according to Table 1, CMIP5 with upper-case letters, CMIP6 lower-case

## Data Availability

The CMIP5 and CMIP6 AOGCM data analysed in this work are available on the Earth System Grid.

## Appendix A Evaluation of diagnostics from AOGCM ocean data

### A.1 AMOC

Rather than choosing a particular latitude, we evaluate the AMOC strength *M* in AOGCMs as the maximum of the Atlantic Ocean overturning streamfunction wherever it occurs in the northern extratropics, to reduce the effect of AOGCM-specific climatological biases. Thus defined, *M* is highly correlated ($r=0.97$, Fig. 16a) with the AOGCM overturning strength at 26^∘^  N, the latitude of the RAPID array, which has monitored the AMOC since 2004. For 2004–2014, the AMOC at this latitude is calculated from RAPID data and inferences from hydrographic data as 17.6 Sv (Fu et al. 2020) and 16.9 Sv (Worthington et al. 2021). The average of these corresponds to $M=19.4$ Sv (Fig. 16a), close to the CMIP5&6 AOGCM mean of 19.8 Sv. The linear regression relationship between OHUE and AMOC gives $\kappa =0.68$ W m^-2^ K^-1^ for this *M* (Fig. 16b).

### A.2 Global-mean surface temperature change

In Sect. 2 and Appendix C.1﻿, we use $\Delta$SST, the sea-surface temperature difference between the perturbed climate state and piControl, to estimate global-mean surface air temperature change *T*, instead of diagnosing it from the atmosphere GCMs.

*T* and $\Delta$SST are highly correlated across AOGCMs ($r>0.9$, Fig. 16c) at all times in both scenarios, except at the very start of 1pctCO2, and $T\propto {\Delta \textrm{SST}}$ is an excellent approximation. As an example we show the relationship for the $2\times \text{ CO}_{2}$ state (time-mean of years 61–80) in 1pctCO2 (Fig. 16d, $r=0.98$ for 24 AOGCMs). The transient climate response (TCR) is defined as *T* for this state. The ratio $\text{ TCR }/{\Delta \textrm{SST}}$ has a small spread of about 1.4-$-$1.6 (red in Fig. 16d), and $\text{ TCR }=1.5\times {\Delta \textrm{SST}}$ is a very good fit (the line in Fig. 16d).

The best-fit ratio $\text{ TCR }/{\Delta \textrm{SST}}$ varies with time and scenario over a narrow range, again of about 1.4-$-$1.6 (Fig. 16c). For simplicity we take $T=1.5\times {\Delta \textrm{SST}}$ for all times in both scenarios. By using this approximation instead of the diagnosed global-mean surface air temperature change, we eliminate the small model spread in land/sea warming ratio which is irrelevant to our interests.

### A.3 Ocean heat uptake (OHU)

Throughout our analysis, we evaluate ocean heat uptake (OHU, in J) as $H=H_{O}\equiv cV\overline{\theta }$, where $\overline{\theta }$ is the ocean volume-mean temperature change with respect to piControl, *c* is the volumetric heat capacity of sea water, and *V* the volume of the ocean. In Fig. 16e we compare $H_{O}$ with $H_{N}$, the global time-integral of net downward radiation *N* at the top of the atmosphere, for years 61–80 of 1pctCO2. Across AOGCMs these quantities are highly correlated ($r=0.91$). Regression through the origin gives a best-fit $H_{N}/H_{O}=1.07$, consistent with the observational estimate that 93% of heat storage is in the ocean (Rhein et al. 2013), but some models have an exceptionally large ratio (C CNRM-CM5, H GFDL-ESM2M, L IPSL-CM5A-LR, e CNRM-CM6-1, f CNRM-ESM2M-1).

### A.4 Ocean heat uptake efficiency (OHUE)

We can evaluate ocean heat uptake efficiency (OHUE, in W m^-2^ K^-1^) in the $2\times \text{ CO}_{2}$ state of 1pctCO2 (year 70) as

A1 $$\begin{aligned} \kappa _{O} = \left( \frac{1}{{\mathcal {A}}}\,\frac{\textrm{d}H_{O}}{\textrm{d}t}\right) \div {\Delta \textrm{SST}}, \end{aligned}$$

where ${\mathcal {A}}$ is the *global* surface area (not the sea surface area). Thus $\kappa _{O}$ equals the rate of OHU (W per m^2^ of the globe, the first bracket) divided by *T*; this ratio is OHUE by definition. For $\textrm{d}H_{O}/\textrm{d}t$ we use the trend for years 61–80 from linear regression against time. Across models $\kappa _{O}$ is correlated very well ($r=0.86$, Fig. 16f) with OHUE evaluated as the ratio $\kappa _{N}$ of top-of-atmosphere *N* to *T* for time-means of years 61–70. Regression through the origin gives a best-fit $\kappa _{N}/\kappa _{O}=1.03$ i.e. $\kappa _{N}$ is 3% larger. However, a few models give a ratio markedly larger than unity (especially L IPSL-CM5A-LR, e CNRM-CM6-1, f CNRM-ESM2M-1). We suspect that there is a diagnostic problem or non-conservation of energy in these atmosphere models.

For the FAFMIP experiments, the formulae are the same, and we use the final decade, years 61–70. We take $c=4.092\times 10^{6}$ J m^-3^ K^-1^, ${\mathcal {A}}=5.101\times 10^{14}$ m^2^ and $V=1.335\times 10^{18}$ m^3^, assuming the model spread is small in these constants.

## Appendix B Solution of the two-layer model

In this appendix, we derive approximate analytical solutions for the two-layer model,

6 repeated $$\begin{aligned} N = c_{u} \frac{\textrm{d}T}{\textrm{d}t} + \Phi \qquad \text{ where } \qquad \Phi = \gamma (T-T_{d}) = c_{d} \frac{\textrm{d}T_{d}}{\textrm{d}t}, \end{aligned}$$

and other formulae which prove useful for calibrating the *MT*2 model of AOGCMs in Appendix C. In this appendix, *N* denotes the rate of heat uptake by the two-layer model; as a component of the *MT*2 model, the same quantity is denoted *N*_*T*_.

### B.1 Zero-layer solution for steadily increasing forcing

The zero-layer model is a special solution to the two-layer model, obtained by setting the upper-layer heat capacity $c_{u}=0$ and the deep-layer heat capacity $c_{d}=\infty$. Because of the latter, Eq. (6) gives $\textrm{d}T_{d}/\textrm{d}t=0$ for any finite value of the heat flux $\Phi$ from the upper to the deep layer, so $T_{d}=0$ always, and $N=\Phi =\gamma T$. Substituting in Eq. (1) gives $F-\alpha T=\gamma T \Rightarrow T=F/(\alpha +\gamma )$, whence the zero-layer solution (Eq. 2) with $\kappa =\gamma$ (shown in CMIP5 by Gregory et al. 2015). Furthermore, $N \propto F$, since $N \propto T$ and $T \propto F$.

For steadily increasing forcing (like 1pctCO2) $F=Rt$, where *R* is a constant (W m^-2^ yr^-1^), $N \propto F \Rightarrow N \propto t$ i.e. *N*(*t*)/*t* is a constant. Hence the accumulated heat

B2 $$\begin{aligned}{} & {} H(t) = {\mathcal {A}}\int _{0}^{t}\,N(t')\,\textrm{d}t' = {\mathcal {A}} \int _{0}^{t}\,N(t)\frac{t'}{t}\,\textrm{d}t'\nonumber \\{} & {} \quad = {\mathcal {A}} \frac{N(t)}{t} \int _{0}^{t}\,t'\,\textrm{d}t' = \textstyle \frac{1}{2} {\mathcal {A}}Nt \end{aligned}$$

in agreement with Eq. (4). For $t>0$, $H\ne 0$ although $T_{d}=0$ in the zero-layer solution.

We can obtain the zero-layer solution for both steadily increasing forcing and constant forcing in a more rigorous way by first solving the two-layer equations generally and then approximating the solution.

### B.2 General solution

We first substitute for *N* in Eq. (6) from Eq. (1) and rewrite them as

B3 $$\begin{aligned}{} & {} \left( c_{u}\frac{\textrm{d}}{\textrm{d}t} + \alpha + \gamma \right) T - \gamma T_{d} = F \quad \text{(a) } \nonumber \\{} & {} \quad - \gamma T + \left( c_{d}\frac{\textrm{d}}{\textrm{d}t} + \gamma \right) T_{d} = 0. \quad \text{(b) } \end{aligned}$$

Eliminating $T_{d}$, we obtain

B4 $$\begin{aligned} c_{u}c_{d} \frac{\textrm{d}^{2} T}{\textrm{d}t^{2}} + ((\alpha +\gamma )c_{d}+\gamma c_{u})\frac{\textrm{d}T}{\textrm{d}t} + \alpha \gamma T = c_{d}\frac{\textrm{d}F}{\textrm{d}t} + \gamma F, \end{aligned}$$

which is a second-order linear inhomogeneous ordinary differential equation with constant coefficients. The heat capacities $c_{u,d}$, the thermal coupling $\gamma$ and the climate feedback parameter $\alpha$ are all assumed to be constant in time in the two-layer model, although $\alpha$ is certainly not constant in AOGCMs.

Once we have solved this for *T*, we can obtain $T_{d}$ from Eq. (B3) in the form

B5 $$\begin{aligned} \gamma T_{d} = c_{u}\frac{\textrm{d}T}{\textrm{d}t} + (\alpha +\gamma )T - F. \end{aligned}$$

We can also obtain the ocean heat uptake through

B6 $$\begin{aligned}{} & {} H(t) = {\mathcal {A}} \int _{0}^{t} N(t') \, \textrm{d}t' = {\mathcal {A}} \int _{0}^{t} \left( c_{u} \, \frac{\textrm{d}T}{\textrm{d}t} + c_{d} \, \frac{\textrm{d}T_{d}}{\textrm{d}t} \right) \textrm{d}t' \nonumber \\{} & {} \quad = {\mathcal {A}} \, (c_{u} \, T + c_{d} \, T_{d}). \end{aligned}$$

The general solution for the homogeneous case of $F=0$ (the complementary function) is

B7 $$\begin{aligned} T = A_{u}\exp (-t/\tau _{u}) + A_{d}\exp (-t/\tau _{d}), \end{aligned}$$

where $A_{u,d}$ are arbitrary constants, and

B8 $$\begin{aligned}{} & {} \frac{1}{\tau _{u,d}} = \frac{1}{2} \left[ \frac{\alpha +\gamma }{c_{u}} + \frac{\gamma }{c_{d}} \pm \frac{\alpha +\gamma }{c_{u}}\right. \nonumber \\{} & {} \quad \left. \left( 1 - \frac{2\gamma (\alpha -\gamma )}{(\alpha +\gamma )^{2}}\frac{c_{u}}{c_{d}} + \left( \frac{\gamma }{\alpha +\gamma }\frac{c_{u}}{c_{d}}\right) ^{2} \right) ^{\frac{1}{2}} \right] , \end{aligned}$$

gives the two characteristic decay timescales $\tau _{u,d}$. Under scenarios of practical relevance, it is found that $c_{u}\ll c_{d}$ (e.g. Gregory 2000; Geoffroy et al. 2013b). Hence we may approximate by neglecting terms in $c_{u}/c_{d}$ to obtain

B9 $$\begin{aligned} \tau _{u}=\frac{c_{u}}{\alpha +\gamma } \quad \quad \tau _{d}=\frac{(\alpha +\gamma )c_{d}}{\alpha \gamma }, \end{aligned}$$

and their ratio

B10 $$\begin{aligned} \frac{\tau _{u}}{\tau _{d}} = \frac{c_{u}}{c_{d}}\frac{\alpha \gamma }{(\alpha +\gamma )^2} \end{aligned}$$

is of similar size to $c_{u}/c_{d} \Rightarrow \tau _{u}\ll \tau _{d}$.

With $c_{u}\ll c_{d}$, our solutions in the next two sections agree with those of Geoffroy et al. (2013b). Expressed in our notation, their quantities are

B11 $$\begin{aligned} \begin{array}{c} \lambda =\alpha \quad \quad k=R \quad \quad \tau _{f,s}=\tau _{u,d} \\ \displaystyle a_{f}=\frac{\alpha }{\alpha +\gamma } \quad \quad a_{s}=1-a_{f}=\frac{\gamma }{\alpha +\gamma } \quad \quad \tau _{s}a_{s}=\frac{c_{d}}{\alpha } \\ \phi _{s}a_{s}=1 \quad \quad \phi _{f}\ll \phi _{s} \end{array} \end{aligned}$$

### B.3 Solution for steadily increasing forcing

The general solution for $F=Rt$ (as in 1pctCO2) is

B12 $$\begin{aligned}{} & {} T = A_{u}\exp (-t/\tau _{u}) + A_{d}\exp (-t/\tau _{d}) \nonumber \\{} & {} \quad + \frac{Rt}{\alpha } - \frac{R(c_{d}+c_{u})}{\alpha ^{2}}. \end{aligned}$$

Using Eq. (B9) and neglecting terms in $c_{u}/c_{d}$, the solution for $t\gg \tau _{u}$ with $T=T_{d}=0$ at $t=0$ is

B13 $$\begin{aligned}{} & {} T = \frac{Rc_{d}}{\alpha ^{2}}(\exp (-t/\tau _{d})-1) + \frac{Rt}{\alpha } \nonumber \\{} & {} \quad T_{d} = \frac{R\tau _{d}}{\alpha } (\exp (-t/\tau _{d})-1) + \frac{Rt}{\alpha }, \end{aligned}$$

and

B14 $$\begin{aligned} N=F-\alpha T=Rt-\alpha T=\frac{Rc_{d}}{\alpha }(1-\exp (-t/\tau _{d})). \end{aligned}$$

With $\tau _{u}\ll \tau _{d}$, the solutions for *T* and $T_{d}$ (Eq. B13) are the same as given by Eqs. (14) and (15) of Geoffroy et al. (2013b).

Expanding the exponential for $t\ll \tau _{d}$, neglecting $c_{u}$ by comparison with $c_{d}$ and keeping only the first non-zero terms gives

B15 $$\begin{aligned} \begin{array}{c} \displaystyle T=\frac{Rt}{\alpha +\gamma } \quad \quad N = \frac{R\gamma t}{\alpha +\gamma } \quad \quad \kappa =\gamma \\ \displaystyle T_{d}=\frac{Rt^{2}}{2\alpha \tau _{d}} = \frac{Nt}{2c_{d}} \quad \quad H = {\mathcal {A}}c_{d}T_{d} = {\textstyle \frac{1}{2}}{\mathcal {A}}Nt, \end{array} \end{aligned}$$

where $\kappa =N/T$ is the ocean heat uptake efficiency. With $F=Rt$, Eq. (B15) give the zero-layer solution of Eq. (2) and (4).

For $t\gg \tau _{d}$, the exponentials vanish in Eq. (B13), and $T-T_{d}=Rc_{d}/\alpha \gamma$, so the heat flux $\gamma (T-T_{d})$ from the upper to deep layer becomes constant. In this regime, the two temperatures rise at the same rate $R/\alpha$, with the deep layer always cooler. For $t\rightarrow \infty$, $T\rightarrow Rt/\alpha$ and

B16 $$\begin{aligned} N = c_{u}\frac{\textrm{d}T}{\textrm{d}t} + c_{d}\frac{\textrm{d}T_{d}}{\textrm{d}t} \rightarrow (c_{u}+c_{d})\frac{R}{\alpha }, \end{aligned}$$

which is constant, so $\kappa =N/T\rightarrow 0$.

### B.4 Solution for constant forcing

The general solution for constant *F* (as in abrupt4xCO2) is

B17 $$\begin{aligned} T = A_{u}\exp (-t/\tau _{u}) + A_{d}\exp (-t/\tau _{d}) + F/\alpha . \end{aligned}$$

Again neglecting terms in $c_{u}/c_{d}$, the solution with $T=T_{d}=0$ at $t=0$ is

B18 $$\begin{aligned} \begin{array}{c} \displaystyle T = \frac{F}{\alpha }\left( 1 - \frac{\alpha }{\alpha +\gamma }\exp (-t/\tau _{u}) - \frac{\gamma }{\alpha +\gamma }\exp (-t/\tau _{d})\right) \\ \displaystyle T_{d} = \frac{F}{\alpha }(1-\exp (-t/\tau _{d})). \end{array} \end{aligned}$$

The solutions for *T* and $T_{d}$ (Eq. B18) are the same as given by Eqs. (9) and (10) of Geoffroy et al. (2013b). As $t\rightarrow \infty$, both layers approach the equilibrium temperature, $T,T_{d}\rightarrow F/\alpha$.

The term in $\exp (-t/\tau _{u})$ describes the initial rapid warming of the upper ocean, on its short timescale $\tau _{u}$. The rapid warming has been completed once $t\gg \tau _{u}\Rightarrow \exp (-t/\tau _{u})=0$, after which this term can be neglected. Doing so gives

B19 $$\begin{aligned} T=\frac{F}{\alpha }\left( 1-\frac{\gamma }{\alpha +\gamma }\exp (-t/\tau _{d})\right) \end{aligned}$$

and

B20 $$\begin{aligned} N=F-\alpha T=\frac{F\gamma }{\alpha +\gamma }\exp (-t/\tau _{d}), \end{aligned}$$

whence the ocean heat uptake efficiency is

B21 $$\begin{aligned} \kappa= & {} \frac{N}{T} = \frac{F\gamma }{\alpha +\gamma }\exp (-t/\tau _{d})\nonumber \\{} & {} \left[ \frac{F}{\alpha }\left( 1 -\frac{\gamma }{\alpha +\gamma }\exp (-t/\tau _{d})\right) \right] ^{-1} \nonumber \\= & {} \frac{\alpha \gamma }{(\alpha +\gamma )\exp (t/\tau _{d})-\gamma }. \end{aligned}$$

While $t\ll \tau _{d} \Rightarrow \exp (-t/\tau _{d})\simeq 1$, we have $T\simeq F/(\alpha +\gamma )$, $T_{d}\simeq 0$ and $\kappa =\gamma$. At later times which are still small compared with $\tau _{d}$, we can approximate

B22 $$\begin{aligned} T\simeq \frac{F}{\alpha }\left( 1-\frac{\gamma }{\alpha +\gamma } \left( 1-\frac{t}{\tau _{d}}\right) \right) =\frac{F}{\alpha +\gamma }\left( 1+\frac{\gamma t}{\alpha \tau _{d}}\right) \end{aligned}$$

and

B23 $$\begin{aligned} T_{d}\simeq \frac{F}{\alpha } \left( \frac{t}{\tau _{d}}-\frac{t^{2}}{2\tau _{d}^{2}}\right) \simeq \frac{Ft}{\alpha \tau _{d}}. \end{aligned}$$

Hence, the ocean heat uptake per unit of Earth surface area is

$$\begin{aligned} \frac{H}{{\mathcal {A}}}= & \, {} c_{u}T + c_{d}T_{d} \\\simeq & {} \frac{Fc_{u}}{\alpha +\gamma } \left( 1+\frac{\gamma t}{\alpha \tau _{d}}\right) +\frac{Fc_{d}}{\alpha }\frac{\alpha \gamma t}{(\alpha +\gamma )c_{d}} \\\simeq & {} \frac{F}{\alpha +\gamma }\left( c_{u}+\gamma t\right) . \end{aligned}$$

Likewise, the ocean heat uptake per unit of Earth surface area, normalised by the change in global-mean surface temperature, is

B24 $$\begin{aligned} \frac{H}{{\mathcal {A}}T}= & c_{u}+c_{d}\frac{T_{d}}{T}\nonumber \\= & {} c_{u}+c_{d}\frac{1-\exp (-t/\tau _{d})}{1-\gamma /(\alpha +\gamma )\,\exp (-t/\tau _{d})} \end{aligned}$$

B25 $$\begin{aligned}\simeq & {} c_{u}+\gamma t\left( 1-\frac{t}{2\tau _{d}}\right) \left( 1+\frac{\gamma t}{\alpha \tau _{d}}\right) ^{-1} \nonumber \\\simeq & {} c_{u}+\gamma t\left( 1-\frac{t}{\tau _{d}}\left( \frac{\gamma }{\alpha }+\frac{1}{2}\right) \right) \nonumber \\\simeq & {} c_{u}+\gamma t. \end{aligned}$$

For $t\rightarrow \infty$, Eq. (B21) gives $\kappa \rightarrow 0$, whereas at $t=0$, we have $N=F$ and $T=0$ so $\kappa =\infty$. Under constant forcing, the OHUE thus falls from infinity to zero, which is the very opposite of constant!

### B.5 Relationship between *H* and *N* in the two-layer model

For steadily increasing forcing, we have

B26 $$\begin{aligned}{} & {} N = \frac{Rc_{d}}{\alpha }(1-\exp (-t/\tau _{d})) \nonumber \\{} & {} \quad H = \frac{{\mathcal {A}}Rc_{d}}{\alpha }(t-\tau _{d}(1-\exp (-t/\tau _{d})) \end{aligned}$$

(Section B.3, Eqs. B13 and B14). Hence

B27 $$\begin{aligned} \frac{H}{{\mathcal {A}}N} = \frac{t}{1-\exp (-t/\tau _{d})} - \tau _{d}. \end{aligned}$$

Since $\alpha$ is considerably larger than $\gamma$, $\tau _{d}=(\alpha +\gamma )c_{d}/(\alpha \gamma )\simeq c_{d}/\gamma$ (Eq. B9) is AOGCM-neutral if $c_{d}$ and $\gamma$ are, meaning that $H \propto N$ across AOGCMs at any given time. For $t\ll \tau _{d}$

B28 $$\begin{aligned} \frac{H}{{\mathcal {A}}N} \simeq \frac{t}{(t/\tau _{d})-{\textstyle \frac{1}{2}}(t/\tau _{d})^{2}}-\tau _{d} =\frac{{\textstyle \frac{1}{2}}t\tau _{d}}{\tau _{d}-{\textstyle \frac{1}{2}}t}\simeq {\textstyle \frac{1}{2}}t. \end{aligned}$$

Therefore we expect the regression of *H* against ${\mathcal {A}}N$ across AOGCMs to give zero intercept and slope ${\textstyle \frac{1}{2}}t$ in 1pctCO2.

For constant forcing and $t\gg \tau _{u}$ (Sect. B.4, Eqs. B18 and B20), we have

B29 $$\begin{aligned} H \simeq \mathcal{A}c_{d}T_{d} = \frac{{\mathcal {A}}Fc_{d}}{\alpha }(1-\exp (-t/\tau _{d})) \end{aligned}$$

and

B30 $$\begin{aligned}{} & {} N \simeq \frac{F\gamma }{\alpha +\gamma }\exp (-t/\tau _{d}) \nonumber \\{} & {} \quad \Rightarrow \exp (-t/\tau _{d}) = \frac{N(\alpha +\gamma )}{\gamma F}. \end{aligned}$$

Hence

B31 $$\begin{aligned}{} & {} \frac{H}{{\mathcal {A}}} = \frac{Fc_{d}}{\alpha }\left( 1-\frac{N(\alpha +\gamma )}{\gamma F}\right) = \frac{Fc_{d}}{\alpha }\nonumber \\{} & {} \quad - \frac{c_{d}}{\alpha }\frac{\alpha +\gamma }{\gamma }N = \frac{Fc_{d}}{\alpha } - \tau _{d}N, \end{aligned}$$

i.e. *H* varies linearly with *N* over time in a given AOGCM. The intercept of the relationship is AOGCM-specific through $\alpha$, while the slope $\tau _{d}$ is approximately AOGCM-neutral. Furthermore,

B32 $$\begin{aligned} \frac{H}{{\mathcal {A}}N} = \frac{c_{d}(\alpha +\gamma )}{\alpha \gamma } \frac{1-\exp (-t/\tau _{d})}{\exp (-t/\tau _{d})} = \tau _{d}(\exp (t/\tau _{d})-1). \end{aligned}$$

Therefore $H \propto N$ across AOGCMs at a given time, with a slope that increases exponentially with time.

## Appendix C Derivation of the $MT2$ model

In this appendix, we describe the development of the *MT*2 model, in three steps. In the first step (Sect. C.1), guided by the relationships we found among AOGCM quantities for the $2\times \text{ CO}_{2}$ state of the CMIP5&6 1pctCO2 experiment (Sect. 2.12), we introduce a model, called “*MT*”, which contains three parameters (either *U*, *S*, *Q* or *u*, *s*, *q*) that are chosen to fit ocean heat uptake in the AOGCMs. In the second step, we obtain the values of the three parameters of the *MT* model as functions of time in both 1pctCO2 and abrupt4xCO2 scenarios (Sect. C.2). In the third step, we analyse the time- and scenario-dependence of the three parameters, and describe how the form and coefficients of the *MT*2 model are chosen to account for them (Sects. C.3 and C.4), including some consistency checks and corollaries. In Sect. C.5 we derive the relationship between *H* and *N* in the *MT*2 model. Section C.6 describes the “step model”, which is a useful tool in Sects. C.3 and C.4.

### C.1 $MT$ model of OHU in the $2\times \text{ CO}_{2}$ state of 1pctCO2

To account for the points listed in Sect. 2.12, we hypothesise that the variation of OHU *H* across AOGCMs in the $2\times \text{ CO}_{2}$ state of 1pctCO2 depends on *both* the piControl AMOC strength *M* *and* global-mean surface temperature change *T* according to the linear equation

C33 $$\begin{aligned} H = U + S\, (M-\langle M \rangle ) + Q \, T, \end{aligned}$$

where *U*, *S*, *Q* are positive constants, and $\langle M \rangle =19.8$ Sv is the ensemble-mean piControl AMOC strength in the CMIP5&6 AOGCMs of Table 1. (Here and subsequently, $\langle \rangle$ denotes the mean over AOGCMs.) We call Eq. (C33) the “*MT* model”, in reference to its dependence on both *M* and *T*.

The constant *U* comes from point **6**, the term in *S* is introduced to explain point **1** (as shown below, following Eq. C35), and the term in *T* is from point **5**. The choice of $\langle M \rangle$ as a reference AMOC strength is arbitrary. A different reference would change *U* but would not affect *S*. Later on (Sect. C.3) we will make a different choice, suggested by the time-dependence of the *U* and *S* terms. By multiple linear regression of *H* against *M* and *T* according to Eq. (C33) we determine that $U=224\pm 86$ ZJ, $S=13.0\pm 3.1$ ZJ Sv^-1^ and $Q=456\pm 64$ ZJ K^-1^ with $r=0.83$ (at the time of $2\times \text{ CO}_{2}$ in 1pctCO2). We use $T=1.5\,{\Delta \textrm{SST}}$, as in Sect. 2.

In view of $H\propto N$ (point **4**), the *MT* model implies that the rate of OHU

C34 $$\begin{aligned} N = u + s \,(M-\langle M \rangle ) + q \, T, \end{aligned}$$

where *u*, *s*, *q* are constants. Correspondingly, by multiple linear regression of *N* against *M* and *T* according to Eq. (C34), we obtain $u=0.53\pm 0.16$ W m^-2^, $s=0.023\pm 0.006$ W m^-2^ Sv^-1^, $q=0.40\pm 0.12$ W m^-2^ K^-1^ with $r=0.72$.

Alternatively we can calculate *u*, *s*, *q* by dividing *U*, *S*, *Q* by *H*/*N*. We evaluate this ratio for year 70 of 1pctCO2 as the ordinary least-squares regression slope of *H* against *N* (Fig. 4b). We obtain $u=0.33$ W m^-2^, $s=0.019$ W m^-2^ Sv^-1^, $q=0.67$ W m^-2^ K^-1^, consistent within standard errors of the coefficients from multiple regression for *N*. We prefer the values from the multiple regression for *H* because its correlation is larger and the uncertainties of the coefficients are smaller, both owing to the smaller unforced variability in *H* than *N*.

The multiple regression for *H* against both *M* and *T* has a higher correlation ($r=0.83$) than *H* with *T* alone ($r=0.71$), because *M* explains some of the *H* variance. The sensitivity $Q=\partial H/\partial T$ in the multiple regression (the slope of the dashed line in Fig. 4a) is larger than $\textrm{d}H/\textrm{d}T$ from regression of *H* against *T* alone (the slope of the solid line), because *M* and *T* are anticorrelated (point **3**, Fig. 2e). Hence, AOGCMs with larger *T* have smaller *H* than would be expected if the effect of *M* were ignored (as it is by the regression against *T* alone). Consequently, the constant *U* of the multiple regression is less than the *H*-intercept from the regression against *T* alone (compare the *H*-intercepts of the solid and dashed lines in Fig. 4a).

According to Eq. (C33), AOGCMs in which $M=\langle M \rangle$ have $H=U+QT$ (the dashed line in Fig. 4a). AOGCMs with $M>\langle M \rangle$ (stronger AMOC than the CMIP5&6 AOGCM mean of 19.8 Sv) tend to lie above the regression line of *H* against *T* (Fig. 4a, those above the solid line have mean *M* equal to 22.4 Sv), and AOGCMs with $M<\langle M \rangle$ tend to lie below (with mean *M* equal to 16.6 Sv). Thus, *H* tends to be larger with greater *M* at a given *T*, because $S=\partial H/\partial M>0$. Similarly, *H* tends to be larger with greater *T* at a given *M*, because $Q=\partial H/\partial T>0$. These two tendencies can be seen simultaneously by considering the two-dimensional function *H*(*M*, *T*) (Eq. C33, Fig. 12; the figure is for the *MT*2 model, not the *MT* model, but they are very similar). Since $N\propto H$, *N* has likewise a small spread across AOGCMs.

In terms of the *MT* model, the small spread of *H* is a consequence of the postulate that it increases with both *M* and *T* (Eqs. C33 and C34), in combination with the anticorrelation of *M* and *T* across AOGCMs. The small spread of *H* accounts for the lack of correlation between *H* and *M* (point **2**), and hence between *H* and OHUE too (point **2**), given that *M* and OHUE are strongly correlated (point **1**).

From Eq. (C34), the *MT* model gives the OHUE as

C35 $$\begin{aligned} \kappa = \frac{N}{T} = \frac{u}{T}+s\frac{M-\langle M \rangle }{T} + q, \end{aligned}$$

in which the thermal coupling *q* is the same for all AOGCMs. The *s* term gives a correlation between $\kappa$ and *M* (point **1**). The correlation is strengthened by the anticorrelation of *M* and *T* i.e. *T* is smaller when *M* is larger (point **3**). Thus this same anticorrelation accounts both for the strong relationship between OHUE and *M*, and for the lack of relationship between OHU and *M* (previous paragraph).

### C.2 Time- and scenario-dependent $MT$ model

In the *MT* model of Sect. C.1 we consider only one state in one forcing scenario (year 70 in 1pctCO2). We need to know whether the *MT* model is generally applicable to time-dependent scenarios. Therefore, as a second step towards the new conceptual model, we next consider abrupt4xCO2 as well as 1pctCO2, and in each scenario we fit the *MT* model as a function of time. The advantage of abrupt4xCO2 is that the forcing has no intrinsic timescale, so all temporal correlations arise from the response of the system, whereas many temporal correlations may arise in 1pctCO2 because the continually changing forcing affects all quantities simultaneously. We use results from 29 CMIP5&6 AOGCMs for 1pctCO2 and 23 for abrupt4xCO2 (Table 1).

In both scenarios, we regress OHU *H* against *T* and piControl AMOC strength *M* to obtain *U*, *S*, *Q* according to Eq. (C33), using overlapping 20-year means at 10-year intervals (covering years 1–20, 11–30, 21–40, ..., 121–140, and applying nominally to years 10, 20, ..., 130). Whereas in Sects. 2 and C.1 we estimated *T* from $\Delta$SST, now we adopt global-mean surface air temperature change for *T*, in order to be consistent with general practice for simple models of the Earth energy balance and the definition of TCR. However, we continue to use ocean temperature to evaluate *N* (Sect 2.1).

At all times under both scenarios, we find that the rate of OHU $N \propto H$ (solid red and black lines in Fig. 18c show high correlation, dash-dotted lines show small intercept from regression of *H* against *N*), as in the $2\times \text{ CO}_{2}$ state of 1pctCO2. (In Appendix C.5 we present evidence and explanations for the high correlation of *H* and *N*, and the time- and scenario-dependence of their ratio.) Given that $N\propto H$, we expect Eq. (C34) to be applicable as well as Eq. (C33), so we also regress *N* against *M* and *T* at each time in each scenario to obtain *u*, *s*, *q*. Thus we obtain time- and scenario-dependent coefficients *U*, *S*, *Q*, *u*, *s*, *q* for the state-dependent *MT* model (Fig. 17).

The multiple correlation coefficient for *H* with *M* and *T* is about 0.8 at all times in abrupt4xCO2, while in 1pctCO2 it starts from a small value (because the forced response is initially small compared with unforced variability) and increases, passing 0.8 at year 60 (solid lines in Fig. 17b). The high correlation coefficient indicates that the *MT* model is a good fit (except for the first 20 years of 1pctCO2, which we omit because the forced response is obscured). The multiple correlation coefficient for *N* (dotted lines in Fig. 17b) has a similar time-profile to that for *H*, but is smaller at all times in both scenarios (because of the greater effect of unforced variability on *N*).

### C.3 Forcing-dependent rate of heat uptake

#### C.3.1 AMOC-dependent term

The AMOC-dependent term in the *MT* model for *H* is $S(t)M'$ (Eq. C33), where $M'\equiv M-\langle M \rangle$. Its AOGCM-neutral coefficient $\partial H/\partial M=S(t)$ in abrupt4xCO2 is proportional to time (solid red line in Fig. 17e). Linear regression of *S*(*t*) against time (dotted red line) gives a slope $\dot{S}\equiv \textrm{d}S/\textrm{d}t=0.57\pm 0.01$ ZJ yr^-1^ Sv^-1^ and negligible intercept. The *M* term in the *MT* model for *N* in abrupt4xCO2 is $s(t)M'$ (Eq. C34), whose coefficient *s* (solid red line in Fig. 17f) has only a small trend (dotted red line). Its time-mean (dashed red line) is $s=0.037$ W m^-2^ Sv^-1^.

Treating *s* as constant, the contribution $s M'$ to *N* makes a time-integral contribution to *H* of ${\mathcal {A}}\int \,s M'\,\textrm{d}t= {\mathcal {A}}s M'\,t$, which we can identify with the *M* term in *H* i.e. $SM'=\dot{S}M'\,t\equiv {\mathcal {A}}s M't \Rightarrow \dot{S}={\mathcal {A}}s$. Hence $\dot{S}$ from the *MT* fit for *H* gives $s=\dot{S}/{\mathcal {A}}=0.0351\pm 0.0007$ W m^-2^ Sv^-1^, very close to *s* from the *MT* fit for *N*. The fits for *H* and *N* in abrupt4xCO2 thus consistently describe a flux of heat which accumulates in the ocean at a constant rate ${\mathcal {A}}s M'$ determined partly by AOGCM-neutral time-constant factors, quantified by *s*, including the radiative forcing, and partly by AOGCM-specific time-constant factors, quantified by $M'$.

By substituting $\dot{S}t$ from abrupt4xCO2 (dotted red line in Fig. 17e) for $X_{4}(t)$ in the step model (Eq. C59, Appendix C.6), we may estimate *S*(*t*) in 1pctCO2 as $X_{1}(t)$. Because *S* increases linearly in time in abrupt4xCO2, the step model predicts that it should increase quadratically in time in 1pctCO2 (dashed black line in Fig. 17e). Similarly, assuming constant *s* in abrupt4xCO2 predicts a linearly increasing $s \propto t$ in 1pctCO2 (dashed black line in Fig. 17f). The step-model estimates of *S* and *s* in the second half of 1pctCO2 are somewhat smaller than the *MT* fit, but not significantly so (comparing the dashed black lines with the grey envelopes in Fig. 17e, f). Therefore the *M* term describes a flux of heat which accumulates in the ocean at an increasing rate in 1pctCO2.

The successful application of the step model to estimate 1pctCO2 from abrupt4xCO2 is evidence that the *M* term depends linearly on forcing. We therefore postulate that $s(t)=s_{0}F(t)$, where $s_{0}$ is a constant that does not depend on AOGCM, time or forcing. This would account for constant *s* in abrupt4xCO2 and $s\propto t$ in 1pctCO2. Taking $F_{4\times }=7.5$ W m^-2^ for abrupt4xCO2 we obtain $s_{0}=s/F_{4\times }=\dot{S}/({\mathcal {A}}F_{4\times })=0.0047\pm 0.0001$ Sv^-1^. That is, about 0.5% of the forcing is taken up passively by this term, per Sv of the deviation of *M* from its AOGCM-mean value $\langle M \rangle$.

Writing $N_{M}(t)=s_{0}F(t)M'$ for the contribution from this term to the rate of OHU at time *t*, its contribution to the OHU accumulated by time *t* is

C36 $$\begin{aligned} H_{M}(t)\equiv & {} {\mathcal {A}}\int _{0}^{t}\,N_{M}(t')\,\textrm{d}t' ={\mathcal {A}}\int _{0}^{t}\,s_{0}F(t')M'\,\textrm{d}t'\nonumber \\= & {} {\mathcal {A}}s_{0}M'\int _{0}^{t}\,F(t')\,\textrm{d}t' \\ \nonumber= & {} {\mathcal {A}}s_{0}M'\left\{ \begin{array}{ll} {\textstyle \frac{1}{2}}Rt^{2}={\textstyle \frac{1}{2}}Ft \\ F_{4\times }t \end{array}\right. ={\mathcal {A}}N_{M} \left\{ \begin{array}{ll} {\textstyle \frac{1}{2}}t &{} \text{1pctCO2 } \\ t &{} \text{ abrupt4xCO2, } \end{array}\right. \end{aligned}$$

since $F=F_{4\times }$ in abrupt4xCO2 and $F=Rt$ in 1pctCO2 with constant $R=0.05$ W m^-2^ yr^-1^ ($F_{4\times }$ divided by 140 years, the time taken for quadrupling at 1% yr^-1^). Hence

C37 $$\begin{aligned} \frac{H_{M}}{{\mathcal {A}}N_{M}}=\left\{ \begin{array}{ll} {\textstyle \frac{1}{2}}t &{} \text{1pctCO2 } \\ t &{} \text{ abrupt4xCO2. } \end{array}\right. \end{aligned}$$

In the next Sect. (C.3.2) we redefine $M'$, but the above formulae continue to apply, with the addition of a small AOGCM-neutral constant term to $H_{M}$ (*cf.* Eq. C40).

#### C.3.2 AOGCM-neutral term

The AOGCM-neutral term *U*(*t*) in the fit for *H* (Eq. C33) is linearly dependent on time in abrupt4xCO2 (solid red line in Fig. 17c). Its linear regression against time (dotted red line in Fig. 17c, which coincides with the turquoise line) has a small intercept $U_{0}=84$ ZJ and slope $\dot{U}\equiv \textrm{d}U/\textrm{d}t=16.9\pm 0.5$ ZJ yr^-1^. The corresponding contribution to *u* is nearly constant (solid and dotted red lines in Fig. 17d); its time-mean (dashed red line) is 1.0 W m^-2^. Thus, *U* and *u* consistently describe OHU at a rate which is constant in time (like the *M* term) and AOGCM-neutral (unlike the *M* term), with $\textrm{d}U/\textrm{d}t=\dot{U}={\mathcal {A}}u$ (in which $U_{0}$ vanishes). The sum of contributions from this term and the *M* term is $U(t)+S(t)M'=U_{0}+(\dot{U}+\dot{S}M')t$ to *H* and $u+s M'$ to *N*.

In formulating the *MT* model, we chose $M'=M-\langle M \rangle$ as an independent variable. We could equally well have chosen any value $M_{0}$ instead of $\langle M \rangle$ as the reference for the unperturbed AMOC strength. For any AOGCM, introducing an arbitrary constant $M_{0}$,

C38 $$\begin{aligned}{} & {} \dot{U}+\dot{S}M'= \dot{U}+\dot{S}(M-\langle M \rangle ) \nonumber \\{} & {} \quad =\dot{U}+\dot{S}(M-M_{0}+M_{0}-\langle M \rangle ) \nonumber \\{} & {} \quad =(\dot{U}+\dot{S}M_{0}-\dot{S}\langle M \rangle )+\dot{S}(M-M_{0}) \nonumber \\{} & {} \quad \Rightarrow \dot{U}t + \dot{S}M'\,t = (\dot{U}+\dot{S}M_{0}-\dot{S}\langle M \rangle )t+\dot{S}(M-M_{0})t. \end{aligned}$$

Hence we can write the sum of these two time-dependent terms as a single time-dependent term

C39 $$\begin{aligned} \dot{S}M'\,t=\dot{S}(M-M_{0})t \end{aligned}$$

if we choose $M_{0}$ such that $\dot{U}+\dot{S}M_{0}-\dot{S}\langle M \rangle =0$. Although this is an arbitrary choice which does not in itself give any physical insight, we prefer it for simplicity.

In our set of abrupt4xCO2 AOGCMs, $\langle M \rangle =19.8$ Sv, whence $M_{0}=\langle M \rangle -\dot{U}/\dot{S}=-10.2$ Sv. Henceforth we redefine $M'\equiv M-M_{0}$. With this choice of $M_{0}$, an AOGCM with $M=\langle M \rangle$ has $H_{M}=U_{0}+S(\langle M \rangle -M_{0})=U_{0}+{\mathcal {A}}s_{0}(\langle M \rangle -M_{0})F_{4\times }t$ (turquoise line in Fig. 17c), which is very close to *U*(*t*) (red line), as should be so by construction.

Likewise we replace $u+s(M-\langle M \rangle )$ with $s(M-M_{0})$. Our values for constant *s* and constant $M_{0}$ predict constant $s(\langle M \rangle -M_{0})=s_{0}(\langle M \rangle -M_{0})F_{4\times }$ (turquoise line in Fig. 17d) exceeding time-mean *u* by $\sim$0.05 W m^-2^. This error is only a small fraction of *N*.

By redefining $M'\equiv M-M_{0}$, we include *U* in $H_{M}$ and *u* in $N_{M}$, thus

C40 $$\begin{aligned} H_{M} = U_{0} + {\mathcal {A}}s_{0}M'\,F_{4\times }t \quad \quad N_{M}=\frac{1}{{\mathcal {A}}}\,\frac{\textrm{d}H_{M}}{\textrm{d}t}=s_{0}M'\,F_{4\times } \end{aligned}$$

for abrupt4xCO2. These formulae cannot be accurate for the first few years, since we must have $H_{M}=0$ at $t=0$, implying that $U_{0}$ takes some time to build up.

Applying the step model to *U* and *u* from abrupt4xCO2 gives predictions for 1pctCO2 that increase quadratically and linearly in time respectively (black dashed lines in Fig. 17c,d), as for *S* and *s*, but these are significantly larger than the *MT* fit in later years. We believe that the step model is more accurate for 1pctCO2, and that the *MT* fit underestimates *U* and *u*. This might happen because the predictors *T* and $M'$ in the multilinear regressions for *H* or *N* are significantly anticorrelated (dashed lines in Fig. 17b), so the corresponding slopes *Q* or *q* and *S* or *s* will tend to err in the same direction. We noted above that *S* and *s* are slightly overestimated for 1pctCO2 (compared with the step-model projections). If both the slopes are simultaneously overestimated by the regression, the constants *U* and *u* will consequently be underestimated by the *MT* fit. This argument applies to abrupt4xCO2 as well as to 1pctCO2, but we prefer to rely on abrupt4xCO2 because the time-dependence of its *U*(*t*) and *u*(*t*) can be simply and satisfactorily explained ($U=\dot{S}M'\,t$ and $u=s M'$ as above). Therefore we use Eq. (C40) for $N_{M}$ (Eq. 5) and $H_{M}$ (Eq. 7) in the *MT*2 model.

### C.4 Temperature-dependent rate of heat uptake

#### C.4.1 The form of the model

We estimate the temperature-dependent part of OHU *H*(*t*) for each AOGCM as $H_{T}(t)=H(t)-H_{M}(t)$, as derived in Sect. C.3, where *H* is diagnosed from AOGCM ocean temperature and $H_{M}$ from Eq. (C40), using *M* for the AOGCM. Correspondingly, we estimate $N_{T}(t)=N(t)-N_{M}(t)$. There are small negative values for $H_{T}$ for the first five years of abrupt4xCO2, which is physically unexpected. It probably arises from the inaccuracy of Eq. (C40) in the early years, and we ignore those years for the purpose of calibrating a model for $H_{T}$. We note that AOGCM-mean $H_{T}$ and $H_{M}$ are about the same size (Fig. 11a).

In the *MT*2 model, the temperature-dependent part of *H* takes the form $H_{T}=Q(t) T(t)$ (Eq. C33). The AOGCM-neutral coefficient $\partial H/\partial T=Q(t)$ increases non-linearly with time in abrupt4xCO2 (red line in Fig. 17g, convex upward) and extrapolation suggests that $Q>0$ at $t=0$. This is clearer for 1pctCO2 (black line), which has less curvature. The zero-layer model for 1pctCO2 gives $H_{T}=\frac{1}{2}{\mathcal {A}}tN_{T}=\frac{1}{2}{\mathcal {A}}t\gamma T \Rightarrow Q=H_{T}/T=\frac{1}{2}{\mathcal {A}}\gamma t$ (Eqs. 2 and 4), which incorrectly predicts that $Q=0$ at $t=0$. Thus the zero-layer model is not a good description of the *T*-dependent rate of OHU.

The zero-layer model assumes $q=N_{T}/T=\gamma$ to be constant, but actually it is a declining function of time in both scenarios (red and black lines in Fig. 17h). Again, the zero-layer model does not correctly describe the AOGCMs.

An obvious hypothesis is that $H_{T}(t)$ and $N_{T}(t)$ might follow the two-layer model (Eq. 6), without making the zero-layer approximation. We find that the two-layer model with AOGCM-neutral parameters is indeed a good fit for $H_{T}(t)$ and $N_{T}(t)$ from each AOGCM, given its *T*(*t*), as we show in the following subsections. This is the two-layer component of the *MT*2 model, which we adopt in Eq. (5) and Eq. (7). The *MT*2*T* variant has the same form, but with AOGCM-specific parameters.

#### C.4.2 Fitting the two-layer model for $H_{T}(T)$

In this subsection we write $C_{u,d}$ for ${\mathcal {A}}c_{u,d}$, for convenience. From Eq. (6) we obtain

C41 $$\begin{aligned} H_{T} = \int _{0}^{t} N\,\textrm{d}t' = C_{u} T + C_{d} T_{d} \end{aligned}$$

and

C42 $$\begin{aligned} C_{d}T_{d} = \int _{0}^{t} \gamma (T-T_{d})\,\textrm{d}t' = \gamma \int _{0}^{t} T\,\textrm{d}t' - \frac{\gamma }{C_{d}}\int _{0}^{t} (H_{T}-C_{u}T)\,\textrm{d}t' \end{aligned}$$

so

C43 $$\begin{aligned} H_{T} - C_{u}T = \gamma \int _{0}^{t} T\,\textrm{d}t' - \frac{\gamma }{C_{d}} \int _{0}^{t} H_{T}\,\textrm{d}t' + \frac{\gamma C_{u}}{C_{d}} \int _{0}^{t} T\,\textrm{d}t' \end{aligned}$$

i.e.

C44 $$\begin{aligned} H_{T} = C_{u}T + \left( \gamma +\frac{C_{u}}{C_{d}}\right) \int _{0}^{t} T\,\textrm{d}t' - \frac{\gamma }{C_{d}} \int _{0}^{t} H_{T}\,\textrm{d}t'. \end{aligned}$$

We estimate the two-layer model parameters $\gamma$, $C_{u}$ and $C_{d}$ to fit $H_{T}(t)$, given *T*(*t*) for abrupt4xCO2, by multiple linear regression of $H_{T}$ against *T*, $\int T\,\textrm{d}t$ and $\int H_{T}\,\textrm{d}t$, requiring the fit to pass through the origin, when all quantities are zero. This yields three coefficients

C45 $$\begin{aligned} a_{1} = C_{u} \quad \quad a_{2}=\gamma +\frac{C_{u}}{C_{d}} \quad \quad a_{3}=-\frac{\gamma }{C_{d}} \end{aligned}$$

whence

C46 $$\begin{aligned} C_{u} = a_{1} \quad \quad C_{d} = -\frac{a_{2}}{a_{3}}-a_{1} \quad \quad \gamma = a_{2} + a_{1}a_{3}. \end{aligned}$$

For *MT*2, we carry out this procedure using AOGCM-mean $H_{T}(t)$ and *T*(*t*). The fit is excellent (Fig 17a; the multiple correlation coefficient exceeds 0.999). We obtain $C_{u}=60\pm 2$ ZJ K^-1^ ($c_{u}=3.7$ $\mathrm{W\,yr\,m}^{-2}\,\textrm{K}^{-1}$), $C_{d}=454\pm 14$ ZJ K^-1^ ($c_{d}=28.2$ $\mathrm{W\,yr\,m}^{-2}\,\textrm{K}^{-1}$) and $\gamma =0.470\pm 0.008$ W m^-2^ K^-1^. For *MT*2*T*, we fit $H_{T}(t)$ and *T*(*t*) from each AOGCM individually.

Geoffroy et al. (2013b) calibrated the two-layer model for a set of CMIP5 AOGCMs in abrupt4xCO2. For the seven of their CMIP5 AOGCMs also used in our analysis, the mean *T*(*t*) of their calibrated two-layer model agrees well with the AOGCMs (compare solid and dashed green lines in Fig. 10a), except that initial warming of the two-layer model is too rapid (as can also be seen in several panels of their Figure 2). However, their two-layer model *H*(*t*) is an overestimate of the AOGCM mean by about 10% (compare solid and dashed green lines in Fig. 11a). The *MT*2 model *H*(*t*) (given the same *T*(*t*) as input) matches the AOGCM mean better (compare solid green and dotted blue lines).

During the early years of abrupt4xCO2, practically all of *H* is stored in the upper ocean. Within the *MT*2 model, the two-layer component accounts for about half ($H_{T}$) of the OHU *H*, (the other half is $H_{M}$, which is not *T*-dependent), whereas Geoffroy et al. (2013b) fit the two-layer model to the entire *H*. Hence the *MT*2 two-layer model requires only about half the upper-ocean heat capacity in the initial phase, since *T* is similar. Thus *MT*2 $c_{u}$ is about half the size of $c_{u}=7.3\pm 1.1$ $\mathrm{W\,yr\,m}^{-2}\,\textrm{K}^{-1}$ for the mean of CMIP5 AOGCMs considered by Geoffroy et al. (2013b). Similarly, the *MT*2 $\gamma$ is only about two-thirds of their $\gamma =0.74\pm 0.18$ W m^-2^ K^-1^, because the heat flux $\gamma (T-T_{d})$ is about half the size, being mainly controlled by *T* for the next several decades while $T_{d}$ is still small.

The *MT*2 $c_{d}$ is about a third of the $c_{d}=91\pm 27$ $\mathrm{W\,yr\,m}^{-2}\,\textrm{K}^{-1}$ of Geoffroy et al. (2013b). With two-thirds of the heat flux into the deep ocean, but only a third of the heat capacity, our $T_{d}$ warms faster and approaches equilibrium more quickly. The *MT*2 deep-ocean timescale $\tau _{d}\simeq 80$ years, about a third of theirs.

The *MT*2 coefficients can also be estimated by the above procedure using AOGCM-mean 1pctCO2 results. Using these coefficients to estimate $H_{MT}$ from *T* for individual AOGCMs in 1pctCO2 gives similar values to those obtained with the same *T* and the *MT*2 coefficients from the abrupt4xCO2 fit. They have similarly high correlation with AOGCM *H* ($r=0.82$) and similar RMS error (177 ZJ). The similarity confirms that the two-layer fit is scenario-independent. The values are all slightly larger, which reduces the mean bias to $-10$ ZJ.

Despite this small improvement, we think the abrupt4xCO2 coefficients are more reliable, for the following reasons. The *MT*2 1pctCO2 fit gives similar $C_{d}=461\pm 8$ ZJ K^-1^ to abrupt4xCO2, but larger $\gamma =0.679\pm 0.008$ W m^-2^ K^-1^ and smaller $C_{u}=20\pm 1$ ZJ K^-1^, equivalent to only 10 m thickness of water, which seems implausibly small. We think $\gamma$ is less well-constrained than by abrupt4xCO2, because the two large terms ($\int T\,\textrm{d}t$ and $\int H_{T}\,\textrm{d}t$) are opposed in sign and both proportional to $\gamma$ but have similar time-profiles, and are thus degenerate. This uncertainty is not indicated by the errors from the AOGCM-mean *MT*2 fits, but if the two-layer fit is carried out for individual AOGCMs, rather than the AOGCM mean (Sect. 3.4), all three parameters have a much larger standard deviation (across AOGCMs) for 1pctCO2 than for abrupt4xCO2. In particular, for individual AOGCMs $\gamma =0.470\pm 0.068$ W m^-2^ K^-1^ in abrupt4xCO2, $0.667\pm 0.300$ W m^-2^ K^-1^ in 1pctCO2. We therefore prefer the abrupt4xCO2 calibration.

#### C.4.3 Relationships of $N_{T}$ and $H_{T}$ for abrupt4xCO2

In this section, we show that the *MT*2 two-layer model reproduces and explains the relationships of AOGCM $H_{T}$ and $N_{T}$ with each other and with *T* for abrupt4xCO2.

We calculate *MT*2 $N_{T}(t)$ for abrupt4xCO2 in each AOGCM with the two-layer model using its own *T*(*t*) and the AOGCM-neutral $c_{u},c_{d},\gamma$ from the multiple linear regression of Eq. (C44). At each *t*, we regress $N_{T}$ against *T* across AOGCMs. The slope from the regression varies over time, and is close to *q*(*t*) (dotted orange line is close to solid red in Fig. 17h). This agreement provides evidence that $N_{T}$ simulated by the two-layer model for each AOGCM is consistent with $N_{T}=qT$ inferred from the *MT* fit across AOGCMs. We also simulate $N_{T}(t)$ for the AOGCM-mean *T*(*t*) with the *MT*2 two-layer model, and plot $N_{T}/T$ (solid orange line in Fig. 17h), which is likewise close to *q* (solid red line). From the two-layer model solution, we expect that $q\simeq \gamma$ when $\tau _{u}\ll t \ll \tau _{d}$ (as shown in the sentence following Eq. B21). This point is reached at about 20 years (when the red line crosses the horizontal dotted black line).

The climate feedback parameter $\alpha$ is AOGCM-specific ($1.22\pm 0.35$ W m^-2^ K^-1^ for years 1–20, Table 1) but in most cases considerably larger than the *MT*2 $\gamma =0.470$ W m^-2^ K^-1^ (Sect. C.4.2). Neglecting $\gamma$ in comparison with $\alpha$, the two-layer model gives $\tau _{d}\simeq c_{d}/\gamma$ (Eq. B9) and

C47 $$\begin{aligned} \frac{N_{T}}{T} = q_{0}^{*}\equiv \gamma \exp (-t/\tau _{d}) \end{aligned}$$

for $t\gg \tau _{u}$ under constant forcing (Eq. B21). The ratio $q_{0}^{*}(t)$ should be approximately the same as *q*(*t*) of Eq. (C34), the coefficient of *T* from the multiple linear regression of *N*(*M*, *T*) in CMIP5&6 AOGCM abrupt4xCO2 results (red line in Fig. 17h). We find that it has the same time-profile as *q*(*t*) but makes a small underestimate (dotted green line compared with red line in Fig. 17h). This may be because neglecting $\gamma$ necessarily underestimates $\tau _{d}$, so the exponential decline is too fast. In the early decades, the underestimate is larger, perhaps because of the omission of the storage of heat in the upper ocean (no $c_{u}$ term in Eq. C47).

A feature of Eq. (C47) is that $q_{0}^{*}$ is AOGCM-neutral if $\gamma$ and $c_{d}$ are. This explains $N_{T}(t)\propto T(t)$ across AOGCMs for a given *t* in abrupt4xCO2 (Fig. 14). We find that $q_{0}^{*}=0.37,0.11$ W m^-2^ K^-1^ at $t=20,120$ yr from Eq. (C47) (green lines in Fig. 14) is indeed close to the regression slope of $N_{T}$ against *T* for the AOGCM results (orange and red lines). (We commented in the last paragraph on the underestimate in early decades.)

By integrating $N_{T}(t)$ for each AOGCM, we obtain the corresponding *MT*2 estimates of $H_{T}(t)$. The regression slope across AOGCMs at each *t* of *MT*2 $H_{T}$ against *T*(*t*) gives a good estimate of *Q*(*t*) from the *MT* fit (dotted orange line is close to solid red line in Fig. 17g).

The two-layer model predicts a quadratic dependence on time for $H_{T}/T={\mathcal {A}}(c_{u}+\gamma t -(\gamma /\alpha +{\textstyle \frac{1}{2}})\gamma t^{2}/\tau _{d})$ (Eq. B25) while $\tau _{u}\ll t\ll \tau _{d}$ under constant forcing. If the curve of $Q=H_T/T$ in abrupt4xCO2 from the *MT* fit (red line in Fig. 17g) could be described by a quadratic, the two-layer formula could be used to estimate $c_{u}$ and $\gamma$ if $\alpha$ were known; possibly even a linear fit $Q=H_{T}/T={\mathcal {A}}(c_{u}+\gamma t)$, neglecting the $t^{2}$ term, might be adequate for obtaining $c_{u}$ and $\gamma$ without knowledge of $\alpha$. Actually this appearance is misleading. The quadratic approximation of $\exp (-t/\tau _{d})$ is practically useless (green line) with our small $\tau _{d}$. With the two-layer parameters of Geoffroy et al. (2013b), the quadratic approximation is reasonable for $t\lesssim 50$ years, and it is quite accurate for 150 years if $c_{d}$ is four times larger than in Geoffroy et al. (2013b)’s model, for example (not shown).

At a given time in abrupt4xCO2, *MT*2 $H_{T}$ and $N_{T}$ are highly correlated across AOGCMs (solid orange line in Fig. 18c). The relationship of $N_{T}$ to $H_{T}$ is shown for three different times as examples in Fig. 18d (orange crosses, with dashed orange regression lines). The slope of linear regression of $H_{T}$ against $N_{T}$ increases in time (dashed orange line in Fig. 18c) at a fairly steady rate (the dashed orange line is well-fitted by the constant trend indicated by the dotted orange line), while the intercept is relatively small (rising in later decades, dash-dotted orange line). We also see small intercepts increasing in time in Fig. 18d, with slopes *decreasing* in time because the regression is transposed i.e. $N_{T}$ against $H_{T}$. The *MT*2 behaviour agrees fairly well with the time-dependent linear relationship of $H_{T}$ and $N_{T}$ predicted by the two-layer model

B31 repeated $$\begin{aligned} \frac{H_{T}}{{\mathcal {A}}} = \frac{Fc_{d}}{\alpha } - \tau _{d}N_{T} \qquad \text{ for } \text{ a } \text{ given } \text{ AOGCM } \text{ as } \text{ a } \text{ function } \text{ of } \text{ time } \end{aligned}$$

and

B32 repeated $$\begin{aligned} \frac{H_{T}}{{\mathcal {A}}N_{T}} = \tau _{d}(\exp (t/\tau _{d})-1) \qquad \text{ across } \text{ AOGCMs } \text{ at } \text{ a } \text{ given } \text{ time } \end{aligned}$$

with various degrees of approximation, as follows:

$\bullet$ If we assume $\gamma \ll \alpha$ and that $c_{d}$ and $\gamma$ are AOGCM-neutral, the deep-layer timescale $\tau _{d}$ is also AOGCM-neutral (as in Eq. C47). Hence trajectories of $(N_{T}(t),H_{T}(t))$ for different AOGCMs are parallel but with different $H_{T}$-intercepts dependent on their $\alpha$ (Eq. B31, examples for three values of $\alpha$ are shown as solid green lines in Fig. 18d, where we have assigned $\tau _{d}$ its value from the *MT*2 fit). Furthermore, in that case, $H_{T}\propto N_{T}$ across AOGCMs at any given time, and the constant of proportionality increases exponentially with time (Eq. B32, examples for three times are shown as dashed green lines in Fig 18d).

$\bullet$ In the two-layer model, $\tau _{d}$ is larger for smaller $\alpha$, if $\gamma$ and $c_{d}$ are independent of $\alpha$ (Eq. B9). Including this effect makes the trajectory steeper for smaller $\alpha$ (Eq. B31, solid black lines in Fig. 18d). The relationship of $H_{T}$ and $N_{T}$ at a given time remains roughly linear, as in Eq. (B32), despite $\tau _{d}$ not being constant, but it no longer passes through the origin (dashed black lines), and the intercept increases with time.

$\bullet$ For AOGCMs, the $(N_{T}(t),H_{T}(t))$ trajectories are not straight lines, because $\alpha$ decreases with time in an AOGCM-specific way, making the slope steeper as $H_{T}$ increases (thin orange lines in Fig. 18d). Furthermore, the best choice of $c_{d}$ is AOGCM-specific (although the *MT*2 fit is reasonably good for all, Sect. 3.4). For both these reasons, the relationship of $H_{T}$ and $N_{T}$ at a given time in AOGCMs has considerable scatter, but a linear fit is still fairly good (dashed orange lines in Fig. 18d). As in the previous case (two-layer model with variable $\tau _{d}$), the intercept increases with time (dash-dotted orange line in Fig. 18c). The slope is $(1.31\pm 0.05)t$, fitted by regression against time (dashed and dotted orange lines in Fig. 18c).

#### C.4.4 AOGCM-neutral *q*(*t*) and time-profile of *T*(*t*)

For $t\gg \tau _{u}$, the evolution of *T* in the two-layer model takes place on the deep-ocean timescale $\tau _{d}=(\alpha +\gamma )c_{d}/(\alpha \gamma )$ (Eq. B9). If $\alpha \gg \gamma$, $\tau _{d}\simeq c_{d}/\gamma$, which is independent of $\alpha$. This is a reasonable approximation for *MT*2 (*cf.* Eq. C47 in Sect. C.4.3). If $\tau _{d}$ is the same for all $\alpha$, and because the two-layer model is linear, a given time-profile of *F*(*t*) will produce the same time-profile of *T*(*t*) for $t\gg \tau _{u}$, for a given set of two-layer model parameters $c_{u},c_{d},\gamma$, such as the *MT*2 model uses for all AOGCMs. We assume further that a given CO_2_ scenario will produce the same time-profile of *F*(*t*) in all AOGCMs.

Given all these conditions, we expect all AOGCMs to have the same time-profile of *T* under a given CO_2_ scenario, which means we can write $T(t)=\psi (t)T_{0}$, where $T_{0}$ is a constant temperature and $\psi (t)$ a dimensionless function of time. For example, we could choose $T_{0}$ for each AOGCM as its TCR, with $\psi =T(t)/\text{TCR }$. The magnitude $T_{0}$ depends on both $\alpha$ and the magnitude of *F*, but $\psi$ depends on neither of them. Since all AOGCMs have the same time-profile, we could compute $\psi$ from any of them. For greater accuracy, we prefer to use the AOGCM mean, because the assumption that all have the same time-profile is not perfect, and because individual AOGCM timeseries contain more unforced variability.

From the AOGCM-neutral time-profile $\psi (t)$ of *T*(*t*), we can obtain *q*(*t*) for the *MT*2 model by using

C48 $$\begin{aligned} \frac{\textrm{d}T_{d}}{\textrm{d}t} = \frac{\Phi }{c_{d}} =\frac{T-T_{d}}{\tau _{d}} \end{aligned}$$

from Eq. (6) and $\tau _{d}=c_{d}/\gamma$ for $\gamma \ll \alpha$. Since *T*(*t*) is known, this equation can be solved for $T_{d}$ by considering that

C49 $$\begin{aligned} \frac{\textrm{d}}{\textrm{d}t}\left( T_{d}\exp (t/\tau _{d})\right){}& {} = \frac{\textrm{d}T_{d}}{\textrm{d}t}\exp (t/\tau _{d})\nonumber \\{} & {} \quad + \frac{T_{d}}{\tau _{d}}\exp (t/\tau _{d}) = \frac{T}{\tau _{d}}\exp (t/\tau _{d}), \end{aligned}$$

whence

C50 $$\begin{aligned} T_{d} = \frac{\exp (-t/\tau _{d})}{\tau _{d}} \int _{0}^{t} T\exp (t'/\tau _{d})\,\textrm{d}t'. \end{aligned}$$

Substituting for $T_{d}$ in Eq. (6),

C51 $$\begin{aligned}{} & {} N=c_{u} \frac{\textrm{d}T}{\textrm{d}t} + \gamma (T-T_{d}) = c_{u} \frac{\textrm{d}T}{\textrm{d}t} + \gamma T\nonumber \\{} & {} \quad -\frac{\gamma }{\tau _{d}}\exp (-t/\tau _{d}) \int _{0}^{t} T\exp (t'/\tau _{d})\,\textrm{d}t'. \end{aligned}$$

Therefore

C52 $$\begin{aligned} q = \frac{N}{T} = \frac{c_{u}}{T}\frac{\textrm{d}T}{\textrm{d}t} + \gamma -\frac{\gamma }{\tau _{d}T}\exp (-t/\tau _{d}) \int _{0}^{t} T\exp (t'/\tau _{d})\,\textrm{d}t'. \end{aligned}$$

Substituting for *T* in Eq. (C52), we obtain

C53 $$\begin{aligned}{} & {} q\simeq q_{1}^{*}\equiv \frac{c_{u}}{\psi }\frac{\textrm{d}\psi }{\textrm{d}t} + \gamma -\frac{\gamma }{\tau _{d}\psi }\exp (-t/\tau _{d})\nonumber \\{} & {} \quad \int _{0}^{t} \psi (t')\exp (t'/\tau _{d})\,\textrm{d}t', \end{aligned}$$

which does not involve $T_{0}$. Therefore all AOGCMs under a given CO_2_ scenario have the *q*(*t*) of Eq. (C53), given all the conditions of the first paragraph.

#### C.4.5 Relationships of $N_{T}$ and $H_{T}$ for 1pctCO2

To test the *MT*2 two-layer model for 1pctCO2, we simulate $N_{T}(t)$ and $H_{T}(t)$ for each AOGCM using its own *T*(*t*), and regress them at each time across AOGCMs against *T* to obtain estimates of *Q*(*t*) and *q*(*t*). These regression slopes agree with the *MT* regressions (compare solid black and dashed orange lines in Fig. 17g, h).

The two-layer model predicts that $(1/{\mathcal {A}})H_{T}/N_{T}={\textstyle \frac{1}{2}}t$ for steadily increasing forcing (Eq. B28). The AOGCM results are close to this; the slope of $H_{T}$ against $N_{T}$ across AOGCMs is about 0.55*t* (dashed and dotted blue lines in Fig. 18c) and the intercept is small (not shown).

To evaluate the *MT*2 AOGCM-neutral *q*(*t*), we use Eq. (C53) with $\psi (t)$ given by a quadratic function of time fitted to $\langle T(t)\rangle$. For years 20 onwards, Eq. (C53) nearly matches *MT*2 *q*(*t*) in 1pctCO2 (dashed orange and green lines in Fig. 17h). The values thus predicted ($q_{1}^{*}=0.38,0.26$ W m^-2^ K^-1^ at $t=70,130$ yr, green lines in Fig. 13d) are close to the regression slopes for AOGCM $N_{T}$ against *T* (thick orange and red lines).

For 1pctCO2, we can make a further approximation by assuming the zero-layer solution that $T\propto t \Rightarrow \psi \propto t \Rightarrow \textrm{d}\psi /\textrm{d}t=\psi /t$. Hence the first term in Eq. (C53) becomes $c_{u}/t$, and the third term becomes

C54 $$\begin{aligned}{} & {} -\frac{\gamma }{\tau _{d}t}\exp (-t/\tau _{d}) \int _{0}^{t} t'\exp (t'/\tau _{d})\,\textrm{d}t'\nonumber \\{} & {} \quad = -\frac{\gamma }{\tau _{d}t}\left( \tau _{d}t-\tau _{d}^{2} +\tau _{d}^{2}\exp (-t/\tau _{d}) \right) \nonumber \\{} & {} \quad =-\gamma + \frac{\gamma \tau _{d}}{t}(1-\exp (-t/\tau _{d})). \end{aligned}$$

Therefore

C55 $$\begin{aligned} q\simeq \frac{c_{u}}{t}+\frac{\gamma \tau _{d}}{t}(1-\exp (-t/\tau _{d})) \simeq \frac{c_{u}}{t}+\gamma \left( 1-\frac{t}{2\tau _{d}}\right) . \end{aligned}$$

This form for *q* is accurate only until about year 40. It is nonetheless helpful because it shows qualitatively how *q* decreases. With *MT*2 parameters, $q\simeq \gamma$ at $t\simeq 30$ year, when $c_{u}/t\simeq \frac{1}{4}\gamma$ and $t/2\tau _{d}\simeq \frac{1}{4}$ (the green line crosses the horizontal dotted black line in Fig. 17h).

### C.5 Relationship of *H* and *N* in the $MT2$ model

We have found that, for any *t* in either scenario, variation across AOGCMs is described by

C56 $$\begin{aligned} \frac{1}{{\mathcal {A}}}\frac{H_{M}}{N_{M}}=f_{i}t \quad \text{ and } \quad \frac{1}{{\mathcal {A}}}\frac{H_{T}}{N_{T}}=g_{i}t, \end{aligned}$$

where $f_{i}$ and $g_{i}$ are scenario-dependent ($i=1$ for 1pctCO4, 4 for abrupt4xCO2) but AOGCM-neutral. The time-profile of the ERF *F*(*t*) gives $f_{1}={\textstyle \frac{1}{2}}$ and $f_{4}=1$ (Eq. C37), while CMIP5&6 AOGCMs give $g_{1}\simeq {\textstyle \frac{1}{2}}$ and $g_{4}\simeq 1.31$ for $t\ll \tau _{d}$ (found in Sects. C.4.3 and C.4.5, where we explain them by comparison with the two-layer model). Hence

C57 $$\begin{aligned} \frac{H}{{\mathcal {A}}N}= & {} \frac{1}{{\mathcal {A}}}\frac{H_{M}+H_{T}}{N_{M}+N_{T}} =\frac{H_{M}+H_{T}}{H_{M}/f_{i}+H_{T}/g_{i}}t\nonumber \\= & {} g_{i}\frac{H_{M}+H_{T}}{(g_{i}/f_{i})H_{M}+H_{T}}t \nonumber \\= & {} g_{i}\frac{1+H_{M}/H_{T}}{1+(g_{i}/f_{i})H_{M}/H_{T}}t. \end{aligned}$$

In 1pctCO2, we have $g_{i}=f_{i}={\textstyle \frac{1}{2}}\Rightarrow (1/{\mathcal {A}})H/N={\textstyle \frac{1}{2}}t$, irrespective of $H_{M}/H_{T}$. This explains the high correlation and small intercept of *H*(*T*) (solid and dot-dash black lines in Fig. 18c), and accurately predicts the regression slope of *H* against ${\mathcal {A}}N$, which is $(0.52\pm 0.01)t$ in the AOGCMs (dashed and dotted black lines).

In abrupt4xCO2, $f_{4}=1$, so $g_{4}/f_{4}=g_{4}=1+\delta g$, with $\delta g=0.31$, whence

C58 $$\begin{aligned} \frac{H}{{\mathcal {A}}N} = \frac{\delta g+1+g_{4}H_{M}/H_{T}}{1+g_{4}H_{M}/H_{T}}t = \left( 1 + \frac{\delta g}{1+g_{4}H_{M}/H_{T}}\right) t. \end{aligned}$$

Since $H_{M}$ and $H_{T}$ are of similar size (Fig. 11a), we may approximate $(1/{\mathcal {A}})H/N\simeq (1+0.31/2.31)t=1.13t$, very close to the actual slope of $(1.196\pm 0.004)t$ from regression of *H* against ${\mathcal {A}}N$ (dashed and dotted red lines in Fig. 18c).

Thus the *MT* model can explain our finding $H\propto N$ across AOGCMs at any time in both scenarios, and predicts the ratio *H*/*N*, which is proportional to time in both scenarios.

### C.6 Step model

To investigate the relationship between the coefficients for *H* (Eq. C33) and for *N* (Eq. C34), we make use of the “step model”, which depends on the assumption that the climate system is linear i.e. its response to forcing is a linear function of the forcing (e.g. Good et al. 2011; Gregory et al. 2015), implying that the response to a sum of forcings equals the sum of responses to the forcings separately. Hence the time-dependence $X_{1}(t)$ of any quantity in 1pctCO2 can be calculated from $X_{4}(t)$ for the same quantity in abrupt4xCO2 according to

C59 $$\begin{aligned} X_{1}(t) = \frac{R}{F_{4\times }} \int _{0}^{t}\,X_{4}(t-t')\,\textrm{d}t', \end{aligned}$$

where $F_{4\times }$ is the radiative forcing of $\text{4 }\times \text{ CO}_{2}$ and and $R=\textrm{d}F/\textrm{d}t$ is the constant rate of increase of radiative forcing in 1pctCO2. The response at any time is the integral of the responses to all the previous forcing increments, each with its own delay. In practice we apply the formula as a sum over years, considering annual increments in *F*, rather than as an integral over time.

## Appendix D Differences in efficiency of uptake of heat and passive heat

In Sect. 2.11, we compared the OHUE in the 1pctCO2 experiment with the passive heat uptake efficiency in the FAFMIP faf-passiveheat experiment. In this appendix, we compare these with heat uptake in the FAFMIP faf-heat experiment, and consider what physical processes lead to differences.

In the faf-heat experiment (Gregory et al. 2016), which is 70 years long, a surface heat flux is added to the ocean, which is prescribed the same in all AOGCMs and years as a function of location and time of year. It is the same surface flux as for passive heat in the FAFMIP faf-passiveheat experiment (i.e. the CMIP5 ensemble-mean time-mean of the change in surface heat flux at the time of $2\times \text{ CO}_{2}$ in 1pctCO2), but in faf-heat it is not passive, and therefore forces climate change.

The added heat flux is the only external forcing in faf-heat, with the CO_2_ concentration being unchanged from the piControl. Furthermore, the SST supplied to the atmosphere model does not include the added heat, hence the surface fluxes do not respond to it. The experiment allows us to see how the ocean model components of the various AOGCMs respond differently to the same surface heat flux perturbation. Gregory et al. (2016) describe the technical implementation and complications in practice, which are not relevant to the present study.

The OHUE calculated from the final decade of faf-heat is larger than in 1pctCO2 for each FAFMIP AOGCM (green crosses lie higher than black letters in Fig. 19a). The reason for the larger OHUE is that the warming has penetrated more deeply in faf-heat after a similar length of integration. A deeper penetration occurs in faf-heat because the surface heat flux perturbation is constant, whereas in 1pctCO2 the surface heat flux begins at zero and increases steadily, so that the 1pctCO2 forcing keeps the warming surface-intensified. Note that this difference is simply a consequence of the time-profile of warming, not involving any non-linearity arising from the effect of temperature change on heat uptake.

In faf-heat, the perturbative surface heat flux is also supplied as the boundary condition to a passive tracer $\theta _{a}$, which is initialised to zero. This is the same passive tracer as $\theta _{p}$ of faf-passiveheat, but the results are different because of climate change affecting ocean transports in faf-heat. We calculate the ratio of the ocean volume-mean $\theta _{a}$ to the sea surface area-mean $\theta _{a}$ as a measure of the *added* heat uptake efficiency. The added heat uptake efficiency correlates strongly with *M* (green diamonds in Fig. 19a, $r=0.92$). However, the added heat uptake efficiency is smaller than the OHUE in faf-heat (green diamonds lie lower than green crosses). This behaviour indicates that the added heat is more confined near the ocean surface than the “real” heat.

OHUE is larger than added heat uptake efficiency because of the effect of CO_2_-induced climate change on ocean interior heat transport processes. The original ocean heat content is redistributed as a result of such changes. The global-mean effect of redistribution is to move heat downwards, cooling the surface and warming the interior (Gregory et al. 2016). Thus, the net downward transport of heat (added and redistributed combined) is more efficient than the downward transport of added heat alone.

Previous studies have shown that the *downward* redistribution is due to the weakening of *upward* transport in the unperturbed state, both by dianeutral convective mixing, and by mesoscale eddies, predominantly along sloping neutral directions, particularly in the Southern Ocean (Gregory 2000; Griffies et al. 2015; Morrison et al. 2013). These processes, which occur at small spatial scales and are parametrised in most AOGCMs, generally transport heat from the warmer/saltier ocean interior in the high latitudes, towards the cooler/fresher upper ocean surface. Surface warming increases the gravitational stability and thus inhibits upward convective mixing and upward neutral diffusive heat transport by flattening the neutral directions, with both processes leading to enhanced sequestration of heat within the ocean interior (e.g.Gregory 2000; Exarchou et al. 2015; Kuhlbrodt et al. 2015; Saenko et al. 2021.

The added heat uptake efficiency in faf-heat is smaller than the passive heat uptake efficiency in faf-passiveheat (green diamonds lie below blue diamonds). Since $\theta _{a}$ and $\theta _{p}$ have the same surface flux, their volume integrals are the same, and the difference in their distributions must be due to the effect of CO_2_-induced climate change in faf-heat on the uptake processes of $\theta _{a}$. These are the same phenomena as for redistribution of heat in faf-heat, but the outcome is different. Here, they affect the uptake of the “new” heat $\theta _{a}$, whose initial field is all zero, whereas for $\theta$ their dominant effect is redistribution of the initial field.

Comparison of the latitude-depth distributions of $\theta _{a}$ and $\theta _{p}$ (contour lines and colours in Fig. 19b) and of their vertical integrals (Fig. 19c) shows that the main difference between them is in the North Atlantic. Therefore the likely cause is the weakening of the AMOC in faf-heat. The stronger AMOC in faf-passiveheat conveys $\theta _{p}$ to greater depth, and transports some of it southwards along the deep western boundary to the Southern Ocean, whereas more of $\theta _{a}$ collects in the upper 1000 m of the Arctic. There is little difference between zonal-mean $\theta _{p}$ and $\theta _{a}$ in the low latitudes and the Southern Hemisphere (Fig. 19b). (Couldrey et al. (2023) study the effect of AMOC change on OHU in FAFMIP experiments in detail.) The difference between passive and added heat uptake efficiency is greater for AOGCMs with larger *M* (the blue line in Fig. 19a is steeper than the black–red one). This difference is consistent with the AMOC weakening more in AOGCMs that have a stronger piControl AMOC (Gregory et al. 2005; Gregory and Tailleux 2011; Weaver et al. 2012; Winton et al. 2014; Weijer et al. 2020; Couldrey et al. 2023) (Fig. 19d, $r=-0.65$ with all AOGCMs, $r=-0.79$ excluding the outliers F, T and V). The larger passive heat uptake efficiency means that $\theta _{p}$ is conveyed to greater depth than the warming in 1pctCO2, explaining why its correlation with piControl *M* has a deeper maximum (solid blue and black lines in Fig. 6a).

## Appendix E Models of global ocean heat uptake

In this appendix, we summarise for reference the structure and coefficients of the models of global OHU discussed in this paper, and we repeat the main equations. All symbols are defined in Appendix F.

The models differ in their treatments of the heat flux between the upper ocean, whose change in temperature is *T* (with respect to the unperturbed state), and the deep ocean, whose change in temperature is $T_{d}$. The latter is irrevelant in the zero-layer model (Fig. 8a). The *MT*2 model (Fig. 8c) has two routes for the heat flux from the upper to the deep ocean, one of which is formulated like the two-layer model (Fig. 8b). The ocean heat uptake efficiency $\kappa =N/T$ is a constant in the case of the zero-layer model, but in other cases it is a time-dependent quantity calculated by the model.

The rate of OHU *N*(*t*) and the OHU $H(t)=\int _{0}^{t}\,N(t')\,\textrm{d}t'$ are calculated by each of these models given *T*(*t*) for an individual AOGCM as an input. Since the models are linear, they may equally well calculate AOGCM-mean $\langle H(t) \rangle$ given $\langle T(t) \rangle$. In other words, *N* is the solution of the OHU model equations, given *T* as a boundary condition. For energy conservation, *N* and *T* must at the same time satisfy the global energy balance

1 $$\begin{aligned} N = F - \alpha T, \end{aligned}$$

which has the same form in every model, where *F* is the effective radiative forcing and $\alpha$ is the climate feedback parameter, both of which are time-dependent and differ among AOGCMs.

In the following table, “AOGCM-neutral” quantities are are the same for all AOGCMs modelled, whereas “AOGCM-specific” are different for each AOGCM. Coefficients shown with (*t*) depend on time and scenario; those without (*t*) are constants. Equation numbers correspond to equations in the main text of the paper.

## Appendix F Definitions of symbols and abbreviations

This table lists mathematical symbols, abbreviations and units for the quantities which are mentioned in the text without being defined on every appearance.

**Table:**

Symbol	Unit	Definition
${\mathcal {A}}$	m^2^	Global surface area (land and ocean together).
$c_{u},c_{d}$	J m^-2^ K^-1^	Heat capacities per unit of Earth surface area of the upper and deep ocean layers of the two-layer global ocean model, AOGCM-neutral constants in *MT*2, AOGCM-specific constants in *MT*2*T*.
EffCS	K	Effective climate sensitivity $F_{2\times }/\alpha$, the equilibrium warming which would result under $2\times \text{ CO}_{2}$ for constant $\alpha$.
*F*	W m^-2^	Effective radiative forcing (ERF).
$F_{2\times },F_{4\times }$	W m^-2^	ERF due to doubling and quadrupling CO_2_.
*H*	ZJ	Global ocean heat uptake (OHU) wrt unperturbed equilibrium climate, equal to the global time-integral of *N*.
$H_{M},H_{T}$	ZJ	*M*- and *T*-dependent contributions to *H* in *MT*2.
$H_{MT}$	ZJ	$=H_{M}+H_{T}$, estimate of *H* in *MT*2.
*M*	Sv	Strength of the Atlantic meridional overturning circulation (AMOC) in the unperturbed equilibrium climate, quantified as the maximum value of the overturning streamfunction in the North Atlantic.
$M_{0}$	Sv	Constant reference value for *M* in *MT*2.
$M'$	Sv	$=M-M_{0}$.
*N*	W m^-2^	Global-mean net heat flux into the climate system per unit of global surface area, treated in this work as equal to the global-mean net downward radiative flux at the top of the atmosphere, and to the rate of ocean heat uptake $\textrm{d}H/\textrm{d}t$ per unit of *global* (not ocean) surface area.
$N_{M},N_{T}$	W m^-2^	*M*- and *T*-dependent contributions to *N* in *MT*2.
*p*	1	$=N_{M}/F$, AOGCM-specific constant in *MT*2.
*q*	W m^-2^ K^-1^	$=N_{T}/T$, AOGCM-neutral coefficient $q(t)=\partial N/\partial T$ in *MT* and *MT*2, contribution to OHUE due to *T*-dependent heat uptake.
*Q*	ZJ K^-1^	AOGCM-neutral coefficient $Q(t)=\partial H/\partial T$ in *MT* and *MT*2.
*r*	1	Pearson product–moment correlation coefficient.
*s*	W m^-2^ Sv^-1^	AOGCM-neutral coefficient $s(t)=\partial N/\partial M$ in *MT* and *MT*2.
$s_{0}$	Sv^-1^	AOGCM-neutral constant in *MT*2.
*S*	ZJ Sv^-1^	AOGCM-neutral coefficient $S(t)=\partial H/\partial M$ in *MT* and *MT*2.
*t*	year	Time since the start of the forcing scenario.
*T*	K	Global-mean change in surface air temperature wrt the unperturbed equilibrium climate, assumed equal to the temperature change of the upper layer of the two-layer global ocean model.
$T_{d}$	K	Temperature change of the deep layer of the two-layer global ocean model wrt unperturbed equilibrium climate.
TCR	K	Transient climate response, evaluated as *T* for the time-mean of years 61–80, centred on the time of $2\times \text{ CO}_{2}$, under the 1pctCO2 scenario.
*u*	W m^-2^	AOGCM-neutral *t*-dependent contribution to the rate of OHU in the *MT* model.
*U*	ZJ	AOGCM-neutral *t*-dependent contribution to OHU in the *MT* model.
$U_{0}$	ZJ	AOGCM-neutral constant contribution to OHU in *MT*2.
$\alpha$	W m^-2^ K^-1^	Climate feedback parameter, positive for stability (the sign is arbitrary and some authors write it as negative for stability); $\alpha$ is AOGCM-specific and time-dependent.
$\gamma$	W m^-2^ K^-1^	Thermal coupling coefficient, assumed constant, between the upper and deep layers of the two-layer global ocean model, AOGCM-neutral constant in *MT*2, AOGCM-specific constant in *MT*2*T*.
$\Delta$SST	K	Change in ocean area-mean sea surface temperature (SST) wrt the unperturbed equilibrium climate.
$\theta$	K	Change in ocean temperature wrt the unperturbed equilibrium climate.
$\overline{\theta }$	K	Change in ocean volume-mean temperature wrt the unperturbed equilibrium climate i.e. volume-mean of $\theta$.
$\theta _{a}$	K	Added ocean temperature tracer in the faf-heat experiment.
$\theta _{p}$	K	Added passive temperature tracer in the faf-passiveheat experiment.
$\overline{\theta }_{p}$	K	Ocean volume-mean of $\theta _{p}$.
$\kappa$	W m^-2^ K^-1^	Ocean heat uptake efficiency (OHUE) $=N/T$, for any time or scenario. As a benchmark for AOGCMs, OHUE is conventionally evaluated for the time-mean of years 61–80, centred on the time of $2\times \text{ CO}_{2}$, under the 1pctCO2 scenario.
$\kappa _{M}$	W m^-2^ K^-1^	Contribution to OHUE due to *M*-dependent heat uptake in *MT*2, $\kappa _{M}=N_{M}/T$.
$\rho$	W m^-2^ K^-1^	Climate resistance $=F/T=\alpha +\kappa$.
$\tau _{u},\tau _{d}$	year	Characteristic timescales for change in temperature of the upper and deep layers of the two-layer global ocean model.
$\Phi$	W m^-2^	Global-mean heat flux from the upper to the deep layer of the two-layer global ocean model.
$\langle x \rangle$		Mean of any quantity *x* over CMIP5&6 AOGCMs.
